# Development and testing of the Sleep Health And Wellness Questionnaire (SHAWQ) in adolescents and university students: composite SHAWQ scores are associated with sleep problems, depression symptoms, and academic performance

**DOI:** 10.3389/frsle.2023.1188424

**Published:** 2023-11-15

**Authors:** Yng Miin Loke, Samantha Lim, A. V. Rukmini, Patricia Chen, John C. K. Wang, Joshua J. Gooley

**Affiliations:** ^1^Neuroscience and Behavioural Disorders Programme, Duke-NUS Medical School, Singapore, Singapore; ^2^Institute for Applied Learning Sciences and Educational Technology, National University of Singapore, Singapore, Singapore; ^3^Department of Educational Psychology, The University of Texas at Austin, Austin, TX, United States; ^4^National Institute of Education, Nanyang Technological University, Singapore, Singapore

**Keywords:** sleep, health, depression, anxiety, grades, academic performance

## Abstract

**Introduction:**

Sleep problems frequently arise during adolescence and early adulthood and may contribute to the onset of depression. However, few sleep health instruments have been developed for use in student populations. Here, we developed a brief sleep health questionnaire for identifying adolescents and university students with sleep problems who may be at risk of depression.

**Methods:**

In Study 1, sleep survey data in adolescents (*n* = 1,733) were analyzed by best-subsets regression to identify the strongest predictors of self-reported depression symptoms: sleep quality, daytime sleepiness, self-rated health, frequency of staying up until 3:00 am, school day sleep latency, and gender. A 6-item Sleep Health And Wellness Questionnaire (SHAWQ) was developed using these items. Students were categorized into good, fair, and bad sleep health groups based on their composite SHAWQ scores. In Study 2, the SHAWQ was tested in adolescents (*n* = 1,777) for associations with depression symptoms and excessive daytime sleepiness. In Study 3, the SHAWQ was tested in university students (*n* = 2,040) for convergent validity with instruments for measuring sleep quality and insomnia severity, and for associations with major depressive disorder symptoms and anxiety disorder symptoms. Test-retest reliability was determined in a subset of 407 students who re-took the SHAWQ several weeks later. In Study 4, we tested whether SHAWQ scores in university freshmen (cohort 1, *n* = 1,529; cohort 2, *n* = 1,488) were prospectively associated with grade point average (GPA) over their first year.

**Results:**

Across studies, SHAWQ scores were associated with higher depression and anxiety scores, excessive daytime sleepiness, lower sleep quality scores, and higher insomnia severity scores, demonstrating good convergent validity. Associations of SHAWQ scores with depression symptoms were stronger compared with anxiety symptoms. SHAWQ scores showed moderate test-retest reliability. Large effect sizes were observed for bad vs. good sleep health for all sleep and mental health variables. In both cohorts of university freshmen, students with bad sleep health had lower academic performance based on their GPA and percentile rank.

**Conclusion:**

Our findings suggest that the SHAWQ could be used to screen for students in their teens and twenties with bad sleep health who would benefit from counseling for sleep and mental health.

## 1 Introduction

Sleep is a key component of mental health and wellness. Sleep that is short, improperly timed, or low in efficiency has been linked to poorer mood and depression symptoms (Fredriksen et al., 2004; Augustinavicius et al., 2014; Lovato and Gradisar, 2014; Raniti et al., 2017; Yeo et al., 2019; Orchard et al., 2020; Short et al., 2020). Low self-reported sleep quality and high daytime sleepiness are also associated with mood disturbances and depression (Short et al., 2013; Dinis and Bragança, 2018; Tsou and Chang, 2019; Orchard et al., 2020; Boz et al., 2021; Marino et al., 2021; Shimamoto et al., 2021; Gonsalvez et al., 2022). Moreover, sleep problems are among the symptoms used for diagnosing major depressive disorder in the Diagnostic and Statistical Manual of Mental Disorders, 5^th^ edition (DSM-5) (American Psychiatric Association, 2013). There is strong overlap in symptoms associated with inadequate sleep and depression including fatigue or loss of energy, diminished ability to concentrate, and psychomotor retardation. While altered sleep may arise because of poor mental health, inadequate sleep also directly impacts mood and cognitive functioning (Short and Chee, 2019; Short et al., 2020). Hence, the relationship between sleep and mental health problems is generally viewed as bidirectional. Understanding the etiology of sleep and mood disturbances is important for developing interventions to improve health and wellness.

Sleep problems and depression symptoms often emerge during adolescence and early adulthood. Bedtimes shift later across adolescence due to a combination of biological and environmental factors (Crowley et al., 2018). A puberty-associated circadian delay and slower build-up of homeostatic sleep pressure makes it easier for adolescents to stay up late and harder to fall asleep early (Carskadon et al., 2004; Jenni et al., 2005; Taylor et al., 2005). In parallel, the increase in school workload, social commitments, and bedtime autonomy can delay and displace nocturnal sleep (Short et al., 2011; Yeo et al., 2019, 2020). Most adolescents also get up earlier than their natural wake-up time to reach school on time, resulting in chronic sleep deprivation during the school week (Minges and Redeker, 2016; Wheaton et al., 2016; Bowers and Moyer, 2017; Marx et al., 2017; Alfonsi et al., 2020). Many of these factors persist into young adulthood, when sleep opportunities during the work/school week are often restricted by late night activities and early university start times (Basner et al., 2014; Yeo et al., 2021, 2023). Sleep problems frequently coincide with mood disturbances and depression. The cumulative frequencies of insomnia and depression during adolescence are about 10% and 20%, respectively (Johnson et al., 2006; Thapar et al., 2012; Hysing et al., 2013), although estimates may vary depending on the criteria used, cultural setting, and age group. Notably, girls are about twice as likely to experience insomnia and depression compared with boys (Hyde et al., 2008; Thapar et al., 2012; Hysing et al., 2013). Depression in youth is associated with increased risk of depressive episodes and other mental disorders as an adult (Dunn and Goodyer, 2006; Fergusson et al., 2007; Rudolph and Klein, 2009). Importantly, depressive disorders contribute the most to disability-adjusted life years (a measure that accounts for premature mortality and years lived with disability) among mental disorders, with a peak that occurs in adolescence and young adulthood (Whiteford et al., 2013). It is therefore critical to identify factors that contribute to the onset of depression, including the potential role of sleep problems.

Sleep problems often precede depression. It has therefore been hypothesized that poor sleep contributes to the emergence of depression (Lovato and Gradisar, 2014; Gradisar et al., 2022). A meta-analysis of longitudinal studies in adults showed that non-depressed individuals with insomnia had a 2-fold increase in the odds of developing depression compared with individuals without sleep difficulties (Baglioni et al., 2011). Comparable findings have been reported in adolescents demonstrating that insomnia symptoms were associated with a 2-fold increase in odds of depression diagnosis within the next several years (Roane and Taylor, 2008). Epidemiologic studies have also shown that adolescents with insomnia symptoms had a 1.5-fold increase in odds of reaching survey- or interview-based thresholds for depression one year later (Luo et al., 2013; Roberts and Duong, 2014). One of the mechanisms by which poor sleep may contribute to development of depression is through its effects on mood, emotional regulation, and cognitive functioning. In healthy adolescents, exposure to insufficient sleep gives rise to reduced positive mood, increased negative mood, deficits in emotional regulation, impaired cognition, and an increase in symptoms associated with depression and anxiety (Short and Chee, 2019; Short et al., 2020). Additionally, difficulty falling asleep and nighttime awakenings provide more opportunities to worry and ruminate. Thoughts that occur prior to sleep (pre-sleep cognitions) and nighttime ruminative thinking may exacerbate sleep onset latency and amplify negative feelings and self-perceptions related to depression (Lovato and Gradisar, 2014; Orchard and Reynolds, 2018; Gradisar et al., 2022). A role for sleep problems in the development of depression is also supported by treatment studies for disordered sleep without clinical depression. Meta-analyses of cognitive-behavioral interventions (i.e., non-pharmacological interventions) in adolescents showed improvements in objective and subjective measures of sleep, as well as reduced depression and anxiety symptoms (Blake et al., 2017; Gee et al., 2019). Light therapy for adolescents with delayed sleep-wake phase disorder has also been shown to improve sleep onset difficulties and reduce repetitive negative thinking and depression symptoms (Richardson and Gradisar, 2022). Together, these studies suggest that early treatment of sleep problems may help to decrease the incidence of depressive disorders.

Multiple dimensions of sleep health are associated with depression symptoms. In broad terms, good sleep health is characterized by adequate sleep duration, appropriate sleep timing, high sleep efficiency (ease of falling and returning to sleep), sustained alertness during waking hours, and satisfaction with the quality of sleep (Buysse, 2014). While there is evidence that each of these dimensions of sleep health is associated with depression symptoms, multidimensional sleep health measures may be better at predicting mental health outcomes. This is because sleep symptoms or problems have additive effects on health (Vgontzas et al., 2009). Composite scores of sleep health reflect the combined influence of different dimensions of sleep that normally co-exist rather than treating each dimension of sleep as if it occurs in isolation. The Pittsburgh Sleep Quality Index (PSQI) is an example of an instrument that provides coverage of the primary dimensions of sleep health, even though it was conceptualized as a tool for measuring sleep problems rather than sleep health. In the original validation study (Buysse et al., 1989), PSQI global scores were much higher in patients with major depressive disorder, and subsequent studies have shown that PSQI scores are correlated with depression scores in adults and adolescents (Raniti et al., 2017; Huang and Zhu, 2020). More recently, multidimensional sleep health has been assessed using the SATED scale (satisfaction with sleep, alertness during waking hours, timing of sleep, sleep efficiency, and sleep duration) (Buysse, 2014) and its derivations including RuSATED, which considers variability in sleep timing (sleep regularity) as another dimension of sleep health. Cross-sectional studies in adults that used this framework showed that higher composite sleep health scores (i.e., better sleep health) were associated with fewer depression and anxiety symptoms (Furihata et al., 2017; Bowman et al., 2021; Appleton et al., 2022; Barham et al., 2022), lower self-reported psychological distress (DeSantis et al., 2019), and lower perceived stress (Lee and Lawson, 2021). Consistent with these findings, a prospective study of older women found that a composite measure of sleep health was associated with incidence of depression (i.e., poor sleep health preceded depression) (Furihata et al., 2017). Recent evidence suggests that the same sleep health framework can be applied to “at-risk” adolescents, in whom higher sleep health composite scores were associated with lower depression and anxiety symptoms (Dong et al., 2019). These studies show that measures of multidimensional sleep health are closely related to mental health outcomes.

The present study was performed to address the need for a short sleep health instrument that can be used to predict the co-occurrence of depression symptoms in adolescents and university students. Sleep problems are thought to contribute to the onset of major depressive disorder during adolescence and early adulthood (Gradisar et al., 2022). However, few sleep health instruments have been developed for use in student populations. Current instruments assign equal weights to the various dimensions of sleep health, even though the relative contribution of each dimension to depression and other health outcomes may vary (Buysse, 2014; Raniti et al., 2017; Dong et al., 2019). Here, we took the view that sleep health-related behaviors may be more predictive of depression if they are chosen and scored based on their strength of association with depression scores. With this view in mind, we aimed to develop and test a short questionnaire to evaluate students' sleep health and well-being. In Study 1, we developed a 6-item Sleep Health And Wellness Questionnaire (SHAWQ) by selecting for sleep survey items that were most strongly associated with depression scores in adolescents. SHAWQ scores were also used to categorize students into good, fair, and bad sleep health groups. In Study 2, we tested the SHAWQ in a different population of adolescents. We hypothesized that SHAWQ scores and categories would be associated with global depression scores and individual depression symptoms, as well as excessive daytime sleepiness. In Study 3, we tested the SHAWQ in university students. We hypothesized that the SHAWQ would exhibit convergent validity with other multidimensional instruments for assessing sleep quality and insomnia symptoms, with good test-retest reliability over several weeks. We also predicted that the SHAWQ would be more predictive of depressive disorder symptoms compared with anxiety disorder symptoms. In Study 4, we tested the SHAWQ in 2 different cohorts of university freshmen. We hypothesized that bad sleep health on the SHAWQ would be prospectively associated with lower academic performance.

## 2 Methods

### 2.1 Ethics statement

Adolescents provided written or online informed consent to participate in the research with prior permission obtained from their parent/guardian. Ethical approvals for studies in adolescents were obtained from the Institutional Review Board (IRB) at the National University of Singapore (Study 1: IRB-B-15-243) and the Nanyang Technological University (Study 2: IRB-2020-11-001-01). University students provided online informed consent to participate in the research. Ethical approvals for studies in university students were obtained from the National University of Singapore IRB (Study 3: NUS-IRB-2020-604; Study 4: NUS-IRB-L2020-06-02) and the Learning and Analytics Committee on Ethics, National University of Singapore. The research (Study 1, Study 2, Study 3, Study 4) was not pre-registered.

### 2.2 Study 1: analysis of sleep and depression symptoms in adolescents

#### 2.2.1 Participant recruitment and school characteristics in Study 1

Adolescents aged 13-19 years (*n* = 2,364) were recruited to take part in a cross-sectional anonymous survey of their sleep habits, daily activities, and depression symptoms. As reported in our previous work (Yeo et al., 2019), the main purpose of the survey was to evaluate factors that influence sleep and well-being in adolescents. Data were extracted for the present research to analyse sleep health-related variables that were associated with depression symptoms. In short, 8 schools (out of 74) agreed to participate including 5 local schools and 3 international schools. Student recruitment was managed internally by a designated school representative (e.g., professional educator/teacher). This included distributing study information to students and obtaining written permission from parents. Students were invited to attend a one-time session during their morning assembly to complete the survey by pen and paper under supervision of the researchers. One school opted to have students complete the survey online. Surveys were administered between January 2016 and July 2017 and were scheduled to avoid major examinations.

#### 2.2.2 Survey items and instruments in Study 1

The sleep habits survey comprised 40 questions and took about 15 min to complete. Most items were taken from the School Sleep Habits Survey used to assess adolescent sleep behavior (Wolfson and Carskadon, 1998). The survey collected information on (a) demographics and general student information, (b) sleep behavior and daily activities on school days, (c) sleep behavior and daily activities on non-school days (weekends or holidays), (d) sleep problems, sleepiness, and caffeine use, and (e) sleep preferences. The survey included a combination of free-response and multiple-choice questions. The 11-item Kutcher Adolescent Depression Scale (KADS) was used to evaluate depression severity over the past week (LeBlanc et al., 2002; Brooks et al., 2003). The global depression score was determined by summing scores across items (range=0 to 33) (Supplementary Methods).

#### 2.2.3 Development of the sleep health and wellness questionnaire (SHAWQ)

##### 2.2.3.1 Selection of SHAWQ survey items

**Figure 1:**
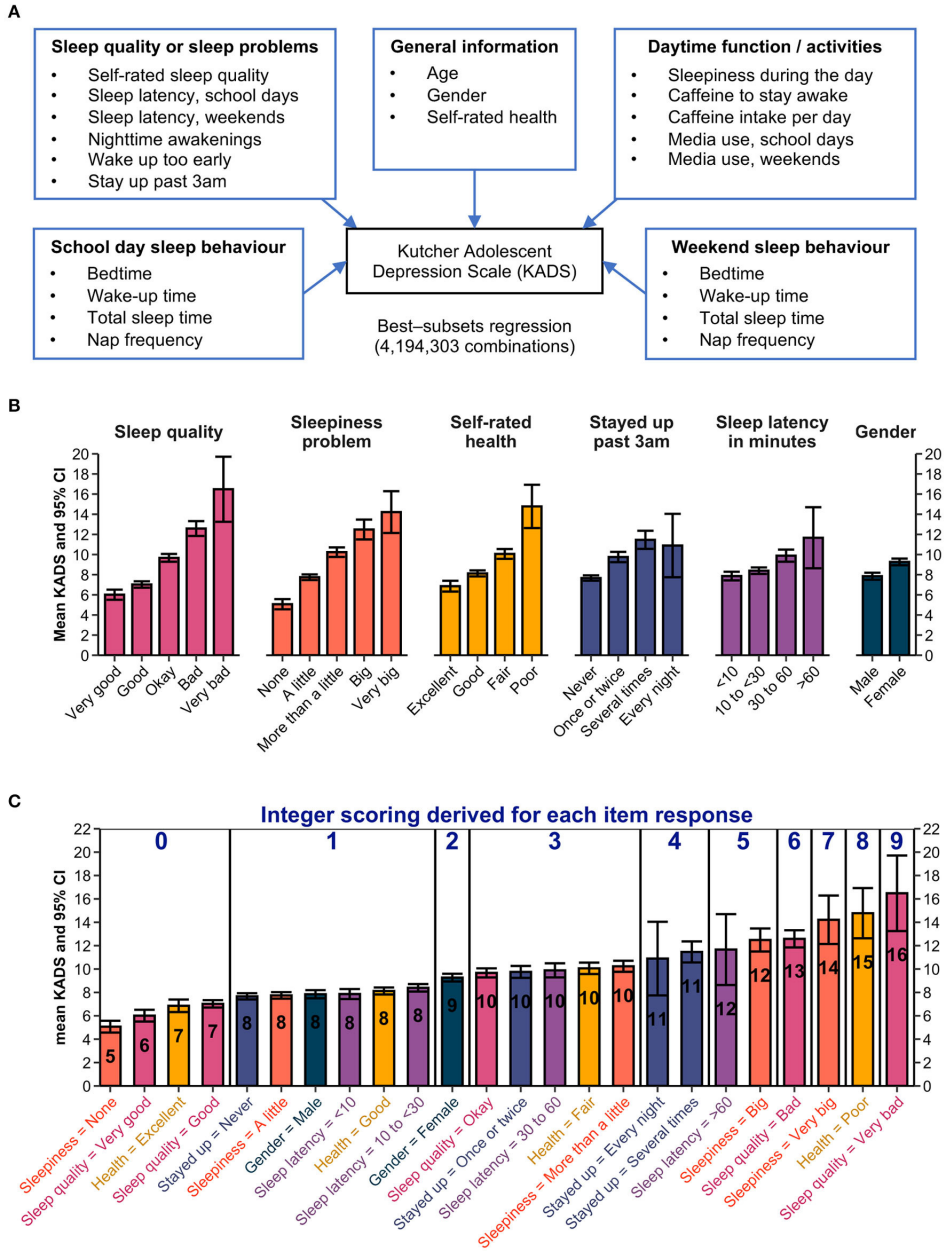
Development of the Sleep Health And Wellness Questionnaire (SHAWQ). **(A)** Best-subsets regression was used to identify combinations of sleep survey items that associated most strongly with depression scores on the Kutcher Adolescent Depression Scale (KADS). Data for 22 candidate predictors were entered in the model from 1,733 adolescents. **(B)** The best 6-predictor model included questions assessing sleep quality, daytime sleepiness, self-rated health, frequency of staying awake until 3:00 am or later in the past 2 weeks, sleep onset latency on school days, and gender. The mean and 95% CIs for KADS scores are shown for individual response items. **(C)** The integer scoring scheme is shown. Data from **(B)** were reordered by the mean KADS score. The rounded KADS score (overlaid in each bar) was converted to scores ranging from 0 to 9 by subtracting 7 for each value. Items with a rounded KADS score of 7 or less were assigned a value of zero.

We shortlisted 22 questions from the sleep habits survey that we expected to be linked to adolescents' sleep health and/or well-being (Figure 1A, Supplementary Methods). Best-subsets regression was used to identify combinations of survey items that were associated most strongly with global depression scores. This method compared all possible linear regression models that could be created based upon the identified set of predictors. With 22 survey questions shortlisted as potential predictors, there were 4,194,303 possible combinations of linear models. Free-response items were included as continuous variables, and multiple-choice questions were included as categorical variables. Among the 2,364 adolescents who participated in the survey, there were 631 individuals who were missing data for one or more of the predictor variables or the KADS. Therefore, data for 1,733 students were entered into the model (Supplementary Table 1a). The best-subsets regression model was implemented with the “olsrr” package (version 0.5.2) using R statistical software (R Core Team, 2022).

For each n-predictor model (i.e., ranging from a 1-predictor model to a 22-predictor model), we determined the set of predictors that explained the greatest amount of variance in the KADS depression score based on the R^2^ value (Table 1). The full model that included all 22 predictors explained 37.6% of the variance in KADS depression scores. The first few n-predictor models accounted for most of the variance that could be explained with marginal improvement beyond several predictors. We chose the best 6-predictor model for developing our sleep health questionnaire based on theoretical and practical considerations (not based on a statistical threshold for variable selection). The 6-predictor model explained 34.5% of the variance in KADS depression scores (Table 1). The predictor variables included self-rated sleep quality, daytime sleepiness, frequency of staying up until 3:00 am or later in the past 2 weeks, and sleep onset latency on school days. There is a strong theoretical basis for including these 4 items because they overlap with previously defined dimensions of sleep health, including sleep satisfaction/quality, daytime alertness, sleep timing, and sleep efficiency (Buysse, 2014). The other 2 predictor variables were self-rated health relative to one's peers and gender, which have been shown to associate with multidimensional sleep health and depression symptoms (Dalmases et al., 2015; Appleton et al., 2022). Lastly, we opted for the 6-predictor model because it aligned with our objective to develop a short instrument that would be practical to administer and score.

**Table 1:** Results of best-subsets regression for associations of sleep survey variables with depression score (Study 1).

**No**.	**Predictors**	**R^2^**	**Adj R^2^**
1	Sleep quality	0.195	0.193
2	Sleepiness problem, sleep quality	0.282	0.278
3	Self-rated health, sleepiness problem, sleep quality	0.311	0.307
4	Self-rated health, sleepiness problem, stay up until 3:00am, sleep quality	0.325	0.319
5	Self-rated health, wake up during night, sleepiness problem, stay up until 3:00am, sleep quality	0.337	0.330
6	Sex, self-rated health, school day sleep latency, sleepiness problem, stay up until 3:00am, sleep quality	0.345	0.339
7	Sex, self-rated health, school day sleep latency, school day media use, sleepiness problem, stay up until 3:00am, sleep quality	0.352	0.346
8	Sex, self-rated health, school day sleep latency, school day media use, wake up during night, sleepiness problem, stay up until 3:00am, sleep quality	0.358	0.350
9	Sex, self-rated health, school day sleep latency, school day media use, wake up during night, sleepiness problem, days of caffeine use, stay up until 3:00am, sleep quality	0.363	0.353
10	Sex, self-rated health, school day bedtime, school day sleep latency, school day media use, wake up during night, sleepiness problem, days of caffeine use, stay up until 3:00am, sleep quality	0.366	0.357
11	Sex, self-rated health, school day bedtime, school day sleep latency, school day media use, wake up during night, sleepiness problem, days of caffeine use, wake up too early, stay up until 3:00am, sleep quality	0.369	0.359
12	Sex, self-rated health, school day bedtime, school day sleep latency, school day media use, wake up during night, sleepiness problem, days of caffeine use, caffeine drinks per day, wake up too early, stay up until 3:00am, sleep quality	0.372	0.360
13	Sex, age, self-rated health, school day sleep latency, school day nocturnal sleep, school day media use, wake up at night, sleepiness problem, days of caffeine use, caffeine drinks per day, wake up too early, stay up until 3:00am, sleep quality	0.374	0.362
14	Sex, age, self-rated health, school day sleep latency, school day nocturnal sleep, school day media use, weekend sleep latency, wake up during night, sleepiness problem, days of caffeine use, caffeine drinks per day, wake up too early, stay up until 3:00am, sleep quality	0.375	0.362
15	Sex, age, self-rated health, school day bedtime, school day sleep latency, school day wake-up time, school day media use, weekend sleep latency, wake up during night, sleepiness problem, days of caffeine use, caffeine drinks per day, wake up too early, stay up until 3:00am, sleep quality	0.375	0.362
16	Sex, age, self-rated health, school day bedtime, school day sleep latency, school day wake-up time, school day media use, weekend sleep latency, weekend media use, wake up during night, sleepiness problem, days of caffeine use, caffeine drinks per day, wake up too early, stay up until 3:00am, sleep quality	0.376	0.362
17	Sex, age, self-rated health, school day bedtime, school day sleep latency, school day wake-up time, school day media use, weekend bedtime, weekend sleep latency, weekend media use, wake up during night, sleepiness problem, days of caffeine use, caffeine drinks per day, wake up too early, stay up until 3:00am, sleep quality	0.376	0.362
18	Sex, age, self-rated health, school day bedtime, school day sleep latency, school day wake-up time, school day naps, school day media use, weekend bedtime, weekend sleep latency, weekend media use, wake up during night, sleepiness problem, days of caffeine use, caffeine drinks per day, wake up too early, stay up until 3:00am, sleep quality	0.376	0.361
19	Sex, age, self-rated health, school day bedtime, school day sleep latency, school day wake-up time, school day naps, school day media use, weekend bedtime, weekend sleep latency, weekend wake-up time, weekend media use, wake up during night, sleepiness problem, days of caffeine use, caffeine drinks per day, wake up too early, stay up until 3:00am, sleep quality	0.376	0.361
20	Sex, age, self-rated health, school day bedtime, school day sleep latency, school day wake-up time, school day naps, school day media use, weekend bedtime, weekend sleep latency, weekend wake-up time, weekend naps, weekend media use, wake up during night, sleepiness problem, days of caffeine use, caffeine drinks per day, wake up too early, stay up until 3:00am, sleep quality	0.376	0.360
21	Sex, age, self-rated health, school day bedtime, school day sleep latency, school day wake-up time, school day nocturnal sleep, school day naps, school day media use, weekend bedtime, weekend sleep latency, weekend wake-up time, weekend naps, weekend media use, wake up during night, sleepiness problem, days of caffeine use, caffeine drinks per day, wake up too early, stay up until 3:00am, sleep quality	0.376	0.360
22	Sex, age, self-rated health, school day bedtime, school day sleep latency, school day wake-up time, school day nocturnal sleep, school day naps, school day media use, weekend bedtime, weekend sleep latency, weekend wake-up time, weekend nocturnal sleep, weekend naps, weekend media use, wake up during night, sleepiness problem, days of caffeine use, caffeine drinks per day, wake up too early, stay up until 3:00am, sleep quality	0.376	0.359

##### 2.2.3.2 Scoring of the SHAWQ

We implemented an integer-based scoring method for the SHAWQ in which scores for individual response items were based on their strength of association with the KADS depression score. We expected some questions and their individual response items would be more closely related to depression symptoms than others, and hence we did not assign equal weights. First, we determined the average KADS depression score for each response item on the SHAWQ (Figure 1B). As expected, depression scores increased with poorer sleep quality, greater severity of daytime sleepiness, higher frequency of staying up until 3:00 am or later, and longer sleep latencies. Depression scores were also higher in girls compared with boys and increased with poorer self-rated health.

**Figure 2:**
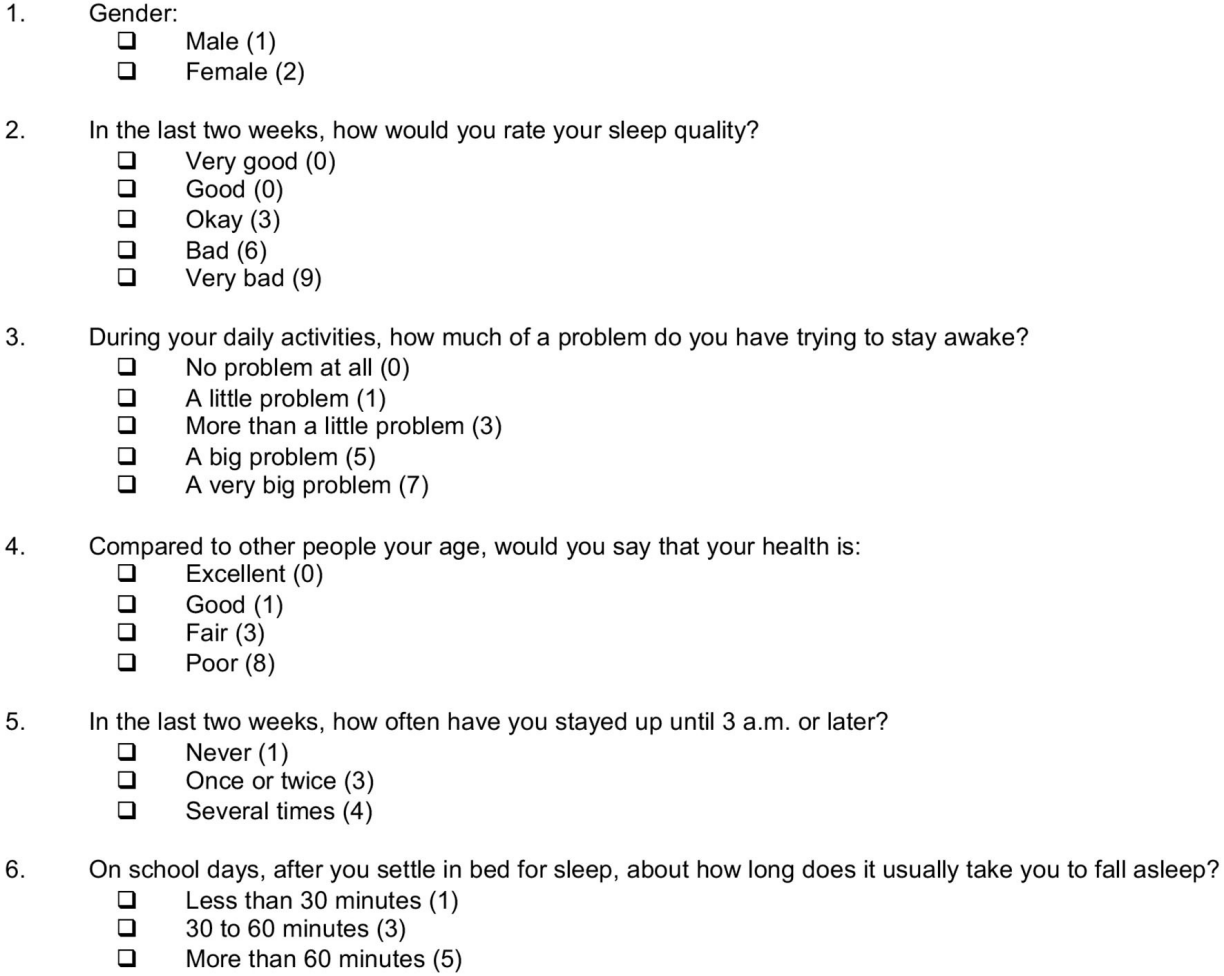
The 6-item Sleep Health And Wellness Questionnaire (SHAWQ). The SHAWQ was developed from analysis of a prior study in adolescents (Study 1, *n* = 1,733), and then tested in populations of adolescents (Study 2, *n* = 1,777), university students (Study 3, *n* = 2,040), and 2 cohorts of university freshmen (Study 4, *n* = 1,529 and *n* = 1,488). The integer scoring is indicated to the right of each response item. The composite SHAWQ score was determined by summing the 6 item scores (range = 3–35). The response item “Very Good” for sleep quality was included in Study 1 but was dropped in subsequent studies. Researchers who use the SHAWQ may decide to include this response option to balance the valence of available choices.

Next, we rounded the average KADS depression score for each response item to the nearest integer value (Figure 1C). The rounded KADS values were then converted to scores ranging from 0 to 9 by subtracting 7 from each value (any item with an average KADS score of 7 or less was assigned a score of zero). In some cases, more than one response item from a question was assigned the same score. In these instances, we reduced the number of response items on the SHAWQ by either deleting or combining response options so that each response item for a given question had a unique value. The final version of the SHAWQ used in subsequent studies had 6 questions, with 3–5 response options for each question (Figure 2).

After assigning scores to individual SHAWQ response items, we determined the composite SHAWQ score by summing scores across the 6 questions (range = 3–35) (Figure 2). SHAWQ scores exhibited a moderately right-skewed distribution with a mode of 8 and a median score of 9 (interquartile range = 7–13) (Supplementary Figure 1). Linear regression showed that the composite SHAWQ score explained 33.0% of the variance in KADS depression score (R^2^= 0.330, *p* < 0.001), which closely resembled results for the best-subsets regression analysis for the corresponding 6-item predictor model (R^2^ = 0.345, *p* < 0.001). Therefore, our simplified scoring scheme for the SHAWQ did not materially alter the strength of the association with depression scores.

Students were categorized into good, fair, and bad sleep health groups based on their SHAWQ score and its association with depression symptoms (Supplementary material). The rationale was to establish threshold scores that could be used for screening students with poor sleep health who may be at risk of mental health problems. Sleep health categories were defined by the following SHAWQ score ranges: Good, 3–7; Fair, 8–16, Bad, 17–35.

### 2.3 Study 2: assessment of the SHAWQ and depression symptoms in adolescents

#### 2.3.1 Participant recruitment and school characteristics in Study 2

Students attending secondary schools and post-secondary schools (junior college, polytechnics, vocational schools) in Singapore (*n* = 2,382) were recruited to take part in a cross-sectional anonymous survey of their sleep habits, lifestyle factors, and depression symptoms. The main purpose of the study was to identify factors that may negatively impact students' sleep and well-being. The SHAWQ was included in the survey as a potential predictor of depression symptoms. In Singapore, most students attend 4 years of secondary education in which their age of enrolment at the start of the school year is usually 13–16 years. Subsequently, adolescents can continue their education at post-secondary (pre-university) schools based on their preferences and national examination test scores. Students with high academic performance often enroll in junior college (functionally equivalent to the last 2 years of high school in the United States educational system) and go on to pursue a university education. Nearly half of students enroll in polytechnic schools (usually 3-year programs) that place greater emphasis on industry-oriented skills, hands-on learning, and work attachments that prepare them for employment in a particular job sector. Most of the remaining students enroll in Institutes of Technical Education (ITE), which provide technical and vocational pre-employment training.

Students were recruited from 10 schools including 3 secondary schools, 3 junior colleges, 1 polytechnic, and 3 ITEs. At each school, a teacher or educational administrator/leader managed student recruitment, including dissemination of study materials and obtaining parental permission. Students completed the survey by pen and paper under supervision of the researchers and/or teachers, or by filling out an online version of the survey. Surveys were administered between March 2021 and April 2022. Among the 2,382 students who consented to take part in the survey study, we excluded 201 students because they were not adolescents or they did not specify their age (≥20 years, *n* = 189; unspecified, *n* = 12). An additional 153 students were excluded because they had missing responses on either the SHAWQ or the KADS, and 251 students were excluded because they had missing data or provided invalid responses to questions on their sleep schedules. The final dataset for our analyses therefore included 1,777 adolescents (Supplementary Table 1b).

#### 2.3.2 Survey items and instruments in Study 2

The survey included the SHAWQ to assess sleep health parameters, as well as questions on nocturnal total sleep time and frequency of napping on school days and non-school days. The Munich Chronotype Questionnaire (MCTQ) was used to assess adolescents' bedtime and wake-up time on school days and non-school days (Roenneberg et al., 2003) (Supplementary material). The Epworth Sleepiness Scale (ESS) was used to measure excessive daytime sleepiness (Johns, 1991) (Supplementary material). An ESS global score >10 was interpreted as evidence of excessive daytime sleepiness (ESS score: 11–12, mild; 13–15, moderate; 16–24, severe). Depression symptoms were assessed using a 10-item version of the KADS (see above) in which the question on self-harm or suicide was removed.

### 2.4 Study 3: assessment of the SHAWQ and depression symptoms in university students

#### 2.4.1 Participant recruitment in Study 3

Undergraduate students (*n* = 2,089, aged 18 years or older) were recruited from the National University of Singapore to participate in an online survey of sleep health and wellness. The purpose of the study was to test the associations of the SHAWQ with sleep problems and depression symptoms. Students were recruited by posting advertisements on the university's learning management system and by sending emails to participants of prior research studies who consented to be re-contacted. Upon providing consent and verifying their student status, students were directed to the online survey. Among the 2,089 students who completed the survey, we excluded 6 individuals because they were listed as graduate students based on university records. There were an additional 43 students who were excluded because they had missing data for one or more items on the SHAWQ or the depression scale. Therefore, the final sample included 2,040 undergraduate students (Supplementary Table 1c). The survey was administered from April 2021 to November 2021 during the Spring and Fall semesters when school was in session. A subset of 411 students agreed to participate in the survey a second time so that we could assess test-retest reliability of the SHAWQ. These students took the survey 6-10 weeks after their first assessment (range = 41–70 days). There were four participants with at least 1 missing response on the SHAWQ. Hence, 407 students were included in the analysis of test-retest reliability.

#### 2.4.2 Survey items and instruments in Study 3

The sleep and wellness survey assessed different aspects of sleep health including those measured by the SHAWQ, sleep timing and duration on school days and non-school days (bedtime, wake-up time, nocturnal total sleep time), frequency of napping, and taking caffeine with the purpose of staying awake. The Pittsburgh Sleep Quality Index (PSQI; range = 0 to 21) was used to assess sleep quality over the past month (Buysse et al., 1989) (Supplementary material). A PSQI global score >5 was interpreted as evidence of poor sleep quality. The Insomnia Severity Index (ISI) was used to assess symptoms associated with insomnia (Bastien et al., 2001) (Supplementary material). An ISI score >14 was interpreted as evidence of clinical insomnia (ISI score: 0–7, no clinically significant insomnia; 8–14, subthreshold insomnia; 15–21, clinical insomnia, moderate severity; 22–28, clinical insomnia, severe).

Depression symptoms were assessed using the Center for Epidemiologic Studies Depression Scale Revised (CESDR) (Eaton et al., 2004) (Supplementary material). The CESDR is a 20-item scale that assesses depression symptoms across 9 dimensions that reflect the symptoms for diagnosis of clinical depression in the DSM-5. The CESDR is used to categorize respondents by their depression severity: (1) meeting the criteria for major depressive disorder, (2) probable major depressive disorder, (3) possible major depressive disorder, (4) subthreshold depression symptoms, and (5) no clinical significance. A subset of depression symptoms was also assessed using items from the KADS (LeBlanc et al., 2002; Brooks et al., 2003), including sadness, fatigue/low motivation, lack of focus, and anxiety. Anxiety disorder symptoms were assessed using the Center for Epidemiologic Studies Anxiety (CESA) scale (Faro and Eaton, 2020) (Supplementary material). The CESA is a 20-item scale that was developed as a diagnostic screening tool for detecting anxiety disorder symptoms based on clinical criteria in the DSM-5. A CESA score >16 with at least 1 response at level 3 (i.e., high severity) was interpreted as evidence of anxiety disorder symptomology.

### 2.5 Study 4: assessment of the SHAWQ and grades in university freshmen

#### 2.5.1 Participant recruitment in Study 4

University freshmen were recruited from the National University of Singapore to participate in a survey on learning beliefs, behaviors, and strategies. The main purpose of the research was to investigate associations between learner characteristics and future academic or employment outcomes. The SHAWQ was included to test whether sleep health was related to grade point average over students' first academic year. The survey was offered to all freshmen aged 18 years or older who enrolled in the Fall semester of the 2020/2021 and 2021/2022 academic years. Students were sent an email invitation which directed them to the online consent form and survey. The email distribution list was provided by the Registrar's office.

The 2020 freshman cohort completed the survey during the late part of the first semester (between October and December), whereas the 2021 freshman cohort completed the survey at the start of the academic year (between July and August). There were 1,809 students in the 2020 cohort who took part in the survey, which represented 23.4% of the freshman class. We excluded 279 students who had missing data for at least one SHAWQ item and 1 student who was not enrolled in courses. The final sample used for analyses of the 2020 freshman cohort therefore included 1,529 students (Supplementary Table 1d). There were 2,051 students in the 2021 cohort who participated in the survey, which represented 24.4% of the freshman class. We excluded 563 students who had missing data on the SHAWQ. Hence, the final sample used for analyses of the 2021 freshman cohort included 1,488 students (Supplementary Table 1e).

#### 2.5.2 Survey items and instruments in Study 4

Four items from the KADS were used to assess frequency of depression symptoms, including sadness, fatigue/low motivation, lack of focus, and anxiety (LeBlanc et al., 2002; Brooks et al., 2003). Two items from the World Health Organization Quality of Life Assessment were used to assess daytime energy and satisfaction with sleep over the past 2 weeks (World Health Organization, 2004). Participants were asked “Do you have enough energy for everyday life?” with the response options “Not at all” (1), “A little” (2), “Moderately” (3), “Mostly” (4), and “Completely” (5), and “How satisfied are you with your sleep?” with the response options “Very dissatisfied” (1), “Dissatisfied” (2), “Neither satisfied nor dissatisfied” (3), “Satisfied” (4), and “Very satisfied” (5).

#### 2.5.3 Grade point average

Permission to analyse students' survey data and grades was obtained from the National University of Singapore (NUS) Institute for Applied Learning Sciences and Educational Technology (ALSET), which stores and links de-identified student data on the ALSET Data Lake. Survey data were merged with other tables on the ALSET Data Lake including demographic information (age, sex, ethnicity, country of citizenship, academic year of matriculation, and school/faculty of enrolment), course enrolment, and course grades. Student datasets were provided by the Registrar's office and identifiers were removed by the NUS department of Information Technology before being added to the ALSET Data Lake. Each student was assigned a unique tokenized identity that could be used to link data across tables. Students provided written informed consent to add their survey data to the ALSET Data Lake for research on learning-related outcomes.

Students' course grades were analyzed over their first academic year. At NUS, the grade point for a given course module is calculated by converting letter grades into numeric values ranging from 0 to 5 (A+ or A = 5.0, A- = 4.5, B+ = 4.0, B = 3.5, B- = 3.0, C+ = 2.5, C = 2.0, D+ = 1.5, D = 1.0, F = 0.0). The cumulative grade point average (GPA) represents the average grade point weighted by the number of course credits earned in each module (i.e., modules worth more credits contribute more to the GPA). Each student's cumulative GPA was calculated after he/she completed both semesters of the freshman year. Percentile rank for GPA was also calculated separately in each freshman cohort that completed the SHAWQ. In the 2020 freshman cohort, we excluded 51 students who were enrolled in the School of Medicine or Faculty of Dentistry, which have a different method of grading compared with the rest of the university, and 1 student with missing course module information. In the 2021 freshman cohort, we excluded 52 students who were enrolled in the School of Medicine or Faculty of Dentistry, and 13 students with missing course module information. Hence, the final samples used for GPA analyses were 1,477 students in the 2020 freshman cohort and 1,423 students in the 2021 freshman cohort.

### 2.6 Analyses and statistics

#### 2.6.1 Correlational analyses of the SHAWQ with sleep and depression scores

The strength of the association between SHAWQ scores with depression scores on the KADS and CESDR was tested using Kendall's rank correlation coefficient (τ_b_, or τ, for short). Kendall's τ was also used to test associations between SHAWQ scores with daytime sleepiness scores on the ESS, sleep quality scores on the PSQI, insomnia scores on the ISI, and anxiety scores on the CESA. Kendall's τ is a nonparametric measure of correlation strength that is based on the number of concordances and discordances in the ranks of paired observations. It is an alternative to Pearson's correlation analysis for ordinal data with many tied ranks. Kendall's τ was calculated with the “stats” package (version 0.1.0) using R statistical software. The 95% CIs for Kendall's τ were determined by performing bootstrap resampling (5,000 samples) using the “boot” package (version 1.3-28.1) in R.

Results for Kendall's τ were interpreted against effect sizes reported in psychology research. A prior study investigated effect sizes across 313 psychology research studies with between-subject designs that were not pre-registered (Schäfer and Schwarz, 2019). Based on the distribution of effect sizes (Pearson's *r*), thresholds for small, medium, and large effect sizes were proposed as *r*=0.18, *r*=0.34, and *r*=0.57. In large datasets, Kendall's τ is approximately 0.67 of the value of Spearman's rho or Pearson's correlation coefficient (for variables that have an approximately normal distribution) (Sheskin, 2000). Therefore, we used the following ranges for Kendall's τ to define the relative strength of the correlation: weak association, Kendall's τ= 0.12 to 0.23; moderate association, Kendall's τ=0.23 to 0.38; strong association, Kendall's τ> 0.38.

#### 2.6.2 Test-retest reliability of the SHAWQ and other instruments

Test-retest reliability of the SHAWQ and other instruments was assessed by calculating the intra-class correlation coefficient (ICC). The ICC reflects the strength of correlation and agreement between measurements. ICC estimates and their 95% CIs were determined with the “irr” package (version 0.84.1) in R, based on a single measurement, absolute-agreement, 2-way mixed-effects model (Koo and Li, 2016). The term “single measurement” means that in practice a person's SHAWQ score would be based on a single measurement rather than the mean of multiple measurements. The term “absolute agreement” refers to the agreement between repeated measurements. A two-way mixed-effects model was used because repeated measurements are not randomized samples. The level of reliability was interpreted using the following ICC ranges (Koo and Li, 2016): poor reliability, ICC <0.50; moderate reliability, ICC between 0.50 and 0.75; good reliability, ICC between 0.75 and 0.90; excellent reliability, ICC > 0.90.

#### 2.6.3 Comparison of demographic, sleep, and mental health variables between SHAWQ categories

Chi-squared tests and ANOVA were used to test for differences in student characteristics across SHAWQ categories. Estimation statistics (effect sizes and their precision) were used to compare depression and sleep variables between SHAWQ categories (Ho et al., 2019). Estimates of population effect size with 95% CIs were determined for groups with fair sleep health or bad sleep health, assessed relative to the group with good sleep health that served as the reference. Cohen's d was used to compare standardized effect sizes for scores on the KADS, ESS, PSQI, ISI, CESDR, and CESA, where mean differences were expressed in terms of the pooled standard deviations of the samples. Relative effect size was interpreted using the following ranges for Cohen's d (Cohen, 1988): small, d between 0.20 and 0.50; medium, d between 0.50 and 0.80; large, d > 0.80.

Cliff's delta was used to compare ordinal data between SHAWQ categories, including item-by-item analyses of depression symptoms on the KADS and CESDR. Cliff's delta is a measure of ordinal dominance with values ranging from −1 to 1 that assesses the degree of overlap between populations. It measures the probability that a randomly selected member of one population has a higher response than a randomly selected member of a second population, minus the reverse probability. Relative effect size was interpreted using the following ranges: small, delta between 0.11 and 0.28; medium, delta between 0.28 and 0.43; large, delta>0.43. These effect size ranges are equivalent to those that we used for interpreting Cohen's d (see above) (Vargha and Delaney, 2000).

For each measure of effect size, the 95% CI was estimated by bootstrap resampling with 5,000 samples. The *p* value for a two-sided permutation *t*-test was reported for each comparison. It represents the likelihood of observing the effect size if the null hypothesis of zero difference is true. Estimation statistics were performed with the “dabest” package (version 0.3.1) using Python (Ho et al., 2019).

#### 2.6.4 Comparisons of grades between SHAWQ categories

GPA and percentile rank were compared between SHAWQ categories using estimation statistics (Cohen's d and mean differences) and ANCOVA. The latter was performed to assess whether SHAWQ scores were significantly associated with academic performance, adjusting for covariates including age (in years), ethnicity (Chinese, Indian, Malay, Others), country of citizenship (Singapore citizen, Singapore Permanent Resident, Foreigner), and school of enrolment (Faculty of Science, School of Business, Faculty of Engineering, Faculty of Arts & Social Sciences, School of Computing, Alice Lee Center for Nursing Studies, School of Design and Environment, Faculty of Law, Yong Siew Toh Conservatory of Music, multidisciplinary degree programs, and Others which included double degree programs, students who switched programs, and undeclared majors). We did not include sex as a covariate because this variable was included in the SHAWQ. ANCOVA was performed using the “car” package (version 3.1-1) in R. Pairwise contrasts between SHAWQ categories were performed using Tukey's test using the “emmeans” package (version 1.8.3) in R.

## 3 Results

### 3.1 Study 1 in adolescents: associations of the SHAWQ with depression symptoms

**Figure 3:**
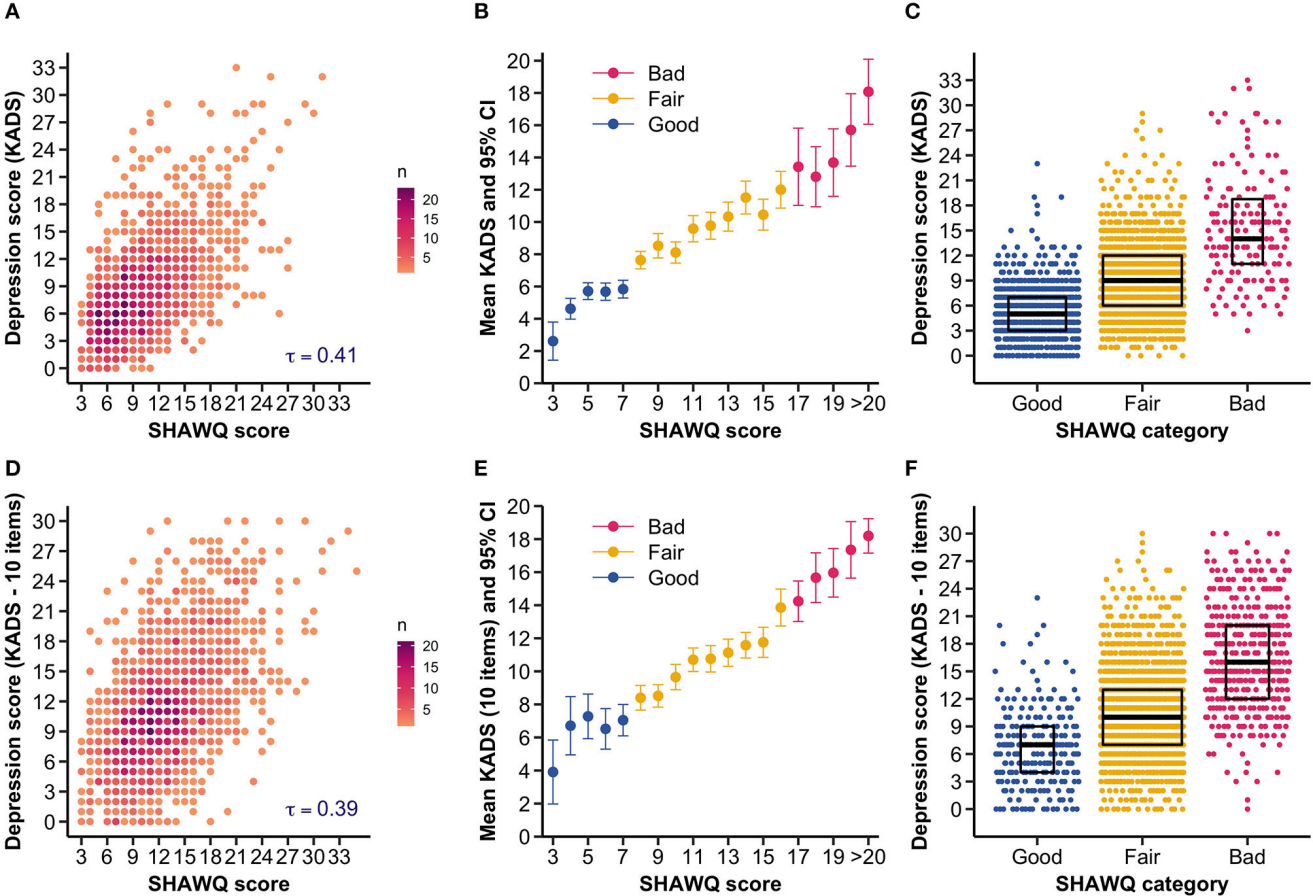
Sleep Health And Wellness Questionnaire (SHAWQ) scores were associated with depression scores in adolescents. The SHAWQ was developed from analysis of a prior study in adolescents (Study 1, *n* = 1,733) and tested in a different group of adolescents (Study 2, *n* = 1,777). In Study 1, **(A)** the scatter density plot shows that depression scores on the 11-item Kutcher Adolescent Depression Scale (KADS) increased monotonically with higher SHAWQ scores. **(B)** The average KADS score increased with the SHAWQ score. Adolescents were categorized as having as having good, fair, or bad sleep health based on the distribution of SHAWQ scores and their association with depression scores (SHAWQ score: Good = 3–7, Fair = 8–16, Bad = 17–35). **(C)** The distribution of depression scores differed across SHAWQ categories with higher scores in adolescents with bad sleep health. Box plots show the median and interquartile range. These findings were reproduced in Study 2, in which **(D)** KADS scores were positively associated with SHAWQ scores, **(E)** the average KADS score increased by SHAWQ score and category, and **(F)** the distribution of depression scores differed across SHAWQ categories. Kendall's rank correlation coefficient (τ) is shown in **(A, D)**. In Study 2, the KADS included 10 items because the question on thoughts of self-harm or suicide was removed.

**Figure 4:**
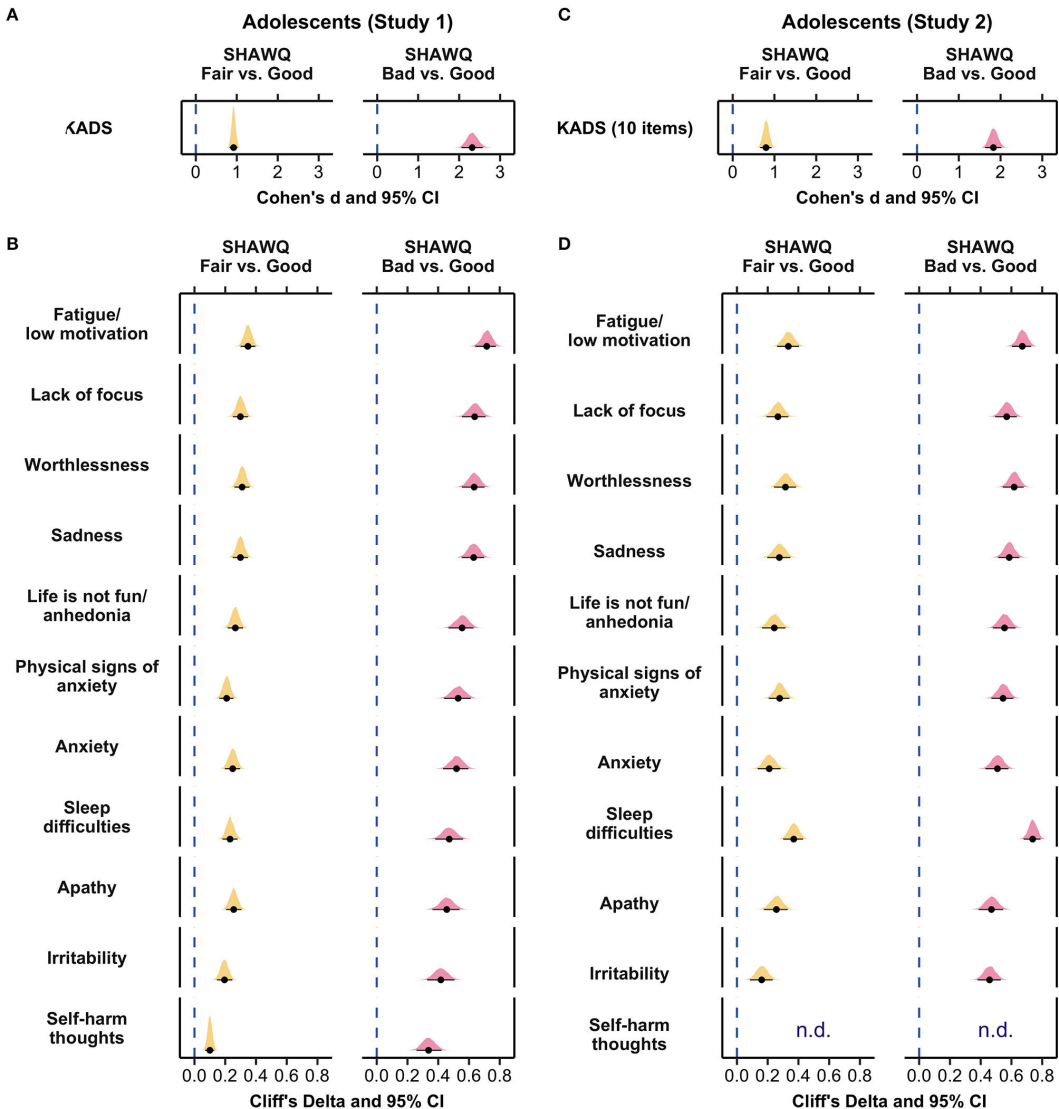
Effect size plots for associations of Sleep Health And Wellness Questionnaire (SHAWQ) categories with depression symptoms in adolescents. Adolescents were categorized as having good, fair, or bad sleep health based on their SHAWQ score. Effect sizes for fair and bad sleep health were determined relative to good sleep health. In Study 1 (retrospective study, *n* = 1,733), **(A)** large effect sizes (Cohen's d) were observed for associations of fair and bad sleep health with global depression score on the Kutcher Adolescent Depression Scale (KADS). **(B)** All depression symptoms were greater in the fair sleep health group with small-to-medium effect sizes, and in the bad sleep health group with medium-to-large effect sizes (Cliff's delta). These findings for effect sizes were reproduced in Study 2 (*n* = 1,777) for associations between SHAWQ category with **(C)** global depression scores, and **(D)** individual depression symptoms. In each plot, the population estimate of effect size is shown with 95% CIs and the bootstrap sampling distribution (5,000 samples). In Study 2, the KADS question on thoughts of self-harm or suicide was removed (n.d., not determined).

In our study of adolescents whose data were used to develop the SHAWQ, we assessed the strength of the relationship between SHAWQ scores and depression scores to establish a baseline for interpreting results of subsequent studies (see below). As expected, there was a strong monotonic association between SHAWQ score and KADS depression score (Kendall's τ = 0.406, 95% CI = 0.377–0.432, *p* < 0.001) (Figures 3A, B), and the distribution of depression scores differed substantially across SHAWQ categories (Figure 3C). Relative to students with good sleep health, the mean depression score on the KADS was about 1 standard deviation higher in students with fair sleep health (mean difference in KADS score = 3.81, 95% CI = 3.42–4.20; Cohen's d = 0.92, 95% CI = 0.82–1.02, *p* < 0.001), and >2 standard deviations higher in students with bad sleep health (mean difference in KADS score = 9.69, 95% CI = 8.70–10.81; Cohen's d = 2.32, 95% CI = 2.06–2.58, *p* < 0.001) (Figure 4A; Supplementary Table 2).

In item-by-item analyses by SHAWQ category, all depression symptoms on the KADS were higher in students with fair or bad sleep health relative to good sleep health (Figure 4B; Supplementary Table 3a). Fair sleep health was associated with medium effect sizes for fatigue/low motivation, lack of focus, worthlessness, and sadness (Cliff's delta, range = 0.30–0.35, *p* < 0.001 for all comparisons), and small effect sizes for anhedonia, physical signs of anxiety, anxiety, sleep difficulties, apathy, and irritability (Cliff's delta, range = 0.20–0.27, *p* < 0.001 for all comparisons). Bad sleep health was associated with large effect sizes for fatigue/low motivation, lack of focus, worthlessness, sadness, anhedonia, physical signs of anxiety, anxiety, sleep difficulties, and apathy (Cliff's delta, range = 0.46–0.72, *p* < 0.001 for all comparisons), and medium effect sizes for irritability and self-harm thoughts (Cliff's delta, range = 0.34–0.42, *p* < 0.001 for all comparisons) (Figure 4B; Supplementary Table 3a).

### 3.2 Study 2 in adolescents: associations of the SHAWQ with depression symptoms and excessive daytime sleepiness

#### 3.2.1 SHAWQ scores were associated with depression symptoms in adolescents

There was a strong monotonic association between SHAWQ score and depression score (Kendall's τ = 0.391, 95% CI = 0.361–0.417, *p* < 0.001) (Figures 3D, E) that closely resembled results for adolescents whose data were used to develop the SHAWQ (Figures 3A, B). There was little overlap in the distribution of depression scores between students with good vs. bad sleep health (Figure 3F), hence reproducing findings from Study 1 (Figure 3C). Relative to students with good sleep health, the mean depression score was nearly 1 standard deviation higher in students with fair sleep health (mean difference in KADS score = 3.85, 95% CI = 3.20–4.47; Cohen's d = 0.80, 95% CI = 0.66–0.93, *p* < 0.001), and nearly 2 standard deviations higher in students with bad sleep health (mean difference in KADS score = 9.77, 95% CI = 8.92–10.61; Cohen's d = 1.83, 95% CI = 1.63–2.02, *p* < 0.001) (Figure 4C, Supplementary Table 2).

All depression symptoms were experienced with greater severity in the fair and bad sleep health groups, as compared to the good sleep health group (Figure 4D; Supplementary Table 3a). Fair sleep health was associated with medium effect sizes for fatigue/low motivation, worthlessness, sadness, physical signs of anxiety, and sleep difficulties (Cliff's delta, range = 0.28–0.37, *p* < 0.001 for all comparisons), and small-to-medium effect sizes for lack of focus, anhedonia, anxiety, apathy, and irritability (Cliff's delta, range = 0.16–0.28, *p* < 0.001 for all comparisons). Bad sleep health was associated with large effect sizes for all depression symptoms (Cliff's delta, range = 0.46–0.74, *p* < 0.001 for all comparisons).

#### 3.2.2 SHAWQ scores were associated with excessive daytime sleepiness in adolescents

There was a weak monotonic increase in daytime sleepiness on the ESS with increasing SHAWQ score (Kendall's τ = 0.213, 95% CI = 0.181–0.246, *p* < 0.001) (Supplementary Figure 2). Nonetheless, there was a medium effect size of fair vs. good sleep health on ESS score (mean difference in ESS score = 2.07, 95% CI = 1.45 to 2.65; Cohen's d = 0.51, 95% CI = 0.35 to 0.66, *p* < 0.001), and a large effect size of bad vs. good sleep health in which the mean sleepiness score was about 1 standard deviation higher in students with bad sleep health (mean difference in ESS score = 4.27, 95% CI = 3.51 to 4.99; Cohen's d = 0.96, 95% CI = 0.78 to 1.13, *p* < 0.001). The proportion of students with an ESS score > 10 differed significantly across SHAWQ categories (Chi-squared = 96.3, *p* < 0.001), whereby the percentages of students categorized with excessive daytime sleepiness were 16.1%, 27.4% and 46.0% in the good, fair, and bad SHAWQ categories (Table 2).

**Table 2:** Comparison of sleep and mental health diagnostic categories between SHAWQ categories in studies of adolescents and university students.

		**SHAWQ category of sleep health**				
**Diagnostic category**	**All**	**Good**	**Fair**	**Bad**	***X** ^2^*	***p***
**Study 2 in adolescents**						
**Sleepiness on the ESS**, ***n*** **(%)**						
Normal daytime sleepiness	1,232 (70.2)	177 (83.9)	863 (72.6)	192 (54.1)	96.3	<0.001
Mild excessive daytime sleepiness	236 (13.5)	20 (9.5)	165 (13.9)	51 (14.4)		
Moderate excessive daytime sleepiness	192 (10.9)	11 (5.2)	113 (9.5)	68 (19.2)		
Severe excessive daytime sleepiness	94 (5.4)	3 (1.4)	47 (4.0)	44 (12.4)		
**Study 3 in university students**						
**Sleep quality on the PSQI**, ***n*** **(%)**						
Good sleep	1,244 (62.1)	279 (93.3)	918 (63.9)	47 (17.5)	351.9	<0.001
Poor sleep	759 (37.9)	20 (6.7)	518 (36.1)	221 (82.5)		
**Insomnia severity on the ISI**, ***n*** **(%)**						
No clinically significant insomnia	953 (46.9)	263 (87.4)	670 (45.9)	20 (7.4)	729.5	<0.001
Subthreshold insomnia	848 (41.7)	36 (12.0)	698 (47.8)	114 (41.9)		
Clinical insomnia (moderate severity)	212 (10.4)	2 (0.7)	92 (6.3)	118 (43.4)		
Clinical insomnia (severe)	21 (1.0)	0 (0.0)	1 (0.1)	20 (7.4)		
**Depression on the CESDR**, ***n*** **(%)**						
No clinical significance	1,275 (63.5)	270 (90.6)	946 (65.7)	59 (21.9)	412.4	<0.001
Subthreshold depressive symptoms	605 (30.1)	28 (9.4)	438 (30.4)	139 (51.7)		
Possible major depressive disorder	8 (0.4)	0 (0.0)	5 (0.3)	3 (1.1)		
Probable major depressive disorder	52 (2.6)	0 (0.0)	29 (2.0)	23 (8.6)		
Major depressive disorder	67 (3.3)	0 (0.0)	22 (1.5)	45 (16.7)		
**Anxiety on the CESA**, ***n*** **(%)**						
No anxiety disorder	1,535 (76.7)	265 (88.9)	1,128 (78.6)	142 (53.0)	112.2	<0.001
Anxiety disorder symptomology	466 (23.3)	33 (11.1)	307 (21.4)	126 (47.0)		

KADS, Kutcher Adolescent Depression Scale; ESS, Epworth Sleepiness Scale; ISI, Insomnia Severity Index; PSQI, Pittsburgh Sleep Quality Index; CESDR, Center for Epidemiologic Studies Depression Scale Revised; CESA, Center for Epidemiologic Studies Anxiety Scale.

### 3.3 Study 3 in university students: associations of the SHAWQ with sleep problems and mental health

#### 3.3.1 SHAWQ scores were associated with sleep quality and insomnia in university students

**Figure 5:**
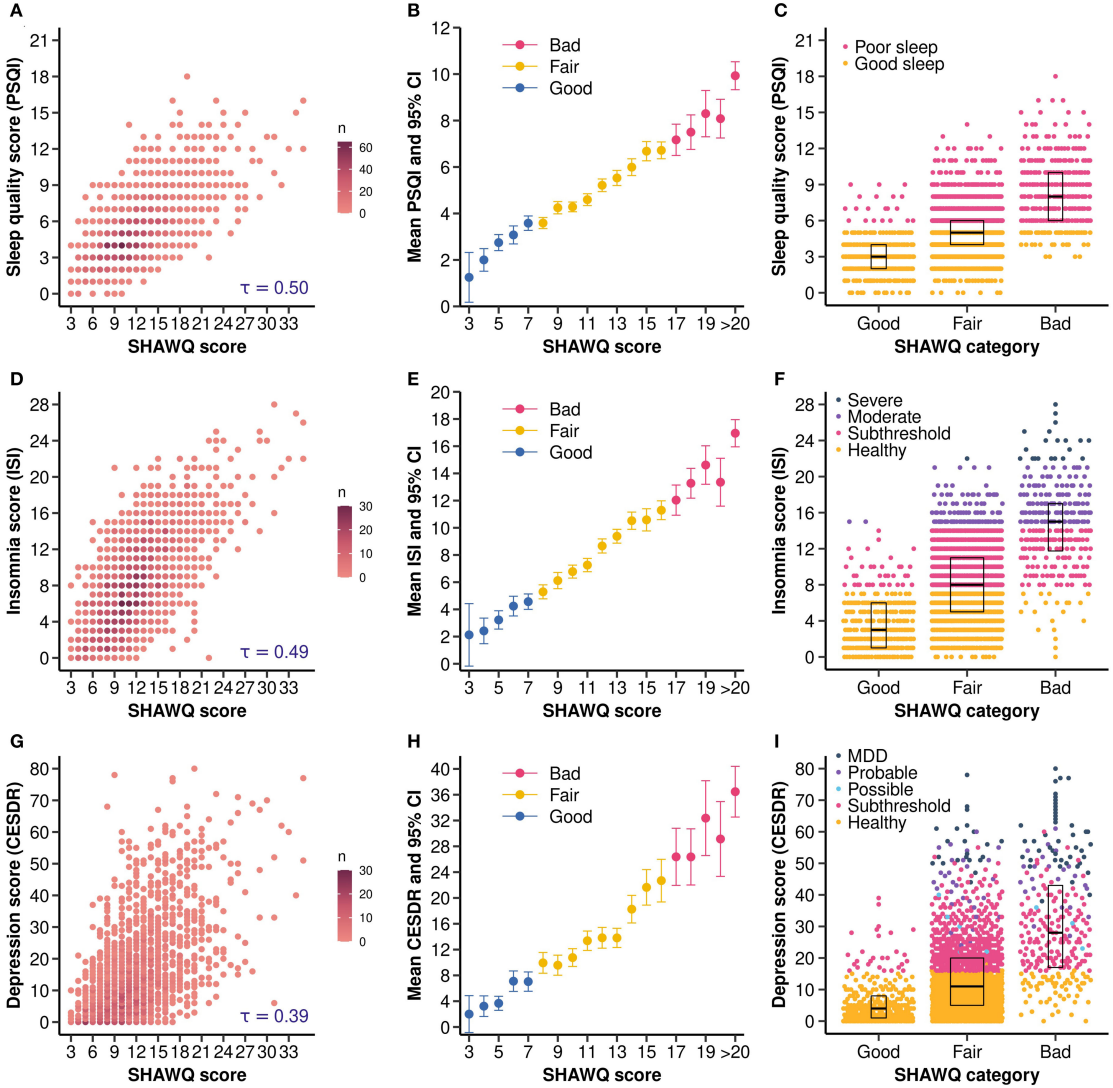
Sleep Health And Wellness Questionnaire (SHAWQ) scores were associated with sleep quality, insomnia, and depression scores in university students. University students (Study 3, *n* = 2,040) completed a survey that included the SHAWQ, the Pittsburgh Sleep Quality Index (PSQI), the Insomnia Severity Index (ISI), and the Center for Epidemiologic Studies Depression Scale Revised (CESDR). Students were categorized as having good, fair, or bad sleep health based on their SHAWQ score. **(A)** The scatter density plot shows that PSQI scores increased monotonically with higher SHAWQ scores, **(B)** the average PSQI score increased by SHAWQ score and category, and **(C)** the distribution of PSQI scores and sleep quality categories differed across SHAWQ categories. Similarly, **(D)** ISI scores increased monotonically with SHAWQ scores, **(E)** the average ISI score increased by SHAWQ score and category, and **(F)** the distribution of ISI scores and insomnia severity categories differed across SHAWQ categories. Comparable findings were observed for depression symptoms in which **(G)** CESDR scores increased monotonically with SHAWQ scores, **(H)** the average CESDR score increased by SHAWQ score and category, and **(I)** the distribution of CESDR scores and depression categories differed by SHAWQ category. Kendall's rank correlation coefficient (τ) is shown in **(A, D, G)**. Box plots in **(C, F, I)** show the median and interquartile range. MDD, Major Depressive Disorder.

Convergent validity of sleep health scores on the SHAWQ was tested against sleep quality scores on the PSQI and insomnia severity scores on the ISI. There was a strong monotonic association between SHAWQ score and PSQI score (Kendall's τ = 0.498, 95% CI = 0.473 to 0.522, *p* < 0.001) (Figures 5A, B). Secondary analyses of PSQI component scores showed moderate-to-strong associations between SHAWQ score with sleep quality (Kendall's τ = 0.501, 95% CI = 0.476 to 0.527, *p* < 0.001), daytime dysfunction (Kendall's τ = 0.365, 95% CI = 0.333 to 0.394, *p* < 0.001), sleep onset latency (Kendall's τ = 0.308, 95% CI = 0.276 to 0.341, *p* < 0.001), and sleep duration (Kendall's τ = 0.253, 95% CI = 0.219 to 0.285, *p* < 0.001), whereas weak associations were observed between SHAWQ score with sleep efficiency (Kendall's τ = 0.195, 95% CI = 0.159 to 0.230, *p* < 0.001), sleep disturbances (Kendall's τ = 0.184, 95% CI = 0.149 to 0.218, *p* < 0.001), and use of sleep medication (Kendall's τ = 0.115, 95% CI = 0.076 to 0.151, *p* < 0.001).

**Figure 6:**
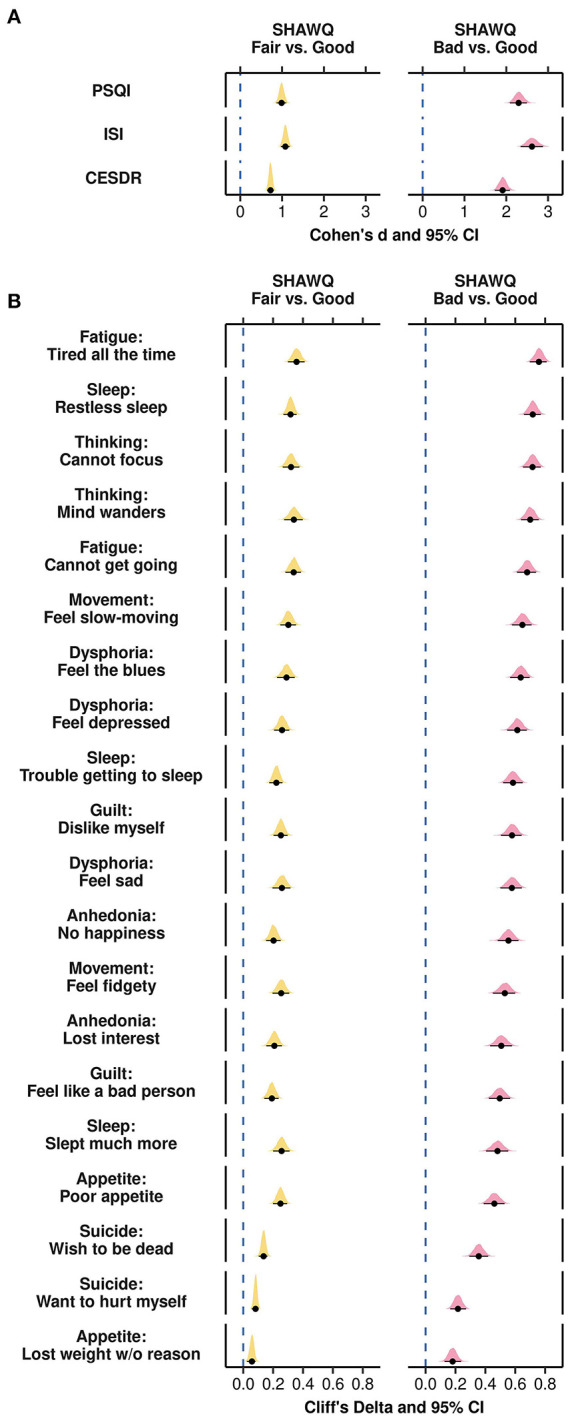
Effect size plots for associations of Sleep Health And Wellness Questionnaire (SHAWQ) categories with depression symptoms in university students. University students (Study 3, *n* = 2,040) were categorized as having good, fair, or bad sleep health based on their SHAWQ score. Effect sizes for fair and bad sleep health were determined relative to good sleep health. **(A)** Large effect sizes (Cohen's d) were observed for associations of fair and bad sleep health with scores on the Pittsburgh Sleep Quality Index (PSQI), Insomnia Severity Index (ISI), and the Center for Epidemiologic Studies Depression Scale Revised (CESDR). **(B)** All depression symptoms on the CESDR were greater in the fair sleep health group with small-to-medium effect sizes, and in the bad sleep health group with medium-to-large effect sizes (Cliff's delta). In each plot, the population estimate of effect size is shown with 95% CIs and the bootstrap sampling distribution (5,000 samples).

Large effect sizes were observed for SHAWQ category on PSQI scores (Supplementary Table 2). Relative to students with good sleep health, the mean PSQI score was about 1 standard deviation higher in students with fair sleep health (mean difference in PSQI score = 1.99, 95% CI = 1.76 to 2.21; Cohen's d = 0.99, 95% CI = 0.87 to 1.10), and more than 2 standard deviations higher in students with bad sleep health (mean difference in PSQI score = 5.37, 95% CI = 4.98 to 5.78; Cohen's d = 2.30, 95% CI = 2.09 to 2.50) (Figure 6A). There was little overlap in the distribution of PSQI scores between students with good vs. bad sleep health (Figure 5C), and the proportion of students with poor sleep quality (PSQI score>5) differed significantly across SHAWQ categories (Chi-squared = 351.9, *p* < 0.001) (Table 2). The percentages of students with poor sleep quality on the PSQI were 6.7%, 36.1% and 82.5% in the good, fair, and bad SHAWQ categories.

Sleep health scores on the SHAWQ were also strongly associated with insomnia scores on the ISI (Kendall's τ = 0.495, 95% CI = 0.469 to 0.517, *p* < 0.001) (Figures 5D, E). The mean ISI score was 1 standard deviation higher in students with fair vs. good sleep health (mean difference in ISI score = 4.23, 95% CI = 3.82 to 4.61; Cohen's d = 1.08, 95% CI = 0.97 to 1.18), and 2.6 standard deviations higher in students with bad vs. good sleep health (mean difference in ISI score = 10.51, 95% CI = 9.83 to 11.18; Cohen's d = 2.61, 95% CI = 2.34 to 2.88) (Figure 6A; Supplementary Table 2). The proportion of students with evidence of clinical insomnia on the ISI differed significantly across SHAWQ categories (Chi-squared = 729.5, *p* < 0.001) (Figure 5F; Table 2). The percentages of students with clinical insomnia on the ISI (moderate or severe) were 0.7%, 6.4%, and 50.8% in the good, fair, and bad SHAWQ categories.

#### 3.3.2 SHAWQ scores were associated with depression symptoms in university students

Sleep health scores on the SHAWQ were strongly associated with global depression scores on the CESDR (Kendall's τ = 0.393, 95% CI = 0.366 to 0.419, *p* < 0.001) (Figures 5G, H). There was a medium effect size for fair vs. good sleep health on depression score (mean difference in CESDR score = 8.19, 95% CI = 7.18 to 9.14; Cohen's d = 0.72, 95% CI = 0.64 to 0.80, *p* < 0.001), and a large effect size for bad vs. good sleep health in which the mean CESDR score was nearly 2 standard deviations higher in students with bad sleep health (mean difference in CESDR score = 25.10, 95% CI = 22.83 to 27.36; Cohen's d = 1.91, 95% CI = 1.73 to 2.09, *p* < 0.001) (Figure 6A; Supplementary Table 2). The proportion of students with evidence of major depressive disorder differed significantly across SHAWQ categories (Chi-squared = 412.4, *p* < 0.001) (Figure 5I; Table 2). The percentages of students with either probable depressive disorder or major depressive disorder were 0.0%, 3.5%, and 25.3% in the good, fair, and bad SHAWQ categories.

All depression symptoms were experienced with greater severity in the fair and bad sleep health groups compared with the good sleep health group (Figure 6B). Medium effect sizes were observed for fair sleep health for several depression symptoms related to fatigue (tired all the time; cannot get going), sleep (restless sleep), thinking (cannot focus on important things; mind wanders), movement (feel slow-moving), and dysphoria (feel the blues) (Cliff's delta, range = 0.29 to 0.36, *p* < 0.001 for all comparisons) (Figure 6B; Supplementary Table 3b). Large effect sizes were observed for bad sleep health for most depression symptoms, including those related to fatigue (cannot get going; tired all the time), sleep (slept much more; trouble getting to sleep, restless sleep), thinking (mind wanders; cannot focus), movement (feel fidgety; feel slow-moving), dysphoria (feel sad; feel depressed, feel the blues), guilt (feel like a bad person; dislike myself), anhedonia (lost interest, no happiness), and appetite (poor appetite) (Cliff's delta, range = 0.46 to 0.76, *p* < 0.001 for all comparisons). In addition, a medium effect size of bad sleep health was observed for thoughts related to suicide (wish to be dead) (Cliff's delta = 0.36, 95% CI = 0.29 to 0.42, *p* < 0.001). SHAWQ scores in university students were also associated with frequency of depression symptoms on the KADS with medium-to-large effect sizes (Supplementary Table 3b).

#### 3.3.3 SHAWQ scores were associated with anxiety disorder symptoms in university students

We tested the relationship between SHAWQ scores with anxiety disorder scores on the CESA. We predicted that the strength of the association with CESA scores would be weaker compared with CESDR scores because SHAWQ items were selected based on their correlation with depression symptoms (i.e., in Study 1 conducted in adolescents). Consistent with this prediction, SHAWQ scores were weakly associated with global anxiety score on the CESA (Kendall's τ = 0.220, 95% CI = 0.190 to 0.251, *p* < 0.001) (Supplementary Figure 3). Nonetheless, there were substantial differences in anxiety scores between SHAWQ categories. There was a small-to-medium effect size for fair vs. good sleep health on anxiety score (mean difference in CESA score = 3.34, 95% CI = 2.61 to 3.99; Cohen's d = 0.48, 95% CI = 0.38 to 0.58, *p* < 0.001), and a large effect size of bad sleep health in which the mean CESA score was about 1 standard deviation greater relative to students with good sleep health (mean difference in CESA score = 8.87, 95% CI = 7.54 to 10.31; Cohen's d = 1.09, 95% CI = 0.93 to 1.25, *p* < 0.001) (Supplementary Table 2). The proportion of students with evidence of anxiety disorder on the CESA differed across SHAWQ categories (Chi-squared = 112.2, *p* < 0.001) (Supplementary Figure 3; Table 2). The percentages of students who were categorized as having anxiety disorder symptoms on the CESA were 11.1%, 21.4%, and 47.0% in the good, fair, and bad SHAWQ categories.

#### 3.3.4 Test-retest reliability of SHAWQ scores in university students

Test-retest reliability of scores on the SHAWQ, PSQI, ISI, CESDR, and CESA was assessed in 407 university students who re-took the surveys 6–10 weeks later. SHAWQ scores showed moderate test-retest reliability (ICC = 0.644, 95% CI = 0.584 to 0.697, *p* < 0.001) that was comparable with PSQI and ISI scores for assessing sleep quality and insomnia severity, respectively (PSQI: ICC = 0.648, 95% CI = 0.588 to 0.702, *p* < 0.001; ISI: ICC = 0.659, 95% CI = 0.600 to 0.710, *p* < 0.001). The test-retest reliability of the SHAWQ was also comparable with CESDR and CESA scores used to assess depression and anxiety symptoms (CESDR: ICC = 0.684, 95% CI = 0.628 to 0.732, *p* < 0.001; CESA: ICC = 0.694, 95% CI = 0.637 to 0.743, *p* < 0.001).

### 3.4 Study 4 in university freshmen: SHAWQ scores were associated with grades

SHAWQ scores in both freshman cohorts were associated with depression symptoms on the KADS (fatigue/low motivation, sadness, lack of focus, and anxiety), as well as energy for everyday life and satisfaction with sleep on the WHO Quality of Life questionnaire (Supplementary Table 3b; Supplementary Figure 4). Bad sleep health was associated with large effect sizes for depression symptoms (Cliff's delta, range = 0.50–0.70, *p* < 0.001 for all comparisons) and quality of life measures (Cliff's delta, range = −0.88 to −0.56, *p* < 0.001 for all comparisons) (Supplementary material).

**Figure 7:**
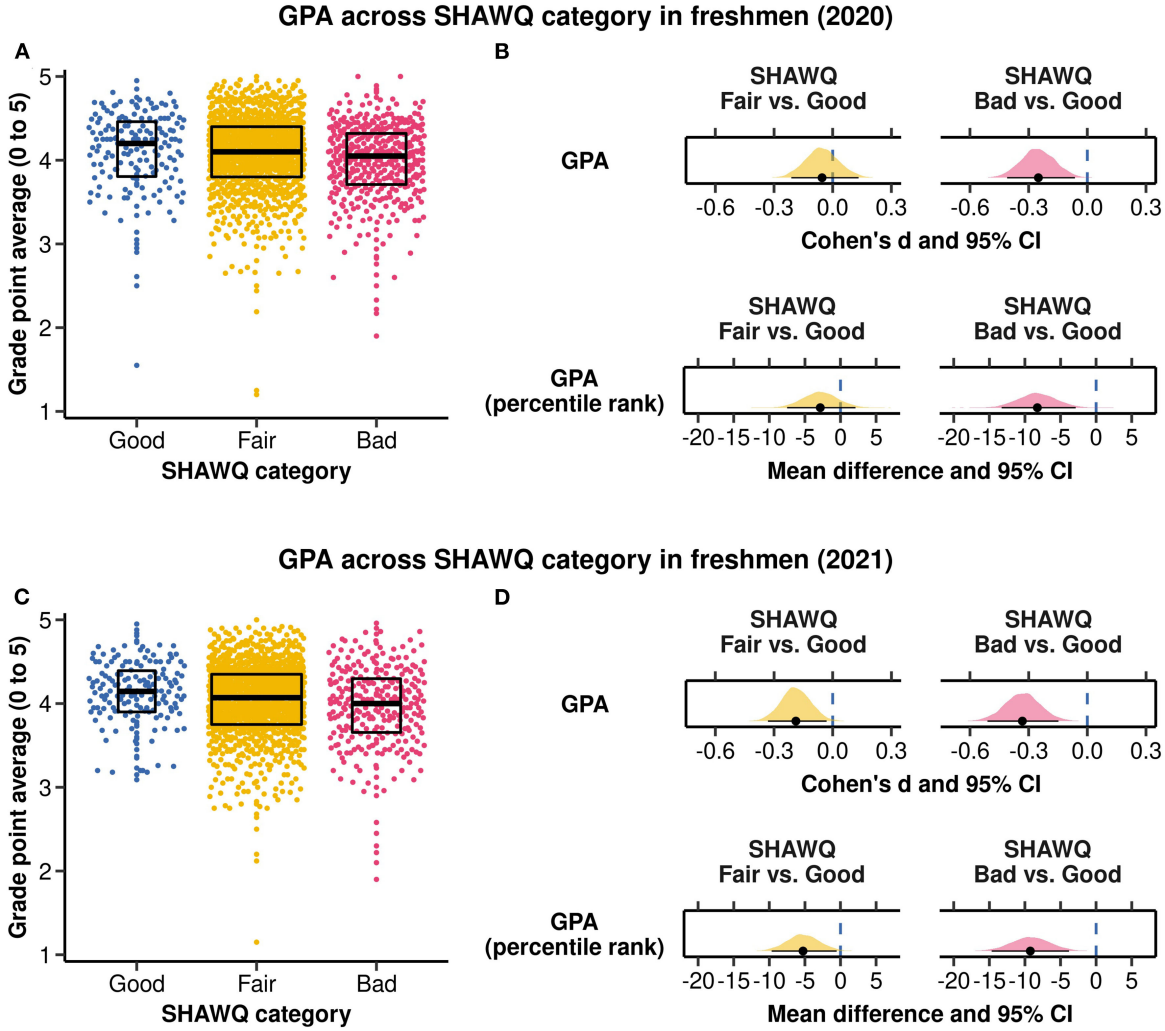
Associations of Sleep Health And Wellness Questionnaire (SHAWQ) categories with grade point average (GPA). University freshmen completed the SHAWQ near the end (2020 cohort, *n* = 1,529) or beginning (2021 cohort, *n* = 1,488) of their first semester, and their GPA was determined after the end of their first academic year spanning 2 semesters. Students were categorized as having good, fair, or bad sleep health based on their SHAWQ score. **(A)** The distribution of GPA is shown by SHAWQ category in the 2020 freshman cohort. **(B)** Effect sizes for GPA (Cohen's d) and percentile rank (mean difference) overlapped with zero in the fair sleep group, and were small-to-medium in the bad sleep health group. **(C)** The distribution of GPA is shown by SHAWQ category in the 2021 freshman cohort. **(D)** Effect sizes for GPA (Cohen's d) and percentile rank (mean difference) were small in the fair sleep health group, and were small-to-medium in the bad sleep health group. Box plots in **(A, C)** show the median and interquartile range. In **(B, D)** the population estimate of effect size is shown with 95% CIs and the bootstrap sampling distribution (5,000 samples).

In the 2020 freshman cohort, there was no difference in grade point average (GPA) in students with fair vs. good sleep health (mean difference in GPA = −0.03, 95% CI = −0.10–0.06; Cohen's d = −0.05, 95% CI = −0.21–0.13, *p* = 0.517). However, students with bad sleep health had lower grades compared with students with good sleep health based on differences in their GPA (mean difference in GPA = −0.13, 95% CI = −0.22 to −0.04; Cohen's d = −0.25, 95% CI = −0.41 to −0.06, *p* = 0.008) and differences in their percentile rank (mean difference in percentile rank = −8.26%, 95% CI = −13.27% to −2.87%; Cohen's d = −0.29, 95% CI = −0.47 to −0.10, *p* = 0.002) (Figures 7A, B; Supplementary Table 2).

In the 2021 freshman cohort, academic performance was marginally worse in students with fair vs. good sleep health for GPA (mean difference in GPA = −0.08, 95% CI = −0.15 to −0.01; Cohen's d = −0.19, 95% CI = −0.33 to −0.03, *p* = 0.028) and percentile rank (mean difference in percentile rank = −5.26%, 95% CI = −9.67% to −0.54%; Cohen's d = −0.18, 95% CI = −0.34 to −0.02, *p* = 0.029). By comparison, students with bad sleep health performed substantially worse than their peers with good sleep health for both GPA (mean difference in GPA = −0.15, 95% CI = −0.24 to −0.07; Cohen's d = −0.33, 95% CI = −0.51 to −0.15, *p* < 0.001) and percentile rank (mean difference in percentile rank = −9.24%, 95% CI = −14.67% to −3.81%; Cohen's d = −0.32, 95% CI = −0.52 to −0.13, *p* = 0.001) (Figures 7C, D; Supplementary Table 2).

ANCOVA showed that academic performance differed significantly across SHAWQ categories, adjusting for covariates (Supplementary material). Multiple comparison tests showed that the mean percentile rank of students with bad sleep health was nearly 10 percentage points lower compared with students with good sleep health, in both the 2020 freshman cohort (estimated difference = −8.24%, 95% CI = −14.35% to −2.13%; *t* = −3.16, *p* = 0.005) and the 2021 freshman cohort (estimated difference = −9.11%, 95% CI = −15.57% to −2.64%; *t* = −3.31, *p* = 0.003). Academic performance did not differ significantly between the fair and good sleep health groups (Supplementary material).

## 4 Discussion

In the present study, we developed and tested a 6–item Sleep Health And Wellness Questionnaire (SHAWQ) in adolescents and university students in their teens and twenties. Items were selected based on their combined strength of association with depression symptoms and included sleep quality, daytime sleepiness, frequency of staying up until 3:00 am or later, sleep latency on school days, self–rated health, and gender. We showed that SHAWQ scores were associated with sleep and mental health outcomes using other validated instruments. Higher SHAWQ scores (poorer sleep health) were associated with excessive daytime sleepiness, poor sleep quality, insomnia, and symptoms of major depressive disorder and anxiety. We provided evidence of convergent validity of the SHAWQ with sleep quality and insomnia severity, and moderate test–retest reliability assessed over several weeks. As expected, SHAWQ scores were more closely associated with depression symptoms compared with anxiety disorder symptoms. SHAWQ scores were used to categorize students as having good, fair, or bad sleep health. Large effect sizes were observed for bad sleep health on all sleep and mental health outcomes relative to good sleep health. Additionally, we showed that higher SHAWQ scores were prospectively associated with lower academic performance in university freshmen. Together, these findings suggest that the SHAWQ may be a useful tool for identifying students in their teens and twenties with poor sleep health who are susceptible to mental health problems and academic difficulties.

### 4.1 SHAWQ scores were associated with sleep health-related measures

Our findings suggest that SHAWQ scores can be used as a relative indicator of sleep health. Four of the items on the SHAWQ were directly related to previously defined dimensions of sleep health (Buysse, 2014), i.e., sleep quality or satisfaction with sleep, daytime alertness/sleepiness or dysfunction, sleep efficiency (ability to fall asleep and return to sleep at night), and sleep timing. Students with high SHAWQ scores also exhibited other signs of poor sleep health, as detailed in the Supplementary material, Supplementary Table 1. In all studies, students with higher SHAWQ scores had later bedtimes and shorter nocturnal sleep on school days. Additionally, students categorized as having bad sleep health were more likely to wake up in the middle of the night, wake up earlier than desired in the morning, nap on school days and weekends, and take caffeine to help stay awake during the day. The other 2 items on the SHAWQ were gender and self-rated health (discussed in more detail below). Prior studies have demonstrated marked sex differences whereby the frequencies of sleep problems and depression were about twice as high in girls compared with boys (Hyde et al., 2008; Thapar et al., 2012; Hysing et al., 2013). Self-reported health has also been shown to associate with inadequate sleep and multidimensional sleep health scores (Steptoe et al., 2006; Dalmases et al., 2015; Yeo et al., 2019).

We observed strong monotonic associations between SHAWQ scores with sleep quality scores on the PSQI and insomnia severity scores on the ISI. Although the PSQI and ISI were constructed to assess sleep problems, both instruments cover multiple dimensions of sleep health including but not limited to self-rated sleep quality, daytime functioning, and restless or disturbed sleep. Our analyses of the ESS, PSQI and ISI by SHAWQ category provided further support that SHAWQ scores provide information on students' sleep health. Among students categorized with bad sleep health on the SHAWQ, about half reported excessive daytime sleepiness and symptoms of clinical insomnia, and more than 80% had poor sleep quality on the PSQI. Bad sleep health was associated with large effect sizes for ESS scores, PSQI scores, and ISI scores, and fair sleep health was associated with medium-to-large effect sizes. Moreover, differences in ESS scores, PSQI scores, and ISI scores between good and bad SHAWQ categories exceeded minimum clinically important difference (MCID) values proposed in prior studies (MCID: ESS >2, PSQI >3, ISI >6 (Yang et al., 2009; Buysse et al., 2011; Patel et al., 2018; Crook et al., 2019), suggesting that differences in sleep problems across SHAWQ categories are clinically meaningful.

### 4.2 SHAWQ scores were associated with mental health outcomes

SHAWQ scores were strongly associated with depression scores in adolescents and university students. The strength of the association was comparable across studies despite differences in student and school characteristics (e.g., demographic and socioeconomic factors, type of school) and changes in sleep or wellbeing that may have been related to the COVID-19 pandemic (Richter et al., 2023). In all studies, bad sleep health on the SHAWQ was associated with very large effect sizes whereby the average depression score on the KADS or CESDR was nearly 2 standard deviations higher compared with good sleep health. Item-by-item analyses showed that all depression symptoms (not just those related to poor sleep) were experienced more frequently in students with fair or bad sleep health. Notably, about 1 out of every 4 students with bad sleep health reached the diagnostic threshold on the CESDR for probable depressive disorder or major depressive disorder, whereas no students with good sleep health reached this threshold. SHAWQ scores were also associated with anxiety disorder symptoms. As expected, SHAWQ scores in university students were more strongly associated with depression scores on the CESDR compared with anxiety scores on the CESA. This is presumably because SHAWQ items were selected based on their association with depression symptoms rather than anxiety symptoms. Nonetheless, major depressive disorder and anxiety disorders are highly comorbid (Kalin, 2020), and they likely co-occur in many students with poor sleep health. Hence, the SHAWQ may be useful for identifying students who are at greater risk of comorbid depression and anxiety.

Our findings are consistent with previous studies in adolescents and university students demonstrating that multidimensional sleep health was associated with mental health outcomes. For example, a study of Australian adolescents showed that higher PSQI scores (i.e., more sleep health problems) were associated with higher depression scores on the CESD and higher anxiety scores on the Spence Children's Anxiety Scale (Raniti et al., 2018). Related findings were obtained in American adolescents who were screened for evening preference and late bedtimes, in whom better composite sleep health scores derived from sleep diaries (regularity, timing, efficiency and duration of sleep) and self-report (satisfaction with sleep and daytime alertness) were associated with lower depression symptoms on the Children's Depression Rating Scale-Revised and lower anxiety symptoms on the Multidimensional Anxiety Scale for Children (Dong et al., 2019). In a study of college students, scores on the National Sleep Foundation's Sleep Health Index (SHI) were inversely related with the frequency of stress-related thoughts or feelings on the Perceived Stress Scale, and intensity of stressors experienced on the Inventory of College Students' Recent Life Experiences (Benham, 2019). Stress was most strongly associated with the sleep quality sub-index of the SHI, which includes items for self-rated sleep quality, daytime sleepiness, and difficulties falling or staying asleep. Weaker associations with stress were observed for the sleep duration sub-index, which includes questions related to time in bed (relative to age-based recommendations, self-reported sleep need, and differences between weekdays and weekends), and the sleep disorder sub-index, which includes questions related to taking sleep medications, seeking help for a sleep problem, or diagnosis of a sleep disorder.

In most studies conducted in student populations and older adults (Supplementary material), the sleep health variables that were most strongly associated with mental health were self-rated sleep quality or satisfaction with sleep, daytime alertness/sleepiness, and sleep efficiency. These were also among the top variables shortlisted for the SHAWQ. Mixed findings have been reported for the associations of sleep regularity and sleep timing with depression symptoms. In our study, staying up late (3:00 am or later) was one of the top predictors of depression scores. This result could be explained by the association of later chronotype with poorer mood and depression symptoms (Bauducco et al., 2020), or by sleep deprivation that occurs when students go to bed late but must wake up early for school (Crowley et al., 2018). Previous studies found that the correlation between sleep duration and mental health was weaker compared with other dimensions of sleep health (Furihata et al., 2017; Benham, 2019; Dong et al., 2019; Bowman et al., 2021; Lee and Lawson, 2021; O'Callaghan et al., 2021; Appleton et al., 2022), despite substantial evidence that either short or long sleep duration is associated with depression symptoms (Zhai et al., 2015; Dong et al., 2022). This could explain why sleep duration was not selected in our best-subsets regression analysis, as it did not explain additional variance in depression scores beyond other sleep survey variables. The items selected for the SHAWQ and its weighted scoring scheme are therefore consistent with prior studies showing that the various dimensions of sleep health differ in their strength of association with depression scores.

### 4.3 SHAWQ scores were associated with lower academic performance

We found that university freshmen with bad sleep health on the SHAWQ had lower grades during their first academic year compared with their peers. In 2 freshman cohorts, the GPA percentile rank was nearly 10 percentage points lower in students with bad sleep health. Although our study does not demonstrate a causal relationship between poor sleep health and grades, SHAWQ scores may provide a relative indication of how students will perform in the future. Possible mechanisms linking SHAWQ scores with grades include effects of poor sleep health on attention and learning, mental health and wellbeing, effort and motivation, as well as psychosocial factors that influence sleep and learning habits (e.g., self-regulation, family support, socioeconomic status) (Lo et al., 2016; Cousins and Fernández, 2019; Dorrian et al., 2019; Massar et al., 2019; Short et al., 2020; Tomaso et al., 2021). Importantly, the effect sizes for the association of bad sleep health with grade point average and percentile rank were within the range considered meaningful for academic outcomes (i.e., Cohen's d >0.20, with a medium effect size defined as *d* = 0.40) (Hattie, 2015; What Works Clearinghouse, 2020). Interventions for improving sleep health could therefore have a positive impact on grades if these variables are causally related.

Our findings are consistent with previous studies in adolescents and university students demonstrating that different dimensions of sleep health were associated with academic performance (Supplementary material). Composite scores relating to sleep health have also been shown to associate with academic outcomes. In university students, sleep quality scores derived from items on sleep onset latency, nocturnal awakenings, and the quality and depth of sleep were associated with end-of-semester grades (Gomes et al., 2011). Similarly, in a study of more than 55,000 students attending 4-year colleges in the United States, composite scores of sleep problems that included items on restorative sleep, difficulties with falling and returning asleep, and feeling sleepy were associated with lower self-reported grades (Hartmann and Prichard, 2018). Poorer sleep quality scores on the PSQI and higher insomnia scores on the ISI have also been shown to associate with lower academic performance in adolescents and university students (Orzech et al., 2011; Lemma et al., 2014; Adelantado-Renau et al., 2019; Carrión-Pantoja et al., 2022; Zhang et al., 2023). Our study using the SHAWQ provides further evidence that poor sleep health is correlated with grades and might be useful for identifying students at greater academic risk.

### 4.4 Comparison of the SHAWQ with other instruments

The content of the SHAWQ overlaps with other questionnaires used to assess sleep problems and sleep health. This includes the PSQI and ISI which were developed as clinical tools but are widely used in non-patient populations (Buysse et al., 1989; Bastien et al., 2001). Like the SHAWQ, the PSQI and ISI include items on sleep quality (or sleep satisfaction), sleep latency (or difficulty falling asleep), and daytime sleepiness or dysfunction. However, the PSQI also has items relating to sleep duration, sleep disturbances, and use of sleep medications, and the ISI has items on sleep maintenance, how noticeable the sleep problem is to others, and the level of worry or distress about the problem. The SHAWQ also has similarities with scales for “sleep disturbances” and “sleep-related impairment” in the National Institutes of Health Patient-Reported Outcomes Measurement Information System (PROMIS) (Cella et al., 2007). These PROMIS scales provide coverage of sleep disturbances, sleep quality, daytime dysfunction, restful sleep, and difficulty falling or staying asleep. Recently, there has been a shift toward assessing (and defining) sleep health in non-clinical populations. The Sleep Health Index (SHI) was developed by a task force at the National Sleep Foundation for assessing general sleep health (Knutson et al., 2017). As described above, the SHI conceptualizes sleep health as comprising 3 domains related to sleep quality, sleep duration, and disordered sleep. The RuSATED, on the other hand, builds on the SATED framework for sleep health and comprises six dimensions of sleep health including sleep regularity, satisfaction with sleep, daytime alertness, sleep timing, sleep efficiency, and sleep duration (Buysse, 2014). Scores on the RuSATED and SATED have been shown to associate with a broad spectrum of mental and physical health outcomes and can potentially be modified for use in children and adolescents (Dong et al., 2019; Meltzer et al., 2021). All the aforementioned instruments have advanced research on sleep health but they differ in their conceptualization (theoretical basis or intended application) and their composition or form (definitions of components or domains, number and types of questions selected). The sleep health questionnaire chosen for a given study will therefore depend on the goals of the research and the study design.

There are several ways that the SHAWQ differs from prior instruments used for assessing sleep health and/or disordered sleep. The SHAWQ was developed for use in student populations whereas most prior work has focused on adults. Given that sleep problems and depression symptoms often emerge during adolescence and early adulthood, it is important to develop and implement sleep health assessment tools during this critical period of development. While sleep health instruments in adults could be adapted for use in student populations, we selected for items in the SHAWQ that were derived from prior adolescent sleep surveys. Hence, some questions on the SHAWQ may better reflect the social context of sleep in students. For example, the SHAWQ included an item for sleep latency on school days, as well as an item on the frequency of staying awake until 3:00 am or later. Difficulty falling asleep is the most common sleep problem in adolescents (i.e. more problematic than nocturnal awakenings and returning to sleep) (Gradisar et al., 2022) and may be worse on school days when students attempt to adjust their bedtime to their school schedule. Adolescents and university students are also more likely to stay up late (i.e., past 3:00 am) compared with working adults due to biological and psychosocial factors (Carskadon, 2011; Crowley et al., 2018). These sleep variables in the SHAWQ may therefore be especially relevant for sleep health in student populations in their teens and twenties.

We did not compare the SHAWQ with other sleep instruments developed for use in adolescents. As reviewed elsewhere (Lewandowski et al., 2011; Ji and Liu, 2016; Van Meter and Anderson, 2020), there are validated questionnaires in pediatric populations for assessing daytime sleepiness, sleep hygiene and bedtime routine, sleep-related attitudes or cognitions, sleep initiation and maintenance, and disordered sleep. Many of these instruments focus on a specific domain or dimension of sleep behavior, whereas some provide broader coverage of items related to sleep health or sleep quality. It is possible that scores on these sleep questionnaires may associate with depression scores with similar strength as the SHAWQ, even though items were not selected based on their relationship with depression symptoms. Future studies should test whether the SHAWQ performs as well as, or better than, other sleep questionnaires at predicting depression symptoms in adolescents (e.g., the Adolescent Sleep Wake Scale, Adolescent Sleep Hygiene Scale; Pediatric Daytime Sleepiness Scale, Chronic Sleep Reduction Questionnaire), despite having fewer items (Drake et al., 2003; LeBourgeois et al., 2005; Meijer, 2008).

Previous sleep health instruments were developed based on conceptual grounds to include items that were viewed as distinct (i.e., representing different dimensions or constructs) and relevant for overall health and wellness (Buysse, 2014; Knutson et al., 2017). Composite scores were then used to test for associations with different health outcomes. Here, we took the opposite approach and started with the health domain that we viewed as of greatest concern in adolescents and young adults (i.e., mental health), and then used best-subsets regression to select for the combination of sleep survey items most strongly associated with depression scores. We also implemented a different scoring method that reflected the strength of association for individual response items with depression scores. Additional studies are needed to assess whether our weighted scoring approach performs better at predicting mental health outcomes compared with other instruments in which each dimension of sleep health is given equal weight.

In contrast to previous work (Buysse, 2014; Knutson et al., 2017), we did not limit the SHAWQ to questions directly related to the experience of sleep. Our primary goal was to develop a questionnaire that can be used to screen for students with sleep health problems who may be at risk of depression. We think that including gender in the SHAWQ is justified because female gender is a predictor or risk factor of sleep problems and depression (Hyde et al., 2008; Thapar et al., 2012; Hysing et al., 2013). We view gender in the SHAWQ as analogous to body mass index (BMI) in the Berlin Questionnaire (Netzer et al., 1999), where BMI is not a symptom of obstructive sleep apnoea but is associated with disease risk. Self-rated health, on the other hand, is closely related to the definition of sleep health in the SATED/RuSATED framework (Buysse, 2014), i.e., “Sleep health is a multidimensional pattern of sleep-wakefulness, adapted to individual, social, and environmental demands, that promotes physical and mental wellbeing”. The “health” component of “sleep health” implies that a person's self-perceived health and wellbeing is integral to the concept of sleep health itself. Hence, including an item on self-rated health on the SHAWQ is consistent with how sleep health is conceptualized, even though it is not specific to how sleep is experienced.

The main caveat of including gender and self-rated health in the SHAWQ is that these variables are predictors, rather than dimensions of sleep health. We do not think that this precludes the use of the SHAWQ for estimating sleep health in student populations (e.g., categorizing students into good, fair, or bad sleep health groups) because the SHAWQ provides coverage of 4 dimensions of sleep health and the composite score was strongly associated with PSQI and ISI scores. In exploratory analyses, removing the SHAWQ items on gender and self-rated health did not alter the strength of association (Kendall's τ) between SHAWQ scores with PSQI and ISI scores (Supplementary Table 4). Additionally, the strength of association with depression scores on the KADS and CESDR was only slightly weaker if SHAWQ scores were calculated using just the 4 sleep-related items rather than all 6 items (Supplementary Table 4). Hence, the association between the 6-item SHAWQ and depression scores was largely attributable to the association between sleep health and depression, rather than being explained primarily by gender and self-rated health. Future studies could assess whether the SHAWQ can be reduced to its 4 sleep-related items (with a new categorization scheme for good, fair, and bad sleep health) without substantially altering effect sizes for associations with sleep problems and depression symptoms. At present, we recommend using all 6 items on the SHAWQ because they were selected based on their combined strength of association with depression symptoms, and our findings on convergent validity, test-retest reliability, effect sizes, and prospective associations with grades are based on the 6-item SHAWQ.

### 4.5 Limitations and considerations

There are several limitations associated with the development of the SHAWQ. The items considered for the SHAWQ were limited to those used in our initial sleep habits survey that was largely based on the School Sleep Habits Survey (Wolfson and Carskadon, 1998). These questions covered the main dimensions of sleep health described in prior work (except for sleep regularity), but it is possible that including other questions on sleep habits or sleep health would have explained additional variance in depression scores. We provided initial evidence of convergent validity and test-retest reliability, but more psychometric testing is needed to assess the validity and reliability of the SHAWQ. Convergent validity should be further tested by comparing the SHAWQ with instruments that were developed for assessing sleep health in non-clinical populations such as the SHI or RuSATED. Divergent validity should be tested by comparing the SHAWQ with sleep questionnaires that were designed to measure constructs other than sleep health (e.g., sleep hygiene or bedtime procrastination), as well as behaviors/disorders other than depression or anxiety (e.g., externalizing behaviors or substance abuse). Another limitation of our study is that we did not assess internal consistency (Supplementary material), and generalizability was assessed only for student populations within Singapore.

All sleep and mental health measures in our study were based on questionnaires. No clinical interviews or clinically-verified diagnostic information were collected. Additional studies are needed to test whether the SHAWQ can be used to screen for students with sleep disorders or psychiatric disorders. We did not attempt to determine optimal classification thresholds for SHAWQ scores (e.g., receiver operating characteristic (ROC) curve analyses) because there are no normative data in Singapore for interpreting results of the various sleep instruments (ESS, PSQI, and ISI) and depression/anxiety instruments (KADS, CESDR, CESA). Students were categorized into groups (e.g., excessive daytime sleepiness, poor sleep quality, insomnia, major depressive disorder, anxiety disorder) using thresholds commonly used for these instruments, but there may be cross-cultural differences in the way that sleep and mental health problems are reported and diagnosed. Notwithstanding this issue, we found that SHAWQ scores were correlated with daytime sleepiness scores, sleep quality scores, insomnia scores, depression scores, and anxiety scores, indicating that poor sleep health on the SHAWQ was associated with worse self-reported sleep and mental health.

The SHAWQ was developed with brevity in mind and is not intended to provide a comprehensive assessment of sleep health. There were sleep survey items not selected for the SHAWQ that are clearly relevant for sleep health. These include sleep duration, nocturnal awakenings, waking up too early, and napping. Previously, we showed that shorter sleep duration on school days was associated with higher depression scores in the same population of adolescents used to develop the SHAWQ (Yeo et al., 2019). In the present study, sleep duration was not included in the SHAWQ because it did not explain additional variance in depression scores beyond those items that were already selected in the best-subsets regression analysis. In any study that seeks to comprehensively assess sleep health, we strongly recommend that other questions are included to measure sleep duration and other aspects of sleep behavior. Future iterations or refinements of the SHAWQ could include questions related to sleep duration or sleep regularity, even if they do not contribute to the SHAWQ score the way that it was defined in the present study in which depression symptoms were the main health outcome. Relatedly, any study that seeks to assess depression or other mental health problems in student populations should include measures designed for this purpose, e.g., structured interviews and validated depression questionnaires. The SHAWQ was developed with the goal of identifying students with sleep health problems that are linked to depression symptoms. The SHAWQ score provides a relative indication of a student's sleep health (good, fair, bad), but it does not measure depression.

As highlighted earlier, it is possible that the SHAWQ could be reduced while still providing a useful measure of sleep health that can predict depression. Our decision to use a 6-item instrument was guided in part by theoretical considerations, including providing coverage across 4 different dimensions of sleep health. In our best-subsets regression model used to develop the SHAWQ, however, sleep quality and daytime sleepiness were the variables that accounted for the greatest proportion of variance in depression scores. Future studies could test whether these 2 items are sufficient for estimating sleep health (e.g., relative to the 6-item SHAWQ, RuSATED, and SHI) and for predicting depression symptoms in student populations.

Our approach for selecting items on the SHAWQ was sample-dependent, i.e. based on sleep survey data in one population of adolescents (Study 1). Although the SHAWQ was subsequently tested for validity and reliability in other student populations (Study 2, Study 3, Study 4), we did not test whether the 6 items in the SHAWQ represented the best items for predicting depression symptoms across samples. An alternative and rigorous statistical approach for selecting sample-independent survey items would be to use item response theory. This approach has been used as part of PROMIS to develop item banks for sleep disturbance and sleep-related impairment which have been validated in adults and adolescents (Buysse et al., 2010; Forrest et al., 2018; van Kooten et al., 2021). Similar methods could be used to develop item banks for assessing sleep health; however, additional analyses would be required to assess which items are most closely associated with depression symptoms. Another limitation of our study is that we did not evaluate the dimensionality of the SHAWQ. Factor analysis could be performed to assess the underlying structure of variables in the SHAWQ and to test whether the results are consistent with the hypothesized (theory-based) structure. As shown in prior work, however, the factor structure of commonly used sleep instruments (e.g., PSQI, ISI, RuSATED) often differs between study populations (Chen et al., 2015; Brandolim Becker et al., 2018; Manzar et al., 2018, 2021; Ravyts et al., 2021), which could be explained by sociocultural differences in sleep health and sleep-related behaviors.

### 4.6 Future directions and potential applications of the SHAWQ

More studies are needed to replicate and extend our work demonstrating that the SHAWQ carries information relevant for students' sleep health and mental well-being. One of the first steps is to test the generalizability of the SHAWQ in student populations outside of Singapore. Sleep behavior has been shown to differ in Asian student populations, where bedtimes are often later and nocturnal sleep is shorter compared with Western cultures (Olds et al., 2010; Gradisar et al., 2011; Ong et al., 2019). The social and environmental factors that influence sleep health and wellbeing may be context-specific. Hence, it will be important to confirm the relationship between SHAWQ scores and depression symptoms in students in other cultural settings. Future studies should also investigate whether SHAWQ scores are associated with measures of social health, defined broadly as the ability to form healthy and positive relationships with others. Poor sleep health may erode the quality of social interactions with family, friends and teachers through its effects on mood and emotional regulation (Gordon et al., 2017, 2021). Conversely, students with stronger socioemotional support from their family and community may be in a better position to cultivate and maintain healthy sleep behaviors (Hale et al., 2020). While the SHAWQ was developed with sleep and mental health outcomes in mind, future work should also assess whether SHAWQ scores are associated with lifestyle behaviors relating to dietary intake and exercise. Poor sleep health may be a contributing factor to poor food choices, increased caloric intake, and sedentary activity (Beebe et al., 2013; Stea et al., 2014; Lin et al., 2018; Van Dyk et al., 2018).

The RuSATED/SATED framework proposes that definitions of sleep health should focus on measurable characteristics of sleep. Recent studies have therefore incorporated actigraphy-based measures of sleep duration, efficiency, timing, and regularity in their assessment of sleep health and its associations with mental health (DeSantis et al., 2019; Bowman et al., 2021; Lee and Lawson, 2021). While self-reported sleep quality does not have a clear biological correlate, the other measures on the SHAWQ including daytime sleepiness, frequency of staying awake until 3:00 am or later, and sleep latency can be quantified and verified with objective data. For example, daytime alertness can be evaluated by performance testing (e.g., by computer, tablet, or smartphone) or by indirect indicators such as actigraphy-verified naps. Actigraphy monitoring can also be used to assess the frequency of late bedtimes and to estimate sleep latency. Such studies are important for establishing that students' scores on the SHAWQ reflect their objectively-determined sleep health behaviors.

There are several ways that we envisage the SHAWQ could be used by schools and researchers. The SHAWQ could be used to screen for students who are at greatest risk of sleep and mental health problems. This was the primary motivation behind developing and testing the SHAWQ in the present study. Educational institutions that have limited resources to provide students with sleep counseling and mental health care could use the SHAWQ to better direct their resources to students with bad sleep health. The SHAWQ may be especially useful in sociocultural contexts where there is stigma associated with depression and other mental health problems. Negative attitudes and misconceptions of mental illness are common among adolescents and university students (Pang et al., 2017; Goh et al., 2021), and schools may be reluctant to administer depression scales to their students due to cultural sensitivities and parental concerns. Students with depression symptoms may therefore avoid seeking help and remain undiagnosed (Vaingankar et al., 2013). By comparison, sleep problems are generally viewed as more socially acceptable if not expected in this age group. The SHAWQ could therefore be used to flag students with sleep health problems who can be followed up for mental health problems.

As a research tool, the SHAWQ could be used as a relative indicator of sleep health in population studies or observational studies. In this context, the SHAWQ could be used to identify and understand the factors that either promote or constrain sleep health in students. Our study was motivated in part by the observation that sleep problems often precede depression (Gradisar et al., 2022). However, the present study only examined the cross-sectional associations of SHAWQ scores with depression symptoms. Longitudinal studies are needed to determine whether SHAWQ scores are predictive of mental health problems. The SHAWQ could also be used as an outcome variable to assess the effects of interventions that target sleep health or mental well-being.

### 4.7 Conclusions

The SHAWQ is a short questionnaire whose composite score and categories (good, fair, bad) can be used as relative indicators of sleep health in adolescents and university students in their teens and twenties. Higher SHAWQ scores were strongly associated with sleep problems, insomnia symptoms, and depression symptoms. Large effect sizes were observed for associations of bad sleep health with all sleep and mental health outcomes. Bad sleep health on the SHAWQ was also prospectively associated with lower grades. These findings suggest that the SHAWQ has the potential to screen for students who would benefit from interventions for sleep and mental health problems. The SHAWQ is also one of the shortest and easiest to score among sleep-health related instruments because there are no sub-scores, sleep-based calculations, or algorithms needed for its implementation.

## Data availability statement

Some datasets presented in this article are not readily available because of institutional and legal restrictions where the research was conducted. The raw data and code supporting the conclusions of Study 1 (first study in adolescents) and Study 3 (study in university students) will be made available by the authors without undue reservation. Permission to access data and code for Study 2 (second study in adolescents) should be directed to the National Institute of Education, Singapore (email contact: john.wang@nie.edu.sg). Raw data for Study 4 (university freshmen) includes university-archived data stored on the National University of Singapore (NUS) Institute for Applied Learning Sciences and Educational Technology (ALSET) Data Lake. In compliance with the Singapore Personal Data Protection Act, data on the ALSET Data Lake cannot be shared publically without student consent. Data and custom code used to analyse the data in Study 4 can be accessed on the ALSET Data Lake with approval by the NUS Learning and Analytics Committee on Ethics in accordance with NUS Data Management Policies. Researchers who wish to access the data and code should contact ALSET at NUS (email contact: alsbox1@nus.edu.sg). Requests to access the datasets should be directed to JG, joshua.gooley@duke-nus.edu.sg; JW, john.wang@nie.edu.sg; and ALSET, alsbox1@nus.edu.sg.

## Ethics statement

The studies involving humans were approved by the Institutional Review Board (IRB) at the National University of Singapore and the Nanyang Technological University. The studies were conducted in accordance with the local legislation and institutional requirements. The participants provided their written informed consent to participate in this study.

## Author contributions

YML and JG contributed to the design of the research, contributed to analyses, and wrote the article with input from all authors. PC, JW, and JG supervised the research. YML, SL, AVR, and JW collected and managed the data. All authors contributed to the article and approved the submitted version.

## References

[B1] Adelantado-Renau M. Beltran-Valls M. R. Migueles J. H. Artero E. G. Legaz-Arrese A. Capdevila-Seder A.. (2019). Associations between objectively measured and self-reported sleep with academic and cognitive performance in adolescents: DADOS study. J. Sleep Res. 28, e12811. 10.1111/jsr.12811 30609171

[B2] Alfonsi V. Scarpelli S. D'Atri A. Stella G. Gennaro D. (2020). Later school start time: the impact of sleep on academic performance and health in the adolescent population. Int. J. Environ. Res. Public Health. 17, 2574. 10.3390/ijerph17072574 32283688 PMC7177233

[B3] American Psychiatric Association (2013). Diagnostic and Statistical Manual of Mental Disorders: DSM-5. Washington, D. C.: American Psychiatric Association.

[B4] Appleton S. L. Melaku Y. A. Reynolds A. C. Gill T. K. de Batlle J. Adams R. J.. (2022). Multidimensional sleep health is associated with mental well-being in Australian adults. J. Sleep Res. 31, e13477. 10.1111/jsr.13477 34622511

[B5] Augustinavicius J. L. S. Zanjani A. Zakzanis K. K. Shapiro C. M. (2014). Polysomnographic features of early-onset depression: a meta-analysis. J. Affect. Disord. 158, 11–18. 10.1016/j.jad.2013.12.009 24655760

[B6] Baglioni C. Battagliese G. Feige B. Spiegelhalder K. Nissen C. Voderholzer U.. (2011). Insomnia as a predictor of depression: a meta-analytic evaluation of longitudinal epidemiological studies. J. Affect. Disord. 135, 10–19. 10.1016/j.jad.2011.01.011 21300408

[B7] Barham W. T. Buysse D. J. Kline C. E. Kubala A. G. Brindle R. C. (2022). Sleep health mediates the relationship between physical activity and depression symptoms. Sleep Breath. 26, 1341–1349. 10.1007/s11325-021-02496-9 34561758 PMC8475358

[B8] Basner M. Spaeth A. M. Dinges D. F. (2014). Sociodemographic characteristics and waking activities and their role in the timing and duration of sleep. Sleep 37, 1889–1906. 10.5665/sleep.4238 25325472 PMC4548514

[B9] Bastien C. H. Vallières A. Morin C. M. (2001). Validation of the Insomnia Severity Index as an outcome measure for insomnia research. Sleep Med. 2, 297–307. 10.1016/S1389-9457(00)00065-4 11438246

[B10] Bauducco S. Richardson C. Gradisar M. (2020). Chronotype, circadian rhythms and mood. Curr. Opini. 34, 77–83. 10.1016/j.copsyc.2019.09.002 31786494

[B11] Beebe D. W. Simon S. Summer S. Hemmer S. Strotman D. Dolan L. M.. (2013). Dietary intake following experimentally restricted sleep in adolescents. Sleep 36, 827–834. 10.5665/sleep.2704 23729925 PMC3649825

[B12] Benham G. (2019). The Sleep Health Index: correlations with standardized stress and sleep measures in a predominantly Hispanic college student population. Sleep Health 5, 587–591. 10.1016/j.sleh.2019.07.007 31422914

[B13] Blake M. J. Sheeber L. B. Youssef G. J. Raniti M. B. Allen N. B. (2017). Systematic review and meta-analysis of adolescent cognitive–behavioral sleep interventions. Clin. Child Fam. Psychol. Rev. 20, 227–249. 10.1007/s10567-017-0234-5 28331991

[B14] Bowers J. M. Moyer A. (2017). Effects of school start time on students' sleep duration, daytime sleepiness, and attendance: a meta-analysis. Sleep Health 3, 423–431. 10.1016/j.sleh.2017.08.004 29157635

[B15] Bowman M. A. Kline C. E. Buysse D. J. Kravitz H. M. Joffe H. Matthews K. A.. (2021). Longitudinal association between depressive symptoms and multidimensional sleep health: the SWAN sleep study. Ann. Behav. Med. 55, 641–652. 10.1093/abm/kaaa107 33410460 PMC8240133

[B16] Boz S. Lanquart J. P. Mungo A. Delhaye M. Loas G. Hein M. (2021). Risk of excessive daytime sleepiness associated to major depression in adolescents. Psychiat. Quart. 92, 1473–1488. 10.1007/s11126-021-09922-x 33956300

[B17] Brandolim Becker N. Martins R. I. S. Jesus S. N Chiodelli R. Stephen Rieber M. (2018). Sleep health assessment: a scale validation. Psychiatry Res. 259, 51–55. 10.1016/j.psychres.2017.10.014 29028524

[B18] Brooks S. J. Krulewicz S. P. Kutcher S. (2003). The Kutcher adolescent depression scale: assessment of its evaluative properties over the course of an 8-week pediatric pharmacotherapy trial. J. Child Adolesc. Psychopharmacol. 13, 337–349. 10.1089/104454603322572679 14642022

[B19] Buysse D. J. (2014). Sleep health: can we define it? does it matter? Sleep 37, 9–17. 10.5665/sleep.3298 24470692 PMC3902880

[B20] Buysse D. J. Germain A. Moul D. E. Franzen P. L. Brar L. K. Fletcher M. E.. (2011). Efficacy of brief behavioral treatment for chronic insomnia in older adults. Arch. Intern. Med. 171, 887–895. 10.1001/archinternmed.2010.535 21263078 PMC3101289

[B21] Buysse D. J. Reynolds C. F. Monk T. H. Berman S. R. Kupfer D. J. (1989). The Pittsburgh sleep quality index: a new instrument for psychiatric practice and research. Psychiatry Res. 28, 193–213. 10.1016/0165-1781(89)90047-4 2748771

[B22] Buysse D. J. Yu L. Moul D. E. Germain A. Stover A. Dodds N. E.. (2010). Development and validation of patient-reported outcome measures for sleep disturbance and sleep-related impairments. Sleep 33, 781–792. 10.1093/sleep/33.6.781 20550019 PMC2880437

[B23] Carrión-Pantoja S. Prados G. Chouchou F. Holguín M. Mendoza-Vinces Á. Expósito-Ruiz M.. (2022). Insomnia symptoms, sleep hygiene, mental health, and academic performance in spanish university students: a cross-sectional study. J. Clin. Med. 11, 1989. 10.3390/jcm11071989 35407597 PMC8999350

[B24] Carskadon M. A. (2011). Sleep in adolescents: the perfect storm. Pediatr. Clin. North Am. 58, 637–647. 10.1016/j.pcl.2011.03.003 21600346 PMC3130594

[B25] Carskadon M. A. Acebo C. Jenni O. G. (2004). Regulation of adolescent sleep: implications for behavior. Ann. N. Y. Acad. Sci. 1021, 276–291. 10.1196/annals.1308.032 15251897

[B26] Cella D. Yount S. Rothrock N. Gershon R. Cook K. Reeve B.. (2007). The patient-reported outcomes measurement information system (PROMIS): progress of an NIH roadmap cooperative group during its first two years. Med. Care. 45, S3. 10.1097/01.mlr.0000258615.42478.55 17443116 PMC2829758

[B27] Chen P. Y. Yang C. M. Morin C. M. (2015). Validating the cross-cultural factor structure and invariance property of the Insomnia Severity Index: evidence based on ordinal EFA and CFA. Sleep Med. 16, 598–603. 10.1016/j.sleep.2014.11.016 25863811

[B28] Cohen J. (1988). Statistical Power Analysis for the Behavioral Sciences (2nd ed.). New York, NY: Academic Press.

[B29] Cousins J. N. Fernández G. (2019). “Chapter 2 - The impact of sleep deprivation on declarative memory,” in Progress in Brain Research Sleep Deprivation and Cognition, eds. Van Dongen, H. P. A., Whitney, P., Hinson, J. M., Honn, K. A., and Chee, M. W. L. London: Elsevier, 27–53. 31072562 10.1016/bs.pbr.2019.01.007

[B30] Crook S. Sievi N. A. Bloch K. E. Stradling J. R. Frei A. Puhan M. A.. (2019). Minimum important difference of the Epworth sleepiness scale in obstructive sleep apnoea: estimation from three randomised controlled trials. Thorax 74, 390–396. 10.1136/thoraxjnl-2018-211959 30100576

[B31] Crowley S. J. Wolfson A. R. Tarokh L. Carskadon M. A. (2018). An update on adolescent sleep: New evidence informing the perfect storm model. J. Adolesc. 67, 55–65. 10.1016/j.adolescence.2018.06.001 29908393 PMC6054480

[B32] Dalmases M. Benítez I. D. Mas A. Garcia-Codina O. Medina-Bustos A. Escarrabill J.. (2015). Assessing sleep health in a European population: results of the Catalan Health Survey. PLoS ONE 13, e0194495. 10.1371/journal.pone.0194495 29668685 PMC5905963

[B33] DeSantis A. S. Dubowitz T. Ghosh-Dastidar B. Hunter G. P. Buman M. Buysse D. J.. (2019). A preliminary study of a composite sleep health score: associations with psychological distress, body mass index, and physical functioning in a low-income African American community. Sleep Health 5, 514–520. 10.1016/j.sleh.2019.05.001 31208939 PMC6801051

[B34] Dinis J. Bragança M. (2018). Quality of sleep and depression in college students: a systematic review. Sleep Sci. 11, 290–301. 10.5935/1984-0063.20180045 30746048 PMC6361309

[B35] Dong L. Martinez A. J. Buysse D. J. Harvey A. G. A. (2019). composite measure of sleep health predicts concurrent mental and physical health outcomes in adolescents prone to eveningness. Sleep Health 5, 166–174. 10.1016/j.sleh.2018.11.009 30928117 PMC6452900

[B36] Dong L. Xie Y. Zou X. (2022). Association between sleep duration and depression in US adults: a cross-sectional study. J. Affect. Disord. 296, 183–188. 10.1016/j.jad.2021.09.075 34607059

[B37] Dorrian J. Centofanti S. Smith A. McDermott K. D. (2019). “Chapter 4 - Self-regulation and social behavior during sleep deprivation,” in Progress in Brain Research, Sleep Deprivation, and Cognition eds. Van Dongen, H. P. A., Whitney, P., Hinson, J. M., Honn, K. A., and Chee, M. W. L. London: Elsevier, 73–110. 31072564 10.1016/bs.pbr.2019.03.010

[B38] Drake C. Nickel C. Burduvali E. Roth T. Jefferson C. Pietro B.. (2003). The pediatric daytime sleepiness scale (PDSS): sleep habits and school outcomes in middle-school children. Sleep 26, 455–458. 10.1037/t02761-000 12841372

[B39] Dunn V. Goodyer I. M. (2006). Longitudinal investigation into childhood-and adolescence-onset depression: psychiatric outcome in early adulthood. Br. J. Psychiatry. 188, 216–222. 10.1192/bjp.188.3.216 16507961

[B40] Eaton W. W. Smith C. Ybarra M. Muntaner C. Tien A. (2004). “Center for Epidemiologic Studies Depression Scale: Review and Revision (CESD and CESD-R),” in The Use of Psychological Testing for Treatment Planning and Outcomes Assessment: Instruments for Adults. Mahwah, NJ, US: Lawrence Erlbaum Associates Publishers, 363–77.

[B41] Faro A. Eaton W. W. A. (2020). Diagnostic-oriented screening scale for anxiety disorders: the center for epidemiologic studies anxiety scale (CESA). Front. Psychol. 11, 957. 10.3389/fpsyg.2020.00957 32528370 PMC7265241

[B42] Fergusson D. M. Boden J. M. Horwood L. J. (2007). Recurrence of major depression in adolescence and early adulthood, and later mental health, educational and economic outcomes. Br. J. Psychiatry. 191, 335–342. 10.1192/bjp.bp.107.036079 17906244

[B43] Forrest C. B. Meltzer L. J. Marcus C. L. (2018). de la Motte A, Kratchman A, Buysse DJ, et al. Development and validation of the PROMIS Pediatric Sleep Disturbance and Sleep-Related Impairment item banks. Sleep 41, zsy054. 10.1093/sleep/zsy054 29546286

[B44] Fredriksen K. Rhodes J. Reddy R. Way N. (2004). Sleepless in Chicago: tracking the effects of adolescent sleep loss during the middle school years. Child Dev. 75, 84–95. 10.1111/j.1467-8624.2004.00655.x 15015676

[B45] Furihata R. Hall M. H. Stone K. L. Ancoli-Israel S. Smagula S. F. Cauley J. A. (2017). An aggregate measure of sleep health is associated with prevalent, incident clinically significant depression symptoms among community-dwelling older women. Sleep 40, zsw075. 10.1093/sleep/zsw075 28364417 PMC6084764

[B46] Gee B. Orchard F. Clarke E. Joy A. Clarke T. Reynolds S.. (2019). The effect of non-pharmacological sleep interventions on depression symptoms: a meta-analysis of randomised controlled trials. Sleep Med. Rev. 43, 118–128. 10.1016/j.smrv.2018.09.004 30579141

[B47] Goh C. M. J. Shahwan S. Lau J. H. Ong W. J. Tan G. T. H. Samari E.. (2021). Advancing research to eliminate mental illness stigma: an interventional study to improve community attitudes towards depression among University students in Singapore. BMC Psychiatry. 21, 108. 10.1186/s12888-021-03106-4 33602155 PMC7890908

[B48] Gomes A. A. Tavares J. Azevedo D. (2011). Sleep and academic performance in undergraduates: a multi-measure, multi-predictor approach. Chronobiol. Int. 28, 786–801. 10.3109/07420528.2011.606518 22080785

[B49] Gonsalvez I. Li J. J. Stevens C. Chen J. A. Liu C. H. (2022). Preexisting depression and daytime sleepiness in women and men. Behav. Sleep Med. 20, 380–392. 10.1080/15402002.2021.1924720 34003712 PMC8599528

[B50] Gordon A. M. Carrillo B. Barnes C. M. (2021). Sleep and social relationships in healthy populations: a systematic review. Sleep Med. Rev. 57, 101428. 10.1016/j.smrv.2021.101428 33596514

[B51] Gordon A. M. Mendes W. B. Prather A. A. (2017). The social side of sleep: Elucidating the links between sleep and social processes. Curr. Dir. Psychol. Sci. 26, 470–475. 10.1177/0963721417712269 29398789 PMC5791747

[B52] Gradisar M. Gardner G. Dohnt H. (2011). Recent worldwide sleep patterns and problems during adolescence: a review and meta-analysis of age, region, and sleep. Sleep Med. 12, 110–118. 10.1016/j.sleep.2010.11.008 21257344

[B53] Gradisar M. Kahn M. Micic G. Short M. Reynolds C. Orchard F.. (2022). Sleep's role in the development and resolution of adolescent depression. Nat. Rev. 1, 512–523. 10.1038/s44159-022-00074-8 35754789 PMC9208261

[B54] Hale L. Troxel W. Buysse D. J. (2020). Sleep health: an opportunity for public health to address health equity. Annu. Rev. Public Health. 41, 81–99. 10.1146/annurev-publhealth-040119-094412 31900098 PMC7944938

[B55] Hartmann M. E. Prichard J. R. (2018). Calculating the contribution of sleep problems to undergraduates' academic success. Sleep Health 4, 463–471. 10.1016/j.sleh.2018.07.002 30241662

[B56] Hattie J. (2015). The applicability of visible learning to higher education. Scholar. Teach. Learn. Psychol. 1, 79–91. 10.1037/stl0000021

[B57] Ho J. Tumkaya T. Aryal S. Choi H. Claridge-Chang A. (2019). Moving beyond P values: data analysis with estimation graphics. Nat. Methods. 16, 565–566. 10.1038/s41592-019-0470-3 31217592

[B58] Huang Y. Zhu M. (2020). Increased global PSQI score is associated with depressive symptoms in an adult population from the United States. Nat. Sci. Sleep. 12, 487–495. 10.2147/NSS.S256625 32765145 PMC7381800

[B59] Hyde J. S. Mezulis A. H. Abramson L. Y. (2008). The ABCs of depression: integrating affective, biological, and cognitive models to explain the emergence of the gender difference in depression. Psychol. Rev. 115, 291–313. 10.1037/0033-295X.115.2.291 18426291

[B60] Hysing M. Pallesen S. Stormark K. M. Lundervold A. J. Sivertsen B. (2013). Sleep patterns and insomnia among adolescents: a population-based study. J. Sleep Res. 22, 549–556. 10.1111/jsr.12055 23611716

[B61] Jenni O. G. Achermann P. Carskadon M. A. (2005). Homeostatic sleep regulation in adolescents. Sleep 28, 1446–1454. 10.1093/sleep/28.11.1446 16335485

[B62] Ji X. Liu J. (2016). Subjective sleep measures for adolescents: a systematic review. Child Care Health Dev. 42, 825–839. 10.1111/cch.12376 27495828

[B63] Johns M. W. A. (1991). New method for measuring daytime sleepiness: the epworth sleepiness scale. Sleep. 14, 540–545. 10.1093/sleep/14.6.540 1798888

[B64] Johnson E. O. Roth T. Breslau N. (2006). The association of insomnia with anxiety disorders and depression: exploration of the direction of risk. J. Psychiatr. Res. 40, 700–708. 10.1016/j.jpsychires.2006.07.008 16978649

[B65] Kalin N. H. (2020). The critical relationship between anxiety and depression. Am. J. Psychiatry. 177, 365–367. 10.1176/appi.ajp.2020.20030305 32354270

[B66] Knutson K. L. Phelan J. Paskow M. J. Roach A. Whiton K. Langer G.. (2017). The national sleep foundation's sleep health index. Sleep Health. 3, 234–240. 10.1016/j.sleh.2017.05.011 28709508

[B67] Koo T. K. Li M. Y. (2016). A guideline of selecting and reporting intraclass correlation coefficients for reliability research. J. Chiropr. Med. 15, 155–163. 10.1016/j.jcm.2016.02.012 27330520 PMC4913118

[B68] LeBlanc J. C. Almudevar A. Brooks S. J. Kutcher S. (2002). Screening for adolescent depression: comparison of the kutcher adolescent depression scale with the beck depression inventory. J. Child Adolesc. Psychopharmacol. 12, 113–26. 10.1089/104454602760219153 12188980

[B69] LeBourgeois M. K. Giannotti F. Cortesi F. Wolfson A. R. Harsh J. (2005). The relationship between reported sleep quality and sleep hygiene in Italian and American adolescents. Pediatrics 115, 257–65. 10.1542/peds.2004-0815H 15866860 PMC3928632

[B70] Lee S. Lawson K. M. (2021). Beyond single sleep measures: a composite measure of sleep health and its associations with psychological and physical well-being in adulthood. Soc. Sci. Med. 274, 113800. 10.1016/j.socscimed.2021.113800 33652324 PMC12666767

[B71] Lemma S. Berhane Y. Worku A. Gelaye B. Williams M. A. (2014). Good quality sleep is associated with better academic performance among university students in Ethiopia. Sleep Breath. 18, 257–263. 10.1007/s11325-013-0874-8 23928956 PMC3918486

[B72] Lewandowski A. S. Toliver-Sokol M. Palermo T. M. (2011). Evidence-based review of subjective pediatric sleep measures. J. Pediatr. Psychol. 36, 780–793. 10.1093/jpepsy/jsq119 21227912 PMC3146754

[B73] Lin Y. Tremblay M. S. Katzmarzyk P. T. Fogelholm M. Hu G. Lambert E. V.. (2018). Temporal and bi-directional associations between sleep duration and physical activity/sedentary time in children: an international comparison. Prev. Med. 111, 436–441. 10.1016/j.ypmed.2017.12.006 29223790 PMC9048858

[B74] Lo J. C. Ong J. L. Leong R. L. F. Gooley J. J. Chee M. W. L. (2016). Cognitive performance, sleepiness, and mood in partially sleep deprived adolescents: the need for sleep study. Sleep 39, 687–698. 10.5665/sleep.5552 26612392 PMC4763363

[B75] Lovato N. Gradisar M. A. (2014). Meta-analysis and model of the relationship between sleep and depression in adolescents: recommendations for future research and clinical practice. Sleep Med. Rev. 18, 521–529. 10.1016/j.smrv.2014.03.006 24857255

[B76] Luo C. Zhang J. Pan J. (2013). One-Year Course and Effects of Insomnia in Rural Chinese Adolescents. Sleep 36, 377–384. 10.5665/sleep.2454 23450433 PMC3571740

[B77] Manzar M. D. BaHammam A. S. Hameed U. A. Spence D. W. Pandi-Perumal S. R. Moscovitch A.. (2018). Dimensionality of the Pittsburgh Sleep Quality Index: a systematic review. Health Qual. Life Outcomes. 16, 89. 10.1186/s12955-018-0915-x PMC5944037 29743066

[B78] Manzar M. D. Jahrami H. A. Bahammam A. S. (2021). Structural validity of the Insomnia Severity Index: a systematic review and meta-analysis. Sleep Med. Rev. 60, 101531. 10.1016/j.smrv.2021.101531 34428679

[B79] Marino C. Andrade B. Campisi S. C. Wong M. Zhao H. Jing X.. (2021). Association between disturbed sleep and depression in children and youths: a systematic review and meta-analysis of cohort studies. JAMA Network Open. 4, e212373. 10.1001/jamanetworkopen.2021.2373 33749768 PMC7985724

[B80] Marx R. Tanner-Smith E. E. Davison C. M. Ufholz L. A. Freeman J. Shankar R.. (2017). Later school start times for supporting the education, health, and well-being of high school students. Cochrane Database Syst. Rev. 7, CD009467. 10.1002/14651858.CD009467.pub2 PMC6483483 28670711

[B81] Massar S. A. A. Lim J. Huettel S. A. (2019). “Chapter 1 - Sleep deprivation, effort allocation and performance,” in Progress in Brain Research, eds. Van Dongen, H. P. A., Whitney, P., Hinson, J. M., Honn, K. A., and Chee, M. W. L. London: Elsevier, 1–26. 31072557 10.1016/bs.pbr.2019.03.007

[B82] Meijer A. M. (2008). Chronic sleep reduction, functioning at school and school achievement in preadolescents. J. Sleep Res. 17, 395–405. 10.1111/j.1365-2869.2008.00677.x 19021856

[B83] Meltzer L. J. Saletin J. M. Honaker S. M. Owens J. A. Seixas A. Wahlstrom K. L.. (2021). COVID-19 instructional approaches (in-person, online, hybrid), school start times, and sleep in over 5,000 U.S. adolescents. Sleep 44, zsab180. 10.1093/sleep/zsab180 34401922 PMC8385997

[B84] Minges K. E. Redeker N. S. (2016). Delayed school start times and adolescent sleep: a systematic review of the experimental evidence. Sleep Med. Rev. 28, 86–95. 10.1016/j.smrv.2015.06.002 26545246 PMC4844764

[B85] Netzer N. C. Stoohs R. A. Netzer C. M. Clark K. Strohl K. P. (1999). Using the berlin questionnaire to identify patients at risk for the sleep apnea syndrome. Ann. Intern. Med. 131, 485–491. 10.7326/0003-4819-131-7-199910050-00002 10507956

[B86] O'Callaghan V. S. Couvy-Duchesne B. Strike L. T. McMahon K. L. Byrne E. M. Wright M. J. A.. (2021). meta-analysis of the relationship between subjective sleep and depressive symptoms in adolescence. Sleep Med. 79, 134–144. 10.1016/j.sleep.2021.01.011 33524839

[B87] Olds T. Blunden S. Petkov J. Forchino F. (2010). The relationships between sex, age, geography and time in bed in adolescents: a meta-analysis of data from 23 countries. Sleep Med. Rev. 14, 371–378. 10.1016/j.smrv.2009.12.002 20207558

[B88] Ong J. L. Tandi J. Patanaik A. Lo J. C. Chee M. W. L. (2019). Large-scale data from wearables reveal regional disparities in sleep patterns that persist across age and sex. Sci. Rep. 9, 3415. 10.1038/s41598-019-40156-x 30833649 PMC6399225

[B89] Orchard F. Gregory A. M. Gradisar M. Reynolds S. (2020). Self-reported sleep patterns and quality amongst adolescents: cross-sectional and prospective associations with anxiety and depression. J. Child Psychol. Psychiatry. 61, 1126–1137. 10.1111/jcpp.13288 32557672

[B90] Orchard F. Reynolds S. (2018). The combined influence of cognitions in adolescent depression: Biases of interpretation, self-evaluation, and memory. Br. J. Clini. Psychol. 57, 420–435. 10.1111/bjc.12184 29799126 PMC6175080

[B91] Orzech K. M. Salafsky D. B. Hamilton L. A. (2011). The state of sleep among college students at a large public university. J. Am. College Health 59, 612–619. 10.1080/07448481.2010.520051 21823956

[B92] Pang S. Liu J. Mahesh M. Chua B. Y. Shahwan S. Lee S. P.. (2017). Stigma among Singaporean youth: a cross-sectional study on adolescent attitudes towards serious mental illness and social tolerance in a multiethnic population. BMJ Open. 7, e016432. 10.1136/bmjopen-2017-016432 29042379 PMC5652546

[B93] Patel S. Kon S. S. C. Nolan C. M. Barker R. E. Simonds A. K. Morrell M. J.. (2018). The Epworth sleepiness scale: minimum clinically important difference in obstructive sleep apnea. Am. J. Respir. Crit. Care Med. 197, 961–963. 10.1164/rccm.201704-0672LE 28961021 PMC6020404

[B94] R Core Team (2022). R: A Language and Environment for Statistical Computing. Vienna, Austria: R Foundation for Statistical Computing.

[B95] Raniti M. B. Allen N. B. Schwartz O. Waloszek J. M. Byrne M. L. Woods M. J.. (2017). Sleep duration and sleep quality: associations with depressive symptoms across adolescence. Behav. Sleep Med. 15, 198–215. 10.1080/15402002.2015.1120198 26744783

[B96] Raniti M. B. Waloszek J. M. Schwartz O. Allen N. B. Trinder J. (2018). Factor structure and psychometric properties of the Pittsburgh Sleep Quality Index in community-based adolescents. Sleep 41, zsy066. 10.1093/sleep/zsy066 29608755

[B97] Ravyts S. G. Dzierzewski J. M. Perez E. Donovan E. K. Dautovich N. D. (2021). Sleep health as measured by RU SATED: a psychometric evaluation. Behav. Sleep Med. 19, 48–56. 10.1080/15402002.2019.1701474 31829724 PMC7289662

[B98] Richardson C. Gradisar M. (2022). Depressed mood and repetitive negative thinking in delayed sleep–wake phase disorder: treatment effects and a comparison with good sleepers. J. Sleep Res. 31, e13452. 10.1111/jsr.13452 34350657

[B99] Richter S. A. Ferraz-Rodrigues C. Schilling L. B. Camargo N. F. Nunes M. L. (2023). Effects of the COVID-19 pandemic on sleep quality in children and adolescents: A systematic review and meta-analysis. J. Sleep Res. 32:e13720. 10.1111/jsr.13720 36000251 PMC9539085

[B100] Roane B. M. Taylor D. J. (2008). Adolescent insomnia as a risk factor for early adult depression and substance abuse. Sleep 31, 1351–1356. 10.5665/sleep/31.10.1351 18853932 PMC2572740

[B101] Roberts R. E. Duong H. T. (2014). The Prospective Association between Sleep Deprivation and Depression among Adolescents. Sleep 37, 239–244. 10.5665/sleep.3388 24497652 PMC3900610

[B102] Roenneberg T. Wirz-Justice A. Merrow M. (2003). Life between clocks: daily temporal patterns of human chronotypes. J. Biol. Rhythms. 18, 80–90. 10.1177/0748730402239679 12568247

[B103] Rudolph K. D. Klein D. N. (2009). Exploring depressive personality traits in youth: Origins, correlates, and developmental consequences. Dev. Psychopathol. 21, 1155–1180. 10.1017/S0954579409990095 19825262 PMC2762644

[B104] Schäfer T. Schwarz M. A. (2019). The meaningfulness of effect sizes in psychological research: differences between sub-disciplines and the impact of potential biases. Front. Psychology 10, 813. 10.3389/fpsyg.2019.00813 31031679 PMC6470248

[B105] Sheskin D. (2000). Handbook of Parametric and Nonparametric Statistical Procedures, 2nd ed. Boca Raton: Chapman and Hall/CRC.

[B106] Shimamoto H. Suwa M. Mizuno K. (2021). Relationships between depression, daily physical activity, physical fitness, and daytime sleepiness among Japanese University students. Int. J. Environ. Res. Public Health. 18, 8036. 10.3390/ijerph18158036 34360329 PMC8345676

[B107] Short M. A. Booth S. A. Omar O. Ostlundh L. Arora T. (2020). The relationship between sleep duration and mood in adolescents: a systematic review and meta-analysis. Sleep Med. Rev. 52, 101311. 10.1016/j.smrv.2020.101311 32240932

[B108] Short M. A. Chee M. W. L. (2019). Adolescent sleep restriction effects on cognition and mood. Prog. Brain Res. 246, 55–71. 10.1016/bs.pbr.2019.02.008 31072563

[B109] Short M. A. Gradisar M. Lack L. C. Wright H. R. (2013). The impact of sleep on adolescent depressed mood, alertness and academic performance. J. Adolesc. 36, 1025–1033. 10.1016/j.adolescence.2013.08.007 24215949

[B110] Short M. A. Gradisar M. Wright H. Lack L. C. Dohnt H. Carskadon M. A.. (2011). Time for bed: parent-set bedtimes associated with improved sleep and daytime functioning in adolescents. Sleep 34, 797–800. 10.5665/SLEEP.1052 21629368 PMC3098947

[B111] Stea T. H. Knutsen T. Torstveit M. K. (2014). Association between short time in bed, health-risk behaviors and poor academic achievement among Norwegian adolescents. Sleep Med. 15, 666–671. 10.1016/j.sleep.2014.01.019 24767727

[B112] Steptoe A. Peacey V. Wardle J. (2006). Sleep duration and health in young adults. Arch. Intern. Med. 166, 1689–1692. 10.1001/archinte.166.16.1689 16983045

[B113] Taylor D. J. Jenni O. G. Acebo C. Carskadon M. A. (2005). Sleep tendency during extended wakefulness: insights into adolescent sleep regulation and behavior. J. Sleep Res. 14, 239–244. 10.1111/j.1365-2869.2005.00467.x 16120098

[B114] Thapar A. Collishaw S. Pine D. S. Thapar A. K. (2012). Depression in adolescence. Lancet 379, 1056–1067. 10.1016/S0140-6736(11)60871-4 22305766 PMC3488279

[B115] Tomaso C. C. Johnson A. B. Nelson T. D. (2021). The effect of sleep deprivation and restriction on mood, emotion, and emotion regulation: three meta-analyses in one. Sleep 44, zsaa289. 10.1093/sleep/zsaa289 33367799 PMC8193556

[B116] Tsou M. T. Chang B. C. (2019). Association of depression and excessive daytime sleepiness among sleep-deprived college freshmen in Northern Taiwan. Int. J. Environ. Res. Public Health. 16, 3148. 10.3390/ijerph16173148 31470500 PMC6747465

[B117] Vaingankar J. A. Rekhi G. Subramaniam M. Abdin E. Chong S. A. (2013). Age of onset of life-time mental disorders and treatment contact. Soc. Psychiatry Psychiatr. Epidemiol. 48, 835–843. 10.1007/s00127-012-0601-y 23076588

[B118] Van Dyk T. R. Krietsch K. N. Saelens B. E. Whitacre C. McAlister S. Beebe D. W.. (2018). Inducing more sleep on school nights reduces sedentary behavior without affecting physical activity in short-sleeping adolescents. Sleep Med. 47, 7–10. 10.1016/j.sleep.2018.03.007 29880148

[B119] van Kooten J. Terwee C. B. Luijten M. A. J. Steur L. M. H. Pillen S. Wolters N. G. J.. (2021). psychometric properties of the patient-reported outcomes measurement information system (PROMIS) sleep disturbance and sleep-related impairment item banks in adolescents. J. Sleep Res. 30, e13029. 10.1111/jsr.13029 32180280 PMC8047882

[B120] Van Meter A. R. Anderson E. A. (2020). Evidence base update on assessing sleep in youth. J. Clin. Child Adolesc. Psychol. 49, 701–736. 10.1080/15374416.2020.1802735 33147074

[B121] Vargha A. Delaney H. D. A. (2000). Critique and improvement of the CL common language effect size statistics of McGraw and Wong. J. Educ. Behavioral Stat. 25, 101–132. 10.3102/10769986025002101

[B122] Vgontzas A. N. Liao D. Bixler E. O. Chrousos G. P. Vela-Bueno A. (2009). Insomnia with objective short sleep duration is associated with a high risk for hypertension. Sleep 32, 491–497. 10.1093/sleep/32.4.491 19413143 PMC2663863

[B123] What Works Clearinghouse (2020). What Works Clearinghouse™ *Procedures Handbook, Version 4.1*. Washington, DC: U.S. Department of Education, Institute Of Education Sciences, National Center for Education Evaluation and Regional Assistance.

[B124] Wheaton A. G. Chapman D. P. Croft J. B. (2016). School start times, sleep, behavioral, health, and academic outcomes: a review of the literature. J. School Health 86, 363–381. 10.1111/josh.12388 27040474 PMC4824552

[B125] Whiteford H. A. Degenhardt L. Rehm J. Baxter A. J. Ferrari A. J. Erskine H. E.. (2013). Global burden of disease attributable to mental and substance use disorders: findings from the Global Burden of Disease Study (2010). Lancet 382, 1575–1586. 10.1016/S0140-6736(13)61611-6 23993280

[B126] Wolfson A. R. Carskadon M. A. (1998). Sleep schedules and daytime functioning in adolescents. Child Dev. 69, 875–887. 10.1111/j.1467-8624.1998.tb06149.x 9768476

[B127] World Health Organization (2004). The World Health Organization quality of life (WHOQOL) - BREF. Geneva: World Health Organization.

[B128] Yang M. Morin C. M. Schaefer K. Wallenstein G. V. (2009). Interpreting score differences in the Insomnia Severity Index: using health-related outcomes to define the minimally important difference. Curr. Med. Res. Opin. 25, 2487–2494. 10.1185/03007990903167415 19689221

[B129] Yeo S. C. Jos A. M. Erwin C. Lee S. M. Lee X. K. Lo J. C.. (2019). Associations of sleep duration on school nights with self-rated health, overweight, and depression symptoms in adolescents: problems and possible solutions. Sleep Med. 60, 96–108. 10.1016/j.sleep.2018.10.041 30611714

[B130] Yeo S. C. Lai C. K. Y. Tan J. Lim S. Chandramoghan Y. Tan T. K.. (2023). Early morning university classes are associated with impaired sleep and academic performance. Nat. Hum. Behav. 7, 502–514. 10.1038/s41562-023-01531-x 36806401 PMC10129866

[B131] Yeo S. C. Tan J. Lai C. K. Y. Lim S. Chandramoghan Y. Fung F. M.. (2021). University-wide chronotyping shows late-type students have lower grades, shorter sleep, poorer well-being, lower self-regulation, and more absenteeism. bioRxiv. 10.1101/2021.08.04.455177

[B132] Yeo S. C. Tan J. Lo J. C. Chee M. W. L. Gooley J. J. (2020). Associations of time spent on homework or studying with nocturnal sleep behavior and depression symptoms in adolescents from Singapore. Sleep Health (2020). 6, 758–766. 10.1016/j.sleh.2020.04.011 32536472

[B133] Zhai L. Zhang H. Zhang D. (2015). Sleep duration and depression among adults: a meta-analysis of prospective studies. Depress. Anxiety. 32, 664–670. 10.1002/da.22386 26047492

[B134] Zhang L. G. Cheng L. F. Wang T. T. Wang L. L. Luo Y. H.. (2023). Chain mediating effect of insomnia, depression, and anxiety on the relationship between nightmares and cognitive deficits in adolescents. J. Affect. Disord. 322, 2–8. 10.1016/j.jad.2022.10.047 36343783

