# Supplementary material for: Development and testing of the Sleep Health And Wellness Questionnaire (SHAWQ) in adolescents and university students: composite SHAWQ scores are associated with sleep problems, depression symptoms, and academic performance
Source: Front Sleep. 2023 Nov 15;2:1188424. doi: 10.3389/frsle.2023.1188424 (PMC12713909; doi:10.3389/frsle.2023.1188424)
Supplement: Supplementary file 1 [file Data_Sheet_1.docx]

**SUPPLEMENTARY MATERIALS**

**Title:** Development and testing of the Sleep Health And Wellness Questionnaire (SHAWQ) in adolescents and university students: Composite SHAWQ scores are associated with sleep problems, depression symptoms, and academic performance

**Authors:** Yng Miin Loke^1^, Samantha Lim^1^, A.V. Rukmini^1^, Patricia Chen^2,3^, John C.K. Wang^4^, Joshua J. Gooley^1,2*^

^1^Neuroscience and Behavioural Disorders Programme, Duke-NUS Medical School, Singapore

^2^Institute for Applied Learning Sciences and Educational Technology, National University of Singapore, Singapore

^3^Department of Educational Psychology, The University of Texas at Austin, Austin, TX, USA

^4^Physical Education & Sports Science, National Institute of Education, Singapore

*** Correspondence:**Joshua J. Gooley
joshua.gooley@duke-nus.edu.sg

**Keywords:** sleep; health; depression; anxiety; grades; academic performance

**1. SUPPLEMENTARY METHODS**

**1.1 Origin of the SHAWQ questions**

The goal of our study was to develop a short instrument (i.e., up to several multiple-choice questions) that can be used to generate a sleep health score in adolescents that is predictive of depression symptoms. Our strategy was to select for combinations of sleep variables in our sleep habits survey that were associated most strongly with depression symptoms. First, we shortlisted 22 questions that we expected to be linked to adolescents’ sleep health and/or well-being (**Fig. 1a**). These questions included information about sleep behaviour on school days and non-school days (bedtime, wake-up time, nocturnal total sleep time, frequency of napping), sleep quality and continuity (self-rated sleep quality, sleep latency, nocturnal awakenings, waking up earlier than desired), frequency of staying awake until 3:00 am or later, daytime sleepiness, and taking caffeine to stay awake. We also included questions related to demographic factors (age, sex), self-rated health, and media use because these factors have been shown to associate with sleep and mental health outcomes (1-8). We excluded questions that were unlikely to be generalizable to other cultural settings (e.g., ethnicity, type of school, grades, time-use). We did not consider sleep variables calculated from two or more other sleep variables (e.g., midpoint of nocturnal sleep, or differences in sleep parameters between school days and non-school days).

The SHAWQ was based on the best 6-predictor model from the best-subsets regression model. Most of the selected questions originated from instruments that are widely used in sleep research. The question on sleep quality was based on the Pittsburgh Sleep Quality Index (PSQI) but was modified to cover sleep in the past 2 weeks rather than a month (9). We also added a response option (“Okay”) so that there were a total of 5 modified response options. (“Very Good”, “Good”, “Okay”, “Bad”, and “Very Bad”). The questions on daytime sleepiness, staying up late, sleep onset latency, and self-rated health were taken from the School Sleep Habits Survey with minor modifications (10). Among the questions included in the 6-predictor model, the sleep latency question was the only one with a free response. Given that we aimed to develop a multiple choice questionnaire, we binned participants’ free responses for sleep latency into different durations (<15 min, 15 to <30 min, 30 to 60 min, and >60 min) prior to developing our SHAWQ scoring method.

**1.2 Definitions of SHAWQ categories**

We sought to categorize students into good, fair, and bad sleep health groups based on their SHAWQ score and its association with global depression score. The rationale was to establish threshold scores that can be used to screen students with poor sleep health who may be at risk of mental health problems. To assess potential cut-off values for defining SHAWQ categories, we examined the distribution of SHAWQ scores (**Supplementary Figure 1**), and we plotted the mean KADS depression score against adolescents’ SHAWQ score (**Fig. 3b**). SHAWQ scores ≤ 7 fell below the mode of the distribution, and based on visual inspection there was a slightly greater increase (i.e., a small ‘jump’ with no overlap in the 95% CIs) in the average depression score when SHAWQ scores increased from 7 to 8. There was also a small drop in the frequency of SHAWQ scores beyond a value of 16, and the average depression score for a SHAWQ score of 16 was about twice as high compared with a SHAWQ score of 7. We therefore preliminarily set these values (SHAWQ scores >7 and >16) as the threshold transitions from good to fair sleep health, and from fair to bad sleep health. Applying these thresholds, the distribution of depression scores differed substantially across good, fair, and bad SHAWQ categories with large effect sizes (**Fig. 3c**). Relative to students with good sleep health, the mean depression score on the KADS was about 1 standard deviation higher in students with fair sleep health (mean difference in KADS score=3.81, 95% CI=3.42 to 4.20; Cohen’s d=0.92, 95% CI=0.82 to 1.02, *p*<0.001), and more than 2 standard deviations higher in students with bad sleep health (mean difference in KADS score=9.69, 95% CI=8.70 to 10.81; Cohen’s d=2.32, 95% CI=2.06 to 2.58, *p*<0.001) (**Fig. 4a; Supplementary Table 2**). Based on these results, we defined good sleep health as SHAWQ scores ranging from 3 to 7, fair sleep health as SHAWQ scores ranging from 8 to 16, and bad sleep health as SHAWQ scores ranging from 17 to 35.

**1.3 Survey instruments**

*Kutcher Adolescent Depression Scale (KADS)*.

The 11-item KADS included descriptions related to sadness, irritability, sleep difficulties, apathy, feelings of worthlessness, fatigue/low motivation, lack of focus, anhedonia, anxiety, physical signs of anxiety, and thoughts of self-harm or suicide (11, 12). Adolescents were instructed to choose among the following options describing the frequency of symptoms over the past week: “Hardly ever”, “Some of the time”, “Most of the time”, and “All of the time”. Each response was assigned a score of 0 to 3, and the global depression score was determined by summing the scores across items (range=0 to 33). The KADS-11 has psychometric properties similar to other self-report instruments used to evaluate adolescent depression, with good internal consistency (Cronbach’s α, range=0.80 to 0.87) (11, 13), test-retest reliability (Pearson’s *r*=0.77) (13), and concurrent validity with the Children’s Depression Inventory (Pearson’s *r*=0.74) (13). There is no established cut-off score for diagnosing depression on the KADS. We therefore used the KADS score as a relative indicator of depression severity and performed item-by-item analyses of individual depression symptoms.

*Munich Chronotype Questionnaire (MCTQ)*

The MCTQ was used to assess adolescents’ bedtime and wake-up time on school days and non-school days (i.e., weekends or free days) (14). The MCTQ asked students to report their usual sleep behaviour over the past 4 weeks, and the questionnaire included a series of pictures to help depict the meaning of each response item. We defined bedtime as when students first attempted sleep (e.g., lights out), which is not necessarily the same as when they got into bed. Bedtime was assessed with the statement “I actually get ready to fall asleep at _____ o’clock”, and wake-up time was assessed with the statement “I wake up at _____o’clock”. The midpoint of the attempted sleep period on free days was used as a relative indicator of students’ chronotype (i.e., later midpoint of sleep indicates a later chronotype).

*Epworth Sleepiness Scale (ESS)*

The ESS was used to evaluate excessive daytime sleepiness (15). The ESS included 8 items in which participants were asked to rate the likelihood of dozing off or falling asleep while engaged in everyday scenarios: “sitting and reading”, “watching TV”, “sitting inactive in a public place (e.g., a theatre or a meeting)”, “as a passenger in a vehicle for an hour without a break”, “lying down to rest in the afternoon when circumstances permit”, “sitting and talking to someone”, “sitting quietly after a lunch without alcohol”, and “in a vehicle, while stopped for a few minutes in traffic”. Participants responded using a 4-point scale with the options “would never doze” (0), “slight chance of dozing” (1), “moderate chance of dozing” (2), and “high chance of dozing” (3). The ESS score was calculated as the sum of scores across the 8 items (range=0 to 24), with a score >10 interpreted as evidence of excessive daytime sleepiness (ESS score: 11-12, mild; 13-15, moderate; 16-24, severe). The ESS has good internal consistency (Cronbach’s α, range=0.73 to 0.90) (16-20), and good test-retest reliability (intraclass correlation coefficient, range=0.78 to 0.93) (16, 18, 21, 22).

*Pittsburgh Sleep Quality Index (PSQI)*

The PSQI was used to assess sleep quality over the past month (9). The PSQI comprised 9 questions (requiring 18 different responses) used to derive integer component scores (range=0 to 3) for 7 dimensions/factors related to sleep health: sleep quality, sleep latency, sleep duration, sleep efficiency, sleep disturbances, use of sleep medications, and daytime dysfunction. The component scores were summed to generate a global PSQI score (range=0 to 21). A PSQI score >5 was interpreted as evidence of poor sleep quality. The PSQI has good internal consistency (Cronbach’s α, range=0.64 to 0.83) and good test-retest reliability (intraclass correlation coefficient, range=0.70 to 0.86) (23).

*Insomnia Severity Index (ISI)*

The ISI was used to assess symptoms associated with insomnia (24). The ISI comprised 5 questions (requiring 7 different responses) that covered sleep efficiency/continuity (difficulty falling asleep, difficulty staying asleep, or waking up too early), satisfaction with sleep, daytime functioning, how noticeable the sleep problem is to others, and level of worry/ distress about the sleep problem. Each item was rated on a 5-point scale (ranging from 0 to 4) and the item scores were summed to generate a global insomnia score ranging from 0 to 28. An ISI score >14 is usually interpreted as evidence of clinical insomnia (ISI score: 0-7, no clinically significant insomnia; 8-14, subthreshold insomnia; 15-21, clinical insomnia, moderate severity; 22-28, clinical insomnia, severe). The ISI has good internal consistency (Cronbach’s α, range=0.70 to 0.91) (24-26) and good test-retest reliability (intraclass correlation coefficient, range=0.76 to 0.91) (26).

*Center for Epidemiologic Studies Depression Scale Revised (CESDR)*

The CESDR is a 20-item scale that assesses depression symptoms across 9 dimensions including sadness/dysphoria (e.g., feeling sad, depressed, the blues), anhedonia (e.g., nothing makes me happy, losing interest in things), fatigue (e.g., tired all the time, cannot get going), thinking/concentration (e.g., cannot focus on important things, mind wanders), guilt/worthlessness (e.g., disliking oneself, feeling like a bad person), sleep (e.g., restless sleep, trouble getting to sleep, sleeping much more), movement (e.g., feeling slow-moving, fidgety), appetite (e.g., poor appetite or losing weight without reason), and suicide (e.g., wanting to hurt oneself, wishing to be dead) (27). These 9 dimensions reflect the symptoms for diagnosis of clinical depression in the Diagnostic and Statistical Manual of Mental Disorders, Fifth Edition (DSM-5), whereby an individual is diagnosed with major depressive disorder if they experience 5 or more symptoms during the same 2-week period, with at least 1 of the symptoms being depressed mood/dysphoria or anhedonia (28). Participants rated the frequency of experiencing each symptom on the CESDR “in the past week or so” on a 5-point scale that included the response options “not at all or less than 1 day” (0), “1-2 days” (1), “3-4 days” (2), “5-7 days” (3), and “nearly every day for 2 weeks” (4). The sum of item scores was used to generate a global CESDR depression score ranging from 0 to 80. The depressive symptom category was determined using an algorithm that takes into account the frequencies of experiencing dysphoria or anhedonia in combination with other symptoms, as well as the CESD-style score that can be derived from the CESDR (27). The 5 categories from highest to lowest severity include (1) meeting the criteria for major depressive disorder, (2) probable major depressive disorder, (3) possible major depressive disorder, (4) subthreshold depression symptoms, and (5) no clinical significance. The CESDR has been shown to have good internal consistency (Cronbach’s α, range=0.90 to 0.93) (29, 30).

*Center for Epidemiologic Studies Anxiety (CESA) scale*

The CESA is a 20-item scale that was developed as a diagnostic screening tool for detecting anxiety disorder symptoms based on clinical criteria in the DSM-5 (31). The CESA comprised 3 sections (A, B, and C) in which participants rated the frequency of experiencing each anxiety symptom on a 4-point scale. Part A included 7 items assessing agoraphobia, social phobia, and blood-illness phobia, in which participants rated how afraid they were in different situations (e.g., “Were you afraid of being alone?”, “Were you afraid of being with people, even friends?”, etc.). Response options were “No, never” (0), “Yes, but never enough to change what I was planning or doing” (1), “Yes, and sometimes I avoided the situation” (2), and “Yes, and I avoided the situation almost all the time” (3). Part B included 12 items that evaluated symptoms of panic and anxiety disorders when participants were in the situations described in Part A (e.g., “get short of breath”, “tremble?”, “feel like throwing up”, etc.). Response options were “No, never” (0), “Yes, sometimes” (1), “Yes, often” (2), and “Yes, almost every time” (3). Part C included 1 item on whether the symptoms in Part B happened suddenly, with no clear explanation, even when not in one of the situations described in Part A. Response options were “No, never” (0), “Once or twice” (1), “Three times or more” (2), and “Many times” (3). The global score was derived by summing the item scores and could range from 0 to 60. A CESA score >16 with at least 1 response at level 3 was interpreted as evidence of anxiety disorder symptomology. The CESA showed good internal consistency (Cronbach’s alpha=0.84) and criterion validity based on a comparison with psychiatric evaluation of anxiety disorders (31).

**2. SUPPLEMENTARY RESULTS**

**2.1 Demographic characteristics and sleep behaviour**

**2.1.1 Study 1 in adolescents: Demographic characteristics and sleep behaviour across SHAWQ categories**

In our first study of adolescents whose data was used to develop the SHAWQ (*n*=1,733), the percentages of students categorized as having good, fair, and bad sleep health were 31.7%, 59.4%, and 8.9%, respectively. As expected, there were sex differences across SHAWQ categories (Chi-squared=76.2, *p*<0.001), in which the proportion of girls was higher in the fair and bad sleep health groups (60.8% and 60.4%) relative to the good sleep health group (38.3%). Additionally, students with poorer sleep health tended to be older, and small between-group differences were observed for ethnicity and type of school (**Supplementary Table 1a**).

By design, SHAWQ category was strongly associated with sleep health measures including sleep quality, daytime sleepiness, staying up until 3:00 am or later, and sleep latency on school days (Chi-squared ≥ 108.7, *p*<0.001 for all comparisons) (**Supplementary Table 1a**). Among students with bad sleep health, 70.1% reported that their sleep quality was bad or very bad, 50.0% indicated that daytime sleepiness was a big or very big problem, 44.1% stayed up until at least 3:00 am several times in the past 2 weeks, and 46.1% reported taking 30 min or longer to fall asleep on school nights. SHAWQ category was also associated with other sleep health-related measures including nocturnal awakenings, waking up earlier than desired, napping on schooldays or on weekends, and taking caffeine to stay awake during the day (Chi-squared ≥ 39.0, *p*<0.001 for all comparisons) (**Supplementary Table 1a**). In students with bad sleep health, 23.3% reported waking up at least 2 times on a typical night, 16.9% woke up earlier than desired at least several times in the past 2 weeks, 70.1% reported napping on school days, 51.4% reported napping on non-school days, and 58.3% reported taking caffeine at least once per week to help stay awake.

On school days, adolescents with higher SHAWQ scores went to bed later (SHAWQ category, mean bedtime ± SD: Good, 22:59 ± 01:06; Fair, 23:49 ± 01:10; Bad, 00:29 ± 01:24) (**Supplementary Table 1a**). However, wake-up times did not differ between SHAWQ categories (SHAWQ category, mean wake-up time ± SD: Good, 06:17 ± 00:32; Fair, 06:17 ± 00:36; Bad, 06:22 ± 00:37). Therefore, nocturnal sleep duration on school days was about an hour shorter in students with bad sleep health compared with good sleep health (SHAWQ category, mean nocturnal sleep duration ± SD: Good, 7.13 h ± 1.21 h; Fair, 6.24 h ± 1.16; Bad, 5.59 h ± 1.36 h).

On weekends/holidays, higher SHAWQ scores were associated with later bedtimes (SHAWQ category, mean bedtime ± SD: Good, 23:43 ± 01:13; Fair, 00:29 ± 01:25; Bad, 01:03 ± 01:44) and later wake-up times (SHAWQ category, mean wake-up time ± SD: Good, 08:48 ± 01:28; Fair, 09:24 ± 01:34; Bad, 10:01 ± 01:46) (**Supplementary Table 1a**). Higher SHAWQ scores were associated with marginally shorter nocturnal sleep duration (SHAWQ category, mean nocturnal sleep duration ± SD: Good, 8.98 h ± 1.43 h; Fair, 8.74 h ± 1.47; Bad, 8.68 h ± 2.02 h). The midpoint of the sleep period on weekends/holidays occurred more than an hour later in students with bad sleep health, suggesting that they had a later chronotype relative to their peers (**Supplementary Table 1a**).

**2.1.2 Study 2 in adolescents: Demographic characteristics and sleep behaviour across SHAWQ categories**

In our test population of adolescents (*n*=1,777), the percentages of students categorized as having good, fair, and bad sleep health were 12.0%, 67.8%, and 20.2%, respectively. There were sex differences across SHAWQ categories (Chi-squared=50.0, *p*<0.001), in which the proportion of girls was much higher in the bad and fair sleep health groups (61.6% and 53.4%) compared with the good sleep health group (31.5%). By comparison, small differences were observed for ethnicity, school type, and housing type across SHAWQ categories (**Supplementary Table 1b**).

As expected, individual responses on the SHAWQ and napping behaviour on school days and weekends differed across SHAWQ categories (Chi-squared ≥ 41.8, *p*<0.001 for all comparisons) (**Supplementary Table 1b**). In adolescents with bad sleep health, 82.2% reported that their sleep quality was bad or very bad, 21.7% indicated that daytime sleepiness was a big or very big problem, 66.3% stayed up until at least 3:00 am several times in the past 2 weeks, 64.4% reported taking 30 min or longer to fall asleep on school nights, 69.1% reported napping on school days, and 60.1% reported napping on non-school days.

On school days, adolescents with higher SHAWQ scores went to bed later (SHAWQ category, mean bedtime ± SD: Good, 23:32 ± 01:19; Fair, 00:20 ± 01:29; Bad, 01:14 ± 01:44), but they woke up only marginally later than their peers (SHAWQ category, mean wake-up time ± SD: Good, 06:31 ± 00:54; Fair, 06:39 ± 01:07; Bad, 06:46 ± 01:10). Consequently, nocturnal sleep duration on school days was about 90 min shorter in adolescents with bad sleep health compared with good sleep health (SHAWQ category, mean nocturnal sleep duration ± SD: Good, 6.75 h ± 1.34 h; Fair, 6.02 h ± 1.22; Bad, 5.16 h ± 1.38 h) (**Supplementary Table 1b**).

On weekends/holidays, higher SHAWQ scores were associated with later bedtimes (SHAWQ category, mean bedtime ± SD: Good, 00:38 ± 01:41; Fair, 01:46 ± 01:49; Bad, 03:00 ± 02:01) and later wake-up times (SHAWQ category, mean wake-up time ± SD: Good, 09:07 ± 01:44; Fair, 10:07 ± 01:59; Bad, 11:00 ± 02:25), but there were no between-group differences in nocturnal sleep duration (SHAWQ category, mean nocturnal sleep duration ± SD: Good, 8.55 h ± 1.73 h; Fair, 8.46 h ± 1.78; Bad, 8.26 h ± 2.16 h). The midpoint of the sleep period occurred much later in adolescents with bad sleep health, indicating that they had a later chronotype (**Supplemental Table 1b**).

**2.1.3 Study 3 in university students: Demographic characteristics and sleep behaviour across SHAWQ categories**

In our study of university students (*n*=2,040), the percentages of students categorized as having good, fair, and bad sleep health were 14.8%, 71.9%, and 13.4%. There were sex differences across SHAWQ categories (Chi-squared=48.0, *p*<0.001), in which the proportion of girls was much higher in the bad and fair sleep health groups (70.0% and 62.1%) compared with the good sleep health group (43.5%). Lower subjective SES, chronic medical problems, and receiving mental health care were associated with poorer sleep health, and there were small differences in ethnicity and class year across SHAWQ categories (**Supplementary Table 1c**).

Participants’ responses on individual SHAWQ items, napping behaviour, and caffeine intake differed across SHAWQ categories (Chi-squared ≥ 13.0, *p* ≤ 0.012 for all comparisons) (**Supplementary Table 1c**). In university students with bad sleep health, 74.4% reported that their sleep quality was bad or very bad, 22.0% indicated that daytime sleepiness was a big or very big problem, 65.6% stayed up until 3:00 am or later several times in the past 2 weeks, 63.4% reported taking 30 min or longer to fall asleep on school nights, 75.9% reported napping on school days, 50.9% reported napping on non-school days, and 45.1% took caffeine on a typical school day to help stay awake.

On school days, university students with higher SHAWQ scores went to bed later (SHAWQ category, mean bedtime ± SD: Good, 00:20 ± 01:07; Fair, 01:17 ± 01:19; Bad, 02:01 ± 01:26) and woke up later than their peers (SHAWQ category, mean wake-up time ± SD: Good, 08:08 ± 01:13; Fair, 08:41 ± 01:22; Bad, 09:05 ± 01:40). Between-group differences were greater for bedtime compared with wake-up time, resulting in shorter nocturnal sleep by about an hour in students with bad versus good sleep health (SHAWQ category, mean nocturnal sleep duration ± SD: Good, 7.57 h ± 0.88 h; Fair, 6.97 h ± 1.05; Bad, 6.47 h ± 1.18 h) (**Supplementary Table 1c**).

On weekends/holidays, higher SHAWQ scores were associated with later bedtimes (SHAWQ category, mean bedtime ± SD: Good, 00:48 ± 01:12; Fair, 01:38 ± 01:27; Bad, 02:23 ± 01:40) and later wake-up times (SHAWQ category, mean wake-up time ± SD: Good, 09:10 ± 01:25; Fair, 09:59 ± 01:37; Bad, 10:44 ± 02:01), but there were no between-group differences in nocturnal sleep duration (SHAWQ category, mean nocturnal sleep duration ± SD: Good, 8.22 h ± 0.97 h; Fair, 8.13 h ± 1.16; Bad, 8.03 h ± 1.30 h). Consequently, the midpoint of the sleep period occurred much later in students with bad sleep health, indicating that they had a later chronotype relative to the other sleep health groups (**Supplementary Table 1c**).

**2.1.4 Study 4 in university freshmen: Demographic characteristics and sleep behaviour across SHAWQ categories**

The percentages of students categorized as having good, fair, and bad sleep health were 11.5%, 61.9%, and 26.6% in freshmen who matriculated in 2020 (*n*=1,529) (**Supplementary Table 1d**), and 12.0%, 68.3%, and 19.7% in freshmen who matriculated in 2021 (*n*=1,488) (**Supplementary Table 1e**). The proportion of women with bad or fair sleep health (60.2% and 54.5%) was higher compared with good sleep health (37.5%) in the 2020 freshman cohort (Chi-squared=25.7, *p*<0.001), but did not differ significantly between SHAWQ categories in the 2021 freshman cohort (Chi-squared=4.3, *p*=0.119). There were no differences between SHAWQ categories in age, citizenship, ethnicity, type of housing, or school of enrolment in either freshman cohort (**Supplementary Table 1d-e**).

In both freshman cohorts, responses on individual SHAWQ items differed strongly between SHAWQ categories (2020 cohort: Chi-squared ≥ 270.2, *p*<0.001 for all comparisons; 2021 cohort: Chi-squared ≥ 308.5, *p*<0.001 for all comparisons) (**Supplementary Table 1d-e**). Among 2020 freshmen with bad sleep health, 81.8% reported that their sleep quality was bad or very bad, 32.9% indicated that daytime sleepiness was a big or very big problem, 73.5% stayed up until at least 3:00 am several times in the past 2 weeks, and 63.9% reported taking 30 min or longer to fall asleep on school nights. Similar results were observed in 2021 freshmen with bad sleep health, in whom 70.3% reported that their sleep quality was bad or very bad, 26.7% indicated that daytime sleepiness was a big or very big problem, 67.9% stayed up until at least 3:00 am several times in the past 2 weeks, and 76.8% reported taking 30 min or longer to fall asleep on school nights.

Sleep duration on school days differed significantly between SHAWQ categories (2020 cohort: Chi-squared=126.3, *p*<0.001; 2021 cohort: Chi-squared=87.2, *p*<0.001) (**Supplementary Table 1d-e**). Most students with good sleep health reported 7 to 9 hours of nocturnal sleep on school days (2020 cohort, 69.3%; 2021 cohort, 69.7%), whereas most students with bad sleep health reported <7 hours of nocturnal sleep (2020 cohort, 72.0%; 2021 cohort, 60.1%). Self-rated morningness-eveningness was also strongly associated with students’ SHAWQ scores (2020 cohort: Chi-squared=89.5, *p*<0.001; 2021 cohort: Chi-squared=76.7, *p*<0.001). Students with good sleep health were more likely to self-identify as a morning type in the 2020 freshman cohort (% of morning types by SHAWQ category: Good, 30.7%, Fair, 13.3%, Bad, 7.4) and the 2021 freshman cohort (% of morning types by SHAWQ category: Good, 29.2%, Fair, 12.7%, Bad, 6.1). By comparison, students with bad sleep health were more likely to self-identify as an evening type in the 2020 freshman cohort (% of evening types by SHAWQ category: Good, 8.5%, Fair, 18.9%, Bad, 31.9) and the 2021 freshman cohort (% of evening types by SHAWQ category: Good, 9.0%, Fair, 17.2%, Bad, 29.7%) (**Supplementary Table 1d-e**).

**2.2 Associations of the SHAWQ with depression symptoms and quality of life measures in university freshmen**

SHAWQ scores in both freshman cohorts were associated with severity of depression symptoms on the KADS (**Supplementary Fig. 4;** **Supplementary Table 3b**). Small-to-medium effect sizes were observed for fair versus good sleep health for fatigue/low motivation, sadness, lack of focus, and anxiety (2020 cohort: Cliff’s delta, range=0.26 to 0.41, *p*<0.001 for all comparisons; 2021 cohort: Cliff’s delta, range=0.21 to 0.32, *p*<0.001 for all comparisons), and large effect sizes were observed for all depression symptoms for students with bad versus good sleep health (2020 cohort: Cliff’s delta, range=0.52 to 0.70, *p*<0.001 for all comparisons; 2021 cohort: Cliff’s delta, range=0.50 to 0.60, *p*<0.001 for all comparisons).

SHAWQ scores were also associated with ratings of energy for everyday life and for satisfaction with sleep on the WHO Quality of Life questionnaire (**Supplementary Fig. 4;** **Supplementary Table 3b**). Effect sizes for energy for everyday life were small-to-medium for students with fair versus good sleep health (2020 cohort: Cliff’s delta=-0.33, 95% CI=-0.41 to -0.25, *p*<0.001; 2021 cohort: Cliff’s delta=-0.27, 95% CI=-0.34 to -0.18, *p*<0.001), and large for students with bad versus good sleep health (2020 cohort: Cliff’s delta=-0.63, 95% CI=-0.69 to -0.56, *p*<0.001; 2021 cohort: Cliff’s delta=-0.56, 95% CI=-0.63 to -0.47, *p*<0.001). Effect sizes were large for satisfaction with sleep for fair versus good sleep health (2020 cohort: Cliff’s delta=-0.58, 95% CI=-0.64 to -0.52, *p*<0.001; 2021 cohort: Cliff’s delta=-0.55, 95% CI=-0.61 to -0.48, *p*<0.001), and very large for bad versus good sleep health (2020 cohort: Cliff’s delta=-0.88, 95% CI=-0.92 to -0.84, *p*<0.001; 2021 cohort: Cliff’s delta=-0.82, 95% CI= -0.87 to -0.77, *p*<0.001).

**2.3 Associations of the SHAWQ with grades in university freshmen**

We assessed whether SHAWQ scores were associated with grades, adjusting for effects of age, ethnicity, citizenship, and school of enrolment at the university. ANCOVA showed that GPA and percentile rank differed significantly between SHAWQ categories in the 2020 freshman cohort (GPA: *F*_2, 1457_=7.657, *p*<0.001; percentile rank: *F*_2, 1457_=6.862, *p*=0.001) and the 2021 freshman cohort (GPA: *F*_2, 1402_=6.553, *p*=0.001; percentile rank: *F*_2, 1402_=5.545, *p*=0.004). Multiple comparison tests for percentile rank in the 2020 freshman cohort showed that students with bad sleep health had lower academic performance compared with students with either good sleep health (estimated difference=-8.24%, 95% CI=-14.35% to -2.13%; *t*=-3.16, *p*=0.005) or fair sleep health (estimated difference=-5.37%, 95% CI=-9.35% to -1.39%; *t*=-3.16, *p*=0.005), but the difference in performance between students with fair versus good sleep health did not reach statistical significance (estimated difference=-2.87%, 95% CI=-8.43% to 2.69%; *t*=-1.21, *p*=0.446). In the 2021 freshman cohort, the percentile rank of students with bad sleep health was nearly 10 percentage points lower compared with students with good sleep health after adjusting for covariates (estimated difference=-9.11%, 95% CI=-15.57% to -2.64%; *t*=-3.31, *p*=0.003), whereas performance did not differ significantly between students with bad versus fair sleep health (estimated difference=-3.98%, 95% CI=-8.44% to 0.474; *t*=-2.10, *p*=0.091), or between students with fair versus good sleep health (estimated difference=-5.13%, 95% CI=-10.69% to 0.44%; *t*=-2.16, *p*=0.079).

**3. SUPPLEMENTARY DISCUSSION**

**3.1 Internal consistency of the SHAWQ**

We did not test for internal consistency (e.g., Cronbach’s alpha) of the SHAWQ because of the way that the questionnaire was developed and scored. Tests of internal consistency assume that the instrument assesses a single construct (i.e., the items are unidimensional or homogenous) (32). Violations of this assumption can result in underestimation of the reliability, especially if the instrument includes a small number of items. The SHAWQ included items assessing multidimensional sleep health, as well as questions on self-rated health and gender. Cronbach’s alpha should not be used in such instances where the instrument is multifaceted (33). Additionally, the SHAWQ items were selected to have the least amount of overlap in predicting depression scores and were not intended to sample the entire domain of sleep health. Cronbach’s alpha is also typically used when items are scored using the same type of scale (e.g., a Likert scale or standardized component scores). In contrast, the number of response items and their scoring differed for each question on the SHAWQ.

**3.2 Multidimensional sleep health is associated with mental health in adults**

Our findings for the SHAWQ and mental health in adolescents and university students are consistent with studies of multidimensional sleep health in older adult populations. In online cross-sectional surveys of respondents whose ages spanned several decades, better sleep health using the RuSATED sleep health framework was associated with fewer depression symptoms on the Hospital Anxiety and Depression Scale (34), and lower likelihood of reporting current or chronic symptoms of depression, anxiety, or daytime dysfunction on the EuroQol 5-dimension scale (35). In the latter study, the sleep dimensions that were most strongly associated with mental well-being were satisfaction with sleep, daytime alertness, and sleep efficiency. In a low-income predominantly African American population, better composite sleep health scores based on actigraphy (regularity, timing, efficiency and duration of sleep) and satisfaction with sleep were associated with lower scores on the 6-item Kessler Psychological Distress Scale (K6); however, only the sleep satisfaction variable was significantly associated with K6 scores (36).

Studies that have focused on midlife and older adults have also demonstrated a strong link between sleep health problems and depression symptoms. In the Midlife in the United States (MIDUS) study, higher PSQI scores were associated with depression symptoms on the CESD, and the components that were most strongly associated with depression scores were subjective sleep quality, sleep disturbances, and daytime dysfunction (37). In a subset of MIDUS study participants with actigraphy and self-report measures, poorer composite sleep health scores were associated with more frequent stress symptoms on the Perceived Stress Scale (38). Adjusting for covariates, sleep regularity and satisfaction with sleep explained additional variance in perceived stress, whereas contributions of alertness, sleep timing, sleep efficiency, and sleep duration were negligible. In the Study of Women’s Health Across the Nation, midlife women with poorer composite sleep health scores (based on actigraphy and questionnaires) also reported greater severity of depression symptoms, whereby higher CESD scores were associated with later sleep timing, lower sleep efficiency, and higher daytime sleepiness, but not shorter sleep duration or lower sleep regularity (39). Among older women in the in the Study of Osteoporotic Fractures, composite scores of poor sleep health using the SATED framework were associated with incident depression using the Geriatric Depression Scale, and the dimensions associated with incident depression symptoms were sleep satisfaction, daytime sleepiness, and sleep onset latency (40).

**3.3 Different dimensions of sleep health are associated with grades**

Our findings for the SHAWQ are consistent with previous studies in adolescents and university students demonstrating that different dimensions of sleep health are associated with academic performance. While there have been many studies linking sleep-related variables (e.g., duration, timing, irregularity, efficiency, quality, and daytime sleepiness) with school and learning outcomes (41-44), here we focus on some of the key findings that are most relevant to items in the SHAWQ. Meta-analyses have shown that poorer sleep quality is associated with lower academic performance in children and adolescents (45, 46), although the definition for sleep quality varied substantially among the included studies. In adolescents, higher daytime sleepiness scores on the ESS or the Paediatric Daytime Sleepiness Scale have been shown to associate with final exam scores and self-reported GPA (47, 48). Adolescents who reported feeling sleepy during the first hours at school have also been shown to have poorer objective school performance based on end-of-term grades (49). Related work in university students found that impaired daytime functioning caused by insufficient sleep was longitudinally associated with end-of-semester GPA (50). Late bedtimes are also thought to contribute to insufficient nocturnal sleep and lower academic performance. In a study of nearly 12,000 university students across 50 universities in China, lower self-reported grades were found among students who went to bed later (51). Similar findings were reported in a longitudinal study of Canadian university students in whom bedtimes were later in semesters that they had a lower grade point average (52). Moreover, meta-analyses have shown that later preferred sleep timing (i.e., greater eveningness) is associated with poorer academic performance in high school and university students (53-55). Together, these studies show that the items included in the SHAWQ are relevant for school performance.

**4. References**

1. Thapar A, Collishaw S, Pine DS, Thapar AK. Depression in adolescence. The Lancet. 2012;379(9820):1056-67.

2. Gariepy G, Danna S, Gobiņa I, Rasmussen M, Gaspar de Matos M, Tynjälä J, et al. How Are Adolescents Sleeping? Adolescent Sleep Patterns and Sociodemographic Differences in 24 European and North American Countries. Journal of Adolescent Health. 2020;66(6, Supplement):S81-S8.

3. Gradisar M, Gardner G, Dohnt H. Recent worldwide sleep patterns and problems during adolescence: A review and meta-analysis of age, region, and sleep. Sleep Medicine. 2011;12(2):110-8.

4. Twenge JM, Nolen-Hoeksema S. Age, gender, race, socioeconomic status, and birth cohort difference on the children's depression inventory: A meta-analysis. Journal of Abnormal Psychology. 2002;111(4):578-88.

5. Conklin AI, Yao CA, Richardson CG. Chronic sleep disturbance, not chronic sleep deprivation, is associated with self-rated health in adolescents. Preventive Medicine. 2019;124:11-6.

6. Klein DN, Glenn CR, Kosty DB, Seeley JR, Rohde P, Lewinsohn PM. Predictors of First Lifetime Onset of Major Depressive Disorder in Young Adulthood. Journal of abnormal psychology. 2013;122(1):1-6.

7. Ambresin G, Chondros P, Dowrick C, Herrman H, Gunn JM. Self-Rated Health and Long-Term Prognosis of Depression. The Annals of Family Medicine. 2014;12(1):57-65.

8. Alonzo R, Hussain J, Stranges S, Anderson KK. Interplay between social media use, sleep quality, and mental health in youth: A systematic review. Sleep Medicine Reviews. 2021;56:101414.

9. Buysse DJ, Reynolds CF, Monk TH, Berman SR, Kupfer DJ. The Pittsburgh sleep quality index: A new instrument for psychiatric practice and research. Psychiatry Research. 1989;28(2):193-213.

10. Wolfson AR, Carskadon MA. Sleep Schedules and Daytime Functioning in Adolescents. Child Development. 1998;69(4):875-87.

11. Brooks SJ, Krulewicz SP, Kutcher S. The Kutcher Adolescent Depression Scale: Assessment of Its Evaluative Properties over the Course of an 8-Week Pediatric Pharmacotherapy Trial. Journal of Child and Adolescent Psychopharmacology. 2003;13(3):337-49.

12. LeBlanc JC, Almudevar A, Brooks SJ, Kutcher S. Screening for Adolescent Depression: Comparison of the Kutcher Adolescent Depression Scale with the Beck Depression Inventory. Journal of Child and Adolescent Psychopharmacology. 2002;12(2):113-26.

13. Zhou H, Hao N, Liu Y, Sui Y, Wang Y, Ciu Y. Reliability and validity of the eleven item Kutcher Adolescent Depression Scale, Chinese Version (KADS-11CV). J Child Adolesc Behav. 2016;4(4):10.4172.

14. Roenneberg T, Wirz-Justice A, Merrow M. Life between Clocks: Daily Temporal Patterns of Human Chronotypes. Journal of Biological Rhythms. 2003;18(1):80-90.

15. Johns MW. A New Method for Measuring Daytime Sleepiness: The Epworth Sleepiness Scale. Sleep. 1991;14(6):540-5.

16. Izci B, Ardic S, Firat H, Sahin A, Altinors M, Karacan I. Reliability and validity studies of the Turkish version of the Epworth Sleepiness Scale. Sleep and Breathing. 2008;12(2):161-8.

17. Hagell P, Broman J-E. Measurement properties and hierarchical item structure of the Epworth Sleepiness Scale in Parkinson's disease. Journal of Sleep Research. 2007;16(1):102-9.

18. Cho YW, Lee JH, Son HK, Lee SH, Shin C, Johns MW. The reliability and validity of the Korean version of the Epworth sleepiness scale. Sleep and Breathing. 2011;15(3):377-84.

19. Kendzerska TB, Smith PM, Brignardello-Petersen R, Leung RS, Tomlinson GA. Evaluation of the measurement properties of the Epworth sleepiness scale: A systematic review. Sleep Medicine Reviews. 2014;18(4):321-31.

20. Johns MW. Reliability and Factor Analysis of the Epworth Sleepiness Scale. Sleep. 1992;15(4):376-81.

21. van der Heide A, van Schie MKM, Lammers GJ, Dauvilliers Y, Arnulf I, Mayer G, et al. Comparing Treatment Effect Measurements in Narcolepsy: The Sustained Attention to Response Task, Epworth Sleepiness Scale and Maintenance of Wakefulness Test. Sleep. 2015;38(7):1051-8.

22. Gibson ES, Powles AP, Thabane L, O'Brien S, Molnar DS, Trajanovic N, et al. "Sleepiness" is serious in adolescence: Two surveys of 3235 Canadian students. BMC Public Health. 2006;6(1):1-9.

23. Mollayeva T, Thurairajah P, Burton K, Mollayeva S, Shapiro CM, Colantonio A. The Pittsburgh sleep quality index as a screening tool for sleep dysfunction in clinical and non-clinical samples: A systematic review and meta-analysis. Sleep Medicine Reviews. 2016;25:52-73.

24. Bastien CH, Vallières A, Morin CM. Validation of the Insomnia Severity Index as an outcome measure for insomnia research. Sleep Medicine. 2001;2(4):297-307.

25. Morin CM, Belleville G, Bélanger L, Ivers H. The Insomnia Severity Index: Psychometric Indicators to Detect Insomnia Cases and Evaluate Treatment Response. Sleep. 2011;34(5):601-8.

26. Chahoud M, Chahine R, Salameh P, Sauleau EA. Reliability, factor analysis and internal consistency calculation of the Insomnia Severity Index (ISI) in French and in English among Lebanese adolescents. eNeurologicalSci. 2017;7:9-14.

27. Eaton WW, Smith C, Ybarra M, Muntaner C, Tien A. Center for Epidemiologic Studies Depression Scale: Review and Revision (CESD and CESD-R). The use of psychological testing for treatment planning and outcomes assessment: Instruments for adults, Volume 3, 3rd ed. Mahwah, NJ, US: Lawrence Erlbaum Associates Publishers; 2004. p. 363-77.

28. American Psychiatric Association A, Association AP. Diagnostic and statistical manual of mental disorders: DSM-5: Washington, DC: American psychiatric association; 2013.

29. Van Dam NT, Earleywine M. Validation of the Center for Epidemiologic Studies Depression Scale—Revised (CESD-R): Pragmatic depression assessment in the general population. Psychiatry Research. 2011;186(1):128-32.

30. Tran TD, Kaligis F, Wiguna T, Willenberg L, Nguyen HTM, Luchters S, et al. Screening for depressive and anxiety disorders among adolescents in Indonesia: Formal validation of the centre for epidemiologic studies depression scale – revised and the Kessler psychological distress scale. Journal of Affective Disorders. 2019;246:189-94.

31. Faro A, Eaton WW. A Diagnostic-Oriented Screening Scale for Anxiety Disorders: The Center for Epidemiologic Studies Anxiety Scale (CESA). Frontiers in Psychology. 2020;11:957.

32. Tavakol M, Dennick R. Making sense of Cronbach's alpha. International Journal of Medical Education. 2011;2:53-5.

33. Streiner DL. Starting at the beginning: an introduction to coefficient alpha and internal consistency. Journal of Personality Assessment. 2003;80(1):99-103.

34. Barham WT, Buysse DJ, Kline CE, Kubala AG, Brindle RC. Sleep health mediates the relationship between physical activity and depression symptoms. Sleep & Breathing = Schlaf & Atmung. 2022;26(3):1341-9.

35. Appleton SL, Melaku YA, Reynolds AC, Gill TK, de Batlle J, Adams RJ. Multidimensional sleep health is associated with mental well-being in Australian adults. Journal of Sleep Research. 2022;31(2):e13477.

36. DeSantis AS, Dubowitz T, Ghosh-Dastidar B, Hunter GP, Buman M, Buysse DJ, et al. A preliminary study of a composite sleep health score: associations with psychological distress, body mass index, and physical functioning in a low-income African American community. Sleep Health. 2019;5(5):514-20.

37. Huang Y, Zhu M. Increased Global PSQI Score Is Associated with Depressive Symptoms in an Adult Population from the United States. Nat Sci Sleep. 2020;12:487-95.

38. Lee S, Lawson KM. Beyond single sleep measures: A composite measure of sleep health and its associations with psychological and physical well-being in adulthood. Social Science & Medicine. 2021;274:113800.

39. Bowman MA, Kline CE, Buysse DJ, Kravitz HM, Joffe H, Matthews KA, et al. Longitudinal Association Between Depressive Symptoms and Multidimensional Sleep Health: The SWAN Sleep Study. Annals of Behavioral Medicine. 2021;55(7):641-52.

40. Furihata R, Hall MH, Stone KL, Ancoli-Israel S, Smagula SF, Cauley JA, et al. An Aggregate Measure of Sleep Health Is Associated With Prevalent and Incident Clinically Significant Depression Symptoms Among Community-Dwelling Older Women. Sleep. 2017;40(3):zsw075.

41. Curcio G, Ferrara M, De Gennaro L. Sleep loss, learning capacity and academic performance. Sleep Medicine Reviews. 2006;10(5):323-37.

42. Prichard JR. Sleep Predicts Collegiate Academic Performance. Sleep Medicine Clinics. 2020;15(1):59-69.

43. Schmidt RE, Van der Linden M. The Relations Between Sleep, Personality, Behavioral Problems, and School Performance in Adolescents. Sleep Medicine Clinics. 2015;10(2):117-23.

44. Wolfson AR, Carskadon MA. Understanding adolescent's sleep patterns and school performance: a critical appraisal. Sleep Medicine Reviews. 2003;7(6):491-506.

45. Dewald JF, Meijer AM, Oort FJ, Kerkhof GA, Bögels SM. The influence of sleep quality, sleep duration and sleepiness on school performance in children and adolescents: A meta-analytic review. Sleep Medicine Reviews. 2010;14(3):179-89.

46. Musshafen LA, Tyrone RS, Abdelaziz A, Sims-Gomillia CE, Pongetti LS, Teng F, et al. Associations between sleep and academic performance in US adolescents: a systematic review and meta-analysis. Sleep Medicine. 2021;83:71-82.

47. Rhie S, Lee S, Chae KY. Sleep patterns and school performance of Korean adolescents assessed using a Korean version of the pediatric daytime sleepiness scale. Korean Journal of Pediatrics. 2011;54(1):29-35.

48. Shin C, Kim J, Lee S, Ahn Y, Joo S. Sleep habits, excessive daytime sleepiness and school performance in high school students. Psychiatry and Clinical Neurosciences. 2003;57(4):451-3.

49. Boschloo A, Krabbendam L, Dekker S, Lee N, de Groot R, Jolles J. Subjective Sleepiness and Sleep Quality in Adolescents are Related to Objective and Subjective Measures of School Performance. Frontiers in Psychology. 2013;4.

50. Chen W-L, Chen J-H. Consequences of inadequate sleep during the college years: Sleep deprivation, grade point average, and college graduation. Preventive Medicine. 2019;124:23-8.

51. Wu D, Yang T. Late bedtime, uncertainty stress among Chinese college students: impact on academic performance and self-rated health. Psychology, Health & Medicine. 2022;0(0):1-12.

52. Galambos NL, Vargas Lascano DI, Howard AL, Maggs JL. Who Sleeps Best? Longitudinal Patterns and Covariates of Change in Sleep Quantity, Quality, and Timing Across Four University Years. Behavioral Sleep Medicine. 2013;11(1):8-22.

53. Zerbini G, Merrow M. Time to learn: How chronotype impacts education: Time to learn: How chronotype impacts education. PsyCh Journal. 2017;6(4):263-76.

54. Tonetti L, Natale V, Randler C. Association between circadian preference and academic achievement: A systematic review and meta-analysis. Chronobiology International. 2015;32(6):792-801.

55. Preckel F, Lipnevich AA, Schneider S, Roberts RD. Chronotype, cognitive abilities, and academic achievement: A meta-analytic investigation. Learning and Individual Differences. 2011;21(5):483-92.

**SUPPLEMENTARY TABLES**

**Supplementary Table 1: Demographic and sleep characteristics**

**Supplementary Table 1a: Study 1 in adolescents (analysis of previous study)**

|  |  | **SHAWQ category of sleep health** | | |  |  |
| --- | --- | --- | --- | --- | --- | --- |
| **Characteristic** | **All (n = 1733)** | **Good**  **(n = 549)** | **Fair**  **(n = 1030)** | **Bad**  **(n = 154)** | ***X*^2^*/F*** | ***p*** |
| **Girls, *n* (%)** | 929 (53.6) | 210 (38.3) | 626 (60.8) | 93 (60.4) | 76.2 | <0.001 |
| **Age (years)** | 16.08 ± 1.52 | 15.62 ± 1.62 | 16.28 ± 1.43 | 16.40 ± 1.40 | 39.5 | <0.001 |
| **Ethnicity, *n* (%)** |  |  |  |  |  |  |
| Chinese | 1230 (71.3) | 367 (67.3) | 759 (74.0) | 104 (68.0) | 14.7 | 0.023 |
| Malay | 27 (1.6) | 7 (1.3) | 15 (1.5) | 5 (3.3) |  |  |
| Indian | 105 (6.1) | 37 (6.8) | 62 (6.0) | 6 (3.9) |  |  |
| Others | 362 (21.0) | 134 (24.6) | 190 (18.5) | 38 (24.8) |  |  |
| **Local school, *n* (%)** | 1226 (70.7) | 355 (64.7) | 767 (74.5) | 104 (67.5) | 17.5 | <0.001 |
| **Self-rated health, *n* (%)** |  |  |  |  |  |  |
| Excellent | 273 (15.8) | 164 (29.9) | 100 (9.7) | 9 (5.8) | 514.3 | <0.001 |
| Good | 958 (55.3) | 341 (62.1) | 572 (55.5) | 45 (29.2) |  |  |
| Fair | 457 (26.4) | 44 (8.0) | 349 (33.9) | 64 (41.6) |  |  |
| Poor | 45 (2.6) | 0 (0.0) | 9 (0.9) | 36 (23.4) |  |  |
| **Sleep quality, *n* (%)** |  |  |  |  |  |  |
| Very good | 251 (14.5) | 166 (30.2) | 85 (8.3) | 0 (0.0) | 1147.4 | <0.001 |
| Good | 645 (37.2) | 368 (67.0) | 275 (26.7) | 2 (1.3) |  |  |
| Okay | 604 (34.9) | 15 (2.7) | 545 (52.9) | 44 (28.6) |  |  |
| Bad | 206 (11.9) | 0 (0.0) | 123 (11.9) | 83 (53.9) |  |  |
| Very bad | 27 (1.6) | 0 (0.0) | 2 (0.2) | 25 (16.2) |  |  |
| **Sleepiness problem, *n* (%)** |  |  |  |  |  |  |
| No problem at all | 204 (11.8) | 148 (27.0) | 55 (5.3) | 1 (0.6) | 692.2 | <0.001 |
| A little problem | 919 (53.0) | 372 (67.8) | 535 (51.9) | 12 (7.8) |  |  |
| More than a little problem | 422 (24.4) | 29 (5.3) | 329 (31.9) | 64 (41.6) |  |  |
| A big problem | 132 (7.6) | 0 (0.0) | 90 (8.7) | 42 (27.3) |  |  |
| A very big problem | 56 (3.2) | 0 (0.0) | 21 (2.0) | 35 (22.7) |  |  |
| **Stayed up past 3:00 am, *n* (%)** |  |  |  |  |  |  |
| Never | 1124 (64.9) | 490 (89.3) | 610 (59.2) | 24 (15.6) | 367.0 | <0.001 |
| Once or twice | 393 (22.7) | 53 (9.7) | 278 (27.0) | 62 (40.3) |  |  |
| Several times | 197 (11.4) | 5 (0.9) | 131 (12.7) | 61 (39.6) |  |  |
| Every night | 19 (1.1) | 1 (0.2) | 11 (1.1) | 7 (4.5) |  |  |
| **Sleep latency, *n* (%)** |  |  |  |  |  |  |
| Less than 30 minutes | 1340 (77.3) | 490 (89.3) | 767 (74.5) | 83 (53.9) | 108.7 | <0.001 |
| 30 to 60 minutes | 372 (21.5) | 58 (10.6) | 251 (24.4) | 63 (40.9) |  |  |
| More than 60 minutes | 21 (1.2) | 1 (0.2) | 12 (1.2) | 8 (5.2) |  |  |
| **Wake up during night, *n* (%)** |  |  |  |  |  |  |
| Never | 971 (56.0) | 335 (61.0) | 577 (56.0) | 59 (38.3) | 95.6 | <0.001 |
| Once | 432 (24.9) | 137 (25.0) | 252 (24.5) | 43 (27.9) |  |  |
| 2 or 3 times | 160 (9.2) | 31 (5.6) | 110 (10.7) | 19 (12.3) |  |  |
| More than 3 times | 35 (2.0) | 2 (0.4) | 16 (1.6) | 17 (11.0) |  |  |
| I have no idea | 135 (7.8) | 44 (8.0) | 75 (7.3) | 16 (10.4) |  |  |
| **Wake up too early, *n* (%)** |  |  |  |  |  |  |
| Never | 1137 (65.6) | 383 (69.8) | 664 (64.5) | 90 (58.4) | 39.0 | <0.001 |
| Once or twice | 474 (27.4) | 150 (27.3) | 286 (27.8) | 38 (24.7) |  |  |
| Several times | 113 (6.5) | 14 (2.6) | 75 (7.3) | 24 (15.6) |  |  |
| Every day/night | 9 (0.5) | 2 (0.4) | 5 (0.5) | 2 (1.3) |  |  |
| **School day naps, *n* (%)** |  |  |  |  |  |  |
| I never nap | 897 (51.8) | 349 (63.6) | 502 (48.7) | 46 (29.9) | 100.7 | <0.001 |
| 1-2 schooldays/ week | 564 (32.5) | 160 (29.1) | 347 (33.7) | 57 (37.0) |  |  |
| 3-4 school days/ week | 171 (9.9) | 28 (5.1) | 118 (11.5) | 25 (16.2) |  |  |
| Every day | 101 (5.8) | 12 (2.2) | 63 (6.1) | 26 (16.9) |  |  |
| **Weekend naps, *n* (%)** |  |  |  |  |  |  |
| I don't nap | 1070 (61.7) | 386 (70.3) | 609 (59.1) | 75 (48.7) | 44.4 | <0.001 |
| Once | 456 (26.3) | 118 (21.5) | 296 (28.7) | 42 (27.3) |  |  |
| Twice or more | 207 (11.9) | 45 (8.2) | 125 (12.1) | 37 (24.0) |  |  |
| **Caffeine to stay awake, *n* (%)** |  |  |  |  |  |  |
| Never | 1035 (59.7) | 375 (68.3) | 596 (57.9) | 64 (41.6) | 70.2 | <0.001 |
| 1 or 2 days | 394 (22.7) | 120 (21.9) | 239 (23.2) | 35 (22.7) |  |  |
| 3 or 4 days | 136 (7.8) | 29 (5.3) | 86 (8.3) | 21 (13.6) |  |  |
| 5 or 6 days | 56 (3.2) | 7 (1.3) | 40 (3.9) | 9 (5.8) |  |  |
| Every day | 112 (6.5) | 18 (3.3) | 69 (6.7) | 25 (16.2) |  |  |
| **Sleep on school days** |  |  |  |  |  |  |
| Bedtime (hh:mm) | 23:37 ± 01:15 | 22:59 ± 01:06 | 23:49 ± 01:10 | 00:29 ± 01:24 | 139 | <0.001 |
| Wake-up time (hh:mm) | 06:17 ± 00:35 | 06:17 ± 00:32 | 06:17 ± 00:36 | 06:22 ± 00:37 | 1.4 | 0.246 |
| Nocturnal sleep duration (h) | 6.46 ± 1.29 | 7.13 ± 1.21 | 6.24 ± 1.16 | 5.59 ± 1.36 | 144.5 | <0.001 |
| **Sleep on weekends** |  |  |  |  |  |  |
| Bedtime (hh:mm) | 00:17 ± 01:27 | 23:43 ± 01:13 | 00:29 ± 01:25 | 01:03 ± 01:44 | 80.5 | <0.001 |
| Wake-up time (hh:mm) | 09:16 ± 01:35 | 08:48 ± 01:28 | 09:24 ± 01:34 | 10:01 ± 01:46 | 46.0 | <0.001 |
| Nocturnal sleep duration (h) | 8.81 ± 1.52 | 8.98 ± 1.43 | 8.74 ± 1.47 | 8.68 ± 2.02 | 5.1 | 0.006 |
| Midpoint of sleep (hh:mm) | 04:47 ± 01:20 | 04:16 ± 01:08 | 04:56 ± 01:18 | 05:32 ± 01:28 | 81.7 | <0.001 |

**Supplementary Table 1b: Study 2 in adolescents (cross-sectional study)**

|  |  | **SHAWQ category of sleep health** | | |  |  |
| --- | --- | --- | --- | --- | --- | --- |
| **Characteristic** | **All (n = 1777)** | **Good**  **(n = 213)** | **Fair**  **(n = 1205)** | **Bad**  **(n = 359)** | ***X*^2^*/F*** | ***p*** |
| **Girls, *n* (%)** | 931 (52.4) | 67 (31.5) | 643 (53.4) | 221 (61.6) | 50.0 | <0.001 |
| **Age (years)** | 17.27 ± 1.19 | 17.42 ± 1.11 | 17.23 ± 1.23 | 17.33 ± 1.08 | 2.7 | 0.068 |
| **Ethnicity, *n* (%)** |  |  |  |  |  |  |
| Chinese | 1037 (65.7) | 126 (67.7) | 725 (67.8) | 186 (57.4) | 23.6 | 0.001 |
| Malay | 308 (19.5) | 29 (15.6) | 190 (17.8) | 89 (27.5) |  |  |
| Indian | 156 (9.9) | 25 (13.4) | 103 (9.6) | 28 (8.6) |  |  |
| Others | 78 (4.9) | 6 (3.2) | 51 (4.8) | 21 (6.5) |  |  |
| **School type, *n* (%)** |  |  |  |  |  |  |
| Secondary school | 232 (13.1) | 22 (10.3) | 177 (14.7) | 33 (9.2) | 28.4 | <0.001 |
| Junior college | 552 (31.1) | 64 (30.0) | 389 (32.3) | 99 (27.6) |  |  |
| Polytechnic | 297 (16.7) | 51 (23.9) | 194 (16.1) | 52 (14.5) |  |  |
| Institute of Technical Education | 696 (39.2) | 76 (35.7) | 445 (36.9) | 175 (48.7) |  |  |
| **Housing type, *n* (%)** |  |  |  |  |  |  |
| Public 1- or 2-room flat | 79 (4.6) | 6 (2.9) | 62 (5.3) | 11 (3.1) | 32.9 | <0.001 |
| Public 3-room flat | 237 (13.7) | 31 (15.2) | 137 (11.7) | 69 (19.5) |  |  |
| Public 4-room flat | 552 (31.9) | 50 (24.5) | 379 (32.3) | 123 (34.8) |  |  |
| Public Executive/ 5-room flat | 469 (27.1) | 58 (28.4) | 318 (27.1) | 93 (26.3) |  |  |
| Condo/ Private | 292 (16.9) | 46 (22.5) | 204 (17.4) | 42 (11.9) |  |  |
| Landed property | 103 (5.9) | 13 (6.4) | 75 (6.4) | 15 (4.2) |  |  |
| **Self-rated health, *n* (%)** |  |  |  |  |  |  |
| Excellent | 218 (12.3) | 68 (31.9) | 141 (11.7) | 9 (2.5) | 422.5 | <0.001 |
| Good | 990 (55.7) | 135 (63.4) | 738 (61.2) | 117 (32.6) |  |  |
| Fair | 498 (28.0) | 10 (4.7) | 316 (26.2) | 172 (47.9) |  |  |
| Poor | 71 (4.0) | 0 (0.0) | 10 (0.8) | 61 (17.0) |  |  |
| **Sleep quality, *n* (%)** |  |  |  |  |  |  |
| Good | 278 (15.6) | 172 (80.8) | 104 (8.6) | 2 (0.6) | 1477.4 | <0.001 |
| Okay | 989 (55.7) | 41 (19.2) | 886 (73.5) | 62 (17.3) |  |  |
| Bad | 377 (21.2) | 0 (0.0) | 203 (16.8) | 174 (48.5) |  |  |
| Very bad | 133 (7.5) | 0 (0.0) | 12 (1.0) | 121 (33.7) |  |  |
| **Sleepiness problem, *n* (%)** |  |  |  |  |  |  |
| No problem at all | 402 (22.6) | 131 (61.5) | 249 (20.7) | 22 (6.1) | 505.4 | <0.001 |
| A little problem | 966 (54.4) | 81 (38.0) | 745 (61.8) | 140 (39.0) |  |  |
| More than a little problem | 297 (16.7) | 1 (0.5) | 177 (14.7) | 119 (33.1) |  |  |
| A big problem | 72 (4.1) | 0 (0.0) | 29 (2.4) | 43 (12.0) |  |  |
| A very big problem | 40 (2.3) | 0 (0.0) | 5 (0.4) | 35 (9.7) |  |  |
| **Stayed up past 3:00 am, *n* (%)** |  |  |  |  |  |  |
| Never | 623 (35.1) | 161 (75.6) | 432 (35.9) | 30 (8.4) | 367.9 | <0.001 |
| Once or twice | 560 (31.5) | 34 (16.0) | 435 (36.1) | 91 (25.3) |  |  |
| Several times | 594 (33.4) | 18 (8.5) | 338 (28.0) | 238 (66.3) |  |  |
| **Sleep latency, *n* (%)** |  |  |  |  |  |  |
| Less than 30 minutes | 1148 (64.6) | 193 (90.6) | 827 (68.6) | 128 (35.7) | 295.7 | <0.001 |
| 30 to 60 minutes | 513 (28.9) | 19 (8.9) | 343 (28.5) | 151 (42.1) |  |  |
| More than 60 minutes | 116 (6.5) | 1 (0.5) | 35 (2.9) | 80 (22.3) |  |  |
| **School day naps, *n* (%)** |  |  |  |  |  |  |
| 0 school days/ week | 671 (37.9) | 115 (54.2) | 445 (37.1) | 111 (30.9) | 49.6 | <0.001 |
| 1-2 schooldays/ week | 633 (35.8) | 60 (28.3) | 452 (37.7) | 121 (33.7) |  |  |
| 3-4 school days/ week | 253 (14.3) | 15 (7.1) | 177 (14.8) | 61 (17.0) |  |  |
| Every day of school/ week | 213 (12.0) | 22 (10.4) | 125 (10.4) | 66 (18.4) |  |  |
| **Weekend naps, *n* (%)** |  |  |  |  |  |  |
| Not at all | 875 (49.4) | 128 (60.4) | 604 (50.2) | 143 (39.9) | 41.8 | <0.001 |
| Once | 556 (31.4) | 56 (26.4) | 392 (32.6) | 108 (30.2) |  |  |
| Twice or more | 342 (19.3) | 28 (13.2) | 207 (17.2) | 107 (29.9) |  |  |
| **Sleep on school days** |  |  |  |  |  |  |
| Bedtime (hh:mm) | 00:24 ± 01:35 | 23:32 ± 01:19 | 00:20 ± 01:29 | 01:14 ± 01:44 | 79.2 | <0.001 |
| Wake-up time (hh:mm) | 06:39 ± 01:06 | 06:31 ± 00:54 | 06:39 ± 01:07 | 06:46 ± 01:10 | 3.6 | 0.027 |
| Nocturnal sleep duration (h) | 5.93 ± 1.34 | 6.75 ± 1.34 | 6.02 ± 1.22 | 5.16 ± 1.38 | 109.8 | <0.001 |
| **Sleep on weekends** |  |  |  |  |  |  |
| Bedtime (hh:mm) | 01:53 ± 01:58 | 00:38 ± 01:41 | 01:46 ± 01:49 | 03:00 ± 02:01 | 111.4 | <0.001 |
| Wake-up time (hh:mm) | 10:10 ± 02:07 | 09:07 ± 01:44 | 10:07 ± 01:59 | 11:00 ± 02:25 | 57.7 | <0.001 |
| Nocturnal sleep duration (h) | 8.43 ± 1.86 | 8.55 ± 1.73 | 8.46 ± 1.78 | 8.26 ± 2.16 | 2.1 | 0.125 |
| Midpoint of sleep (hh:mm) | 06:04 ± 01:50 | 04:53 ± 01:29 | 05:58 ± 01:41 | 07:04 ± 02:00 | 103.2 | <0.001 |

**Supplementary Table 1c: Study 3 in university students (cross-sectional study)**

|  |  | **SHAWQ category of sleep health** | | |  |  |
| --- | --- | --- | --- | --- | --- | --- |
| **Characteristic** | **All (n = 2040)** | **Good**  **(n = 301)** | **Fair**  **(n = 1466)** | **Bad**  **(n = 273)** | ***X*^2^*/F*** | ***p*** |
| **Girls, *n* (%)** | 1232 (60.4) | 131 (43.5) | 910 (62.1) | 191 (70.0) | 48.0 | <0.001 |
| **Age (years)** | 21.60 ± 2.12 | 22.02 ± 3.00 | 21.50 ± 1.90 | 21.68 ± 1.97 | 7.8 | <0.001 |
| **Ethnicity, *n* (%)** |  |  |  |  |  |  |
| Chinese | 1832 (89.8) | 271 (90.0) | 1325 (90.4) | 236 (86.4) | 18.4 | 0.019 |
| Malay | 41 (2.0) | 7 (2.3) | 20 (1.4) | 14 (5.1) |  |  |
| Indian | 90 (4.4) | 10 (3.3) | 68 (4.6) | 12 (4.4) |  |  |
| Caucasian | 5 (0.2) | 1 (0.3) | 3 (0.2) | 1 (0.4) |  |  |
| Others | 72 (3.5) | 12 (4.0) | 50 (3.4) | 10 (3.7) |  |  |
| **Citizenship, *n* (%)** |  |  |  |  |  |  |
| Singapore citizen | 1819 (89.2) | 264 (87.7) | 1312 (89.5) | 243 (89.0) | 2.4 | 0.654 |
| Singapore Permanent Resident | 88 (4.3) | 15 (5.0) | 58 (4.0) | 15 (5.5) |  |  |
| Foreigner | 133 (6.5) | 22 (7.3) | 96 (6.5) | 15 (5.5) |  |  |
| **Subjective social status, *n* (%)** |  |  |  |  |  |  |
| Low | 353 (17.3) | 30 (10.0) | 245 (16.7) | 78 (28.6) | 42.0 | <0.001 |
| Moderate | 1489 (73.0) | 229 (76.1) | 1081 (73.7) | 179 (65.6) |  |  |
| High | 198 (9.7) | 42 (14.0) | 140 (9.5) | 16 (5.9) |  |  |
| **Year of study *n* (%)** |  |  |  |  |  |  |
| Year 1 | 741 (36.3) | 115 (38.2) | 544 (37.1) | 82 (30.0) | 17.2 | 0.028 |
| Year 2 | 687 (33.7) | 97 (32.2) | 491 (33.5) | 99 (36.3) |  |  |
| Year 3 | 189 (9.3) | 15 (5.0) | 145 (9.9) | 29 (10.6) |  |  |
| Year 4 | 390 (19.1) | 66 (21.9) | 267 (18.2) | 57 (20.9) |  |  |
| Year 5 | 33 (1.6) | 8 (2.7) | 19 (1.3) | 6 (2.2) |  |  |
| **Chronic medical issue, *n* (%)** | 80 (3.9) | 3 (1.0) | 54 (3.7) | 23 (8.4) | 21.7 | <0.001 |
| **Mental health care, n (%)** | 78 (3.8) | 6 (2.0) | 49 (3.3) | 23 (8.5) | 19.5 | <0.001 |
| **Self-rated health, *n* (%)** |  |  |  |  |  |  |
| Excellent | 251 (12.3) | 114 (37.9) | 133 (9.1) | 4 (1.5) | 778.4 | <0.001 |
| Good | 1069 (52.4) | 178 (59.1) | 843 (57.5) | 48 (17.6) |  |  |
| Fair | 647 (31.7) | 9 (3.0) | 483 (32.9) | 155 (56.8) |  |  |
| Poor | 73 (3.6) | 0 (0.0) | 7 (0.5) | 66 (24.2) |  |  |
| **Sleep quality, *n* (%)** |  |  |  |  |  |  |
| Good | 470 (23.0) | 256 (85.0) | 213 (14.5) | 1 (0.4) | 1468.1 | <0.001 |
| Okay | 1203 (59.0) | 45 (15.0) | 1089 (74.3) | 69 (25.3) |  |  |
| Bad | 311 (15.2) | 0 (0.0) | 159 (10.8) | 152 (55.7) |  |  |
| Very bad | 56 (2.7) | 0 (0.0) | 5 (0.3) | 51 (18.7) |  |  |
| **Sleepiness problem, *n* (%)** |  |  |  |  |  |  |
| No problem at all | 419 (20.5) | 155 (51.5) | 256 (17.5) | 8 (2.9) | 692.1 | <0.001 |
| A little problem | 1245 (61.0) | 144 (47.8) | 1004 (68.5) | 97 (35.5) |  |  |
| More than a little problem | 302 (14.8) | 2 (0.7) | 192 (13.1) | 108 (39.6) |  |  |
| A big problem | 58 (2.8) | 0 (0.0) | 13 (0.9) | 45 (16.5) |  |  |
| A very big problem | 16 (0.8) | 0 (0.0) | 1 (0.1) | 15 (5.5) |  |  |
| **Stayed up past 3:00 am, *n* (%)** |  |  |  |  |  |  |
| Never | 655 (32.1) | 211 (70.1) | 423 (28.9) | 21 (7.7) | 381.7 | <0.001 |
| Once or twice | 733 (35.9) | 78 (25.9) | 582 (39.7) | 73 (26.7) |  |  |
| Several times | 652 (32.0) | 12 (4.0) | 461 (31.4) | 179 (65.6) |  |  |
| **Sleep latency, *n* (%)** |  |  |  |  |  |  |
| Less than 30 minutes | 1454 (71.3) | 282 (93.7) | 1072 (73.1) | 100 (36.6) | 362.1 | <0.001 |
| 30 to 60 minutes | 515 (25.2) | 19 (6.3) | 374 (25.5) | 122 (44.7) |  |  |
| More than 60 minutes | 71 (3.5) | 0 (0.0) | 20 (1.4) | 51 (18.7) |  |  |
| **School day naps, *n* (%)** |  |  |  |  |  |  |
| I never nap | 715 (35.0) | 144 (47.8) | 505 (34.4) | 66 (24.2) | 40.3 | <0.001 |
| 1-2 schooldays/ week | 897 (44.0) | 112 (37.2) | 656 (44.7) | 129 (47.3) |  |  |
| 3-4 school days/ week | 282 (13.8) | 31 (10.3) | 200 (13.6) | 51 (18.7) |  |  |
| Every school day | 146 (7.2) | 14 (4.7) | 105 (7.2) | 27 (9.9) |  |  |
| **Weekend naps, *n* (%)** |  |  |  |  |  |  |
| I don’t nap | 1053 (51.6) | 180 (59.8) | 739 (50.4) | 134 (49.1) | 13.0 | 0.012 |
| Once | 734 (36.0) | 93 (30.9) | 546 (37.2) | 95 (34.8) |  |  |
| Twice or more | 253 (12.4) | 28 (9.3) | 181 (12.3) | 44 (16.1) |  |  |
| **Caffeine to stay awake, *n* (%)** |  |  |  |  |  |  |
| No | 1231 (60.3) | 209 (69.4) | 872 (59.5) | 150 (54.9) | 14.2 | 0.001 |
| Yes | 809 (39.7) | 92 (30.6) | 594 (40.5) | 123 (45.1) |  |  |
| **Self-rated chronotype (%)** |  |  |  |  |  |  |
| Morning type | 262 (12.8) | 72 (23.9) | 169 (11.5) | 21 (7.7) | 79.9 | <0.001 |
| Intermediate type | 1284 (62.9) | 195 (64.8) | 939 (64.1) | 150 (54.9) |  |  |
| Evening type | 494 (24.2) | 34 (11.3) | 358 (24.4) | 102 (37.4) |  |  |
| **Sleep on school days** |  |  |  |  |  |  |
| Bedtime (hh:mm) | 01:15 ± 01:23 | 00:20 ± 01:07 | 01:17 ± 01:19 | 02:01 ± 01:26 | 122.1 | <0.001 |
| Wake-up time (hh:mm) | 08:40 ± 01:25 | 08:08 ± 01:13 | 08:41 ± 01:22 | 09:05 ± 01:40 | 33.2 | <0.001 |
| Nocturnal sleep duration (h) | 6.99 ± 1.09 | 7.57 ± 0.88 | 6.97 ± 1.05 | 6.47 ± 1.18 | 81.0 | <0.001 |
| **Sleep on weekends** |  |  |  |  |  |  |
| Bedtime (hh:mm) | 01:37 ± 01:31 | 00:48 ± 01:12 | 01:38 ± 01:27 | 02:23 ± 01:40 | 86.0 | <0.001 |
| Wake-up time (hh:mm) | 09:58 ± 01:43 | 09:10 ± 01:25 | 09:59 ± 01:37 | 10:44 ± 02:01 | 64.4 | <0.001 |
| Nocturnal sleep duration (h) | 8.13 ± 1.15 | 8.22 ± 0.97 | 8.13 ± 1.16 | 8.03 ± 1.30 | 2.0 | 0.142 |
| Midpoint of sleep (hh:mm) | 05:47 ± 01:29 | 04:59 ± 01:13 | 05:49 ± 01:25 | 06:34 ± 01:43 | 87.4 | <0.001 |

**Supplementary Table 1d: Study 4 in university freshmen (prospective study, 2020 cohort)**

|  |  | **SHAWQ category of sleep health** | | |  |  |
| --- | --- | --- | --- | --- | --- | --- |
| **Characteristic** | **All (n = 1529)** | **Good**  **(n = 176)** | **Fair**  **(n = 946)** | **Bad**  **(n = 407)** | ***X*^2^*/F*** | ***p*** |
| **Girls, *n* (%)** | 827 (54.1) | 66 (37.5) | 516 (54.5) | 245 (60.2) | 25.7 | <0.001 |
| **Age (years)** | 20.55 ± 1.50 | 20.74 ± 1.22 | 20.52 ± 1.60 | 20.53 ± 1.38 | 1.6 | 0.196 |
| **Ethnicity, *n* (%)** |  |  |  |  |  |  |
| Chinese | 1324 (86.6) | 153 (86.9) | 825 (87.2) | 346 (85.0) | 2.4 | 0.879 |
| Malay | 30 (2.0) | 3 (1.7) | 20 (2.1) | 7 (1.7) |  |  |
| Indian | 87 (5.7) | 11 (6.2) | 49 (5.2) | 27 (6.6) |  |  |
| Others | 88 (5.8) | 9 (5.1) | 52 (5.5) | 27 (6.6) |  |  |
| **Citizenship, *n* (%)** |  |  |  |  |  |  |
| Singapore citizen | 1352 (88.4) | 159 (90.3) | 841 (88.9) | 352 (86.5) | 8.9 | 0.064 |
| Singapore Permanent Resident | 54 (3.5) | 9 (5.1) | 25 (2.6) | 20 (4.9) |  |  |
| Foreigner | 123 (8.0) | 8 (4.5) | 80 (8.5) | 35 (8.6) |  |  |
| **Housing type, *n* (%)** |  |  |  |  |  |  |
| Public 1- or 2-room flat | 16 (1.1) | 1 (0.6) | 10 (1.1) | 5 (1.2) | 7.1 | 0.714 |
| Public 3-room flat | 125 (8.2) | 10 (5.7) | 73 (7.8) | 42 (10.4) |  |  |
| Public 4-room flat | 355 (23.3) | 43 (24.4) | 217 (23.1) | 95 (23.5) |  |  |
| Public Executive/ 5-room flat | 454 (29.8) | 56 (31.8) | 277 (29.4) | 121 (30.0) |  |  |
| Condo/ Private | 381 (25.0) | 46 (26.1) | 246 (26.1) | 89 (22.0) |  |  |
| Landed property | 190 (12.5) | 20 (11.4) | 118 (12.5) | 52 (12.9) |  |  |
| **School of enrolment, *n* (%)** |  |  |  |  |  |  |
| Faculty of Science | 203 (13.3) | 21 (11.9) | 131 (13.8) | 51 (12.5) | 37.8 | 0.064 |
| Business School | 224 (14.7) | 21 (11.9) | 135 (14.3) | 68 (16.7) |  |  |
| Fac. of Arts & Social Sciences | 316 (20.7) | 33 (18.8) | 196 (20.7) | 87 (21.4) |  |  |
| School of Computing | 200 (13.1) | 23 (13.1) | 122 (12.9) | 55 (13.5) |  |  |
| Faculty of Engineering | 199 (13.0) | 30 (17.0) | 121 (12.8) | 48 (11.8) |  |  |
| School of Design & Environment | 108 (7.1) | 8 (4.5) | 54 (5.7) | 46 (11.3) |  |  |
| Nursing Studies | 87 (5.7) | 13 (7.4) | 57 (6.0) | 17 (4.2) |  |  |
| Faculty of Law | 36 (2.4) | 3 (1.7) | 27 (2.9) | 6 (1.5) |  |  |
| School of Medicine | 37 (2.4) | 7 (4.0) | 25 (2.6) | 5 (1.2) |  |  |
| Yale-NUS College | 46 (3.0) | 9 (5.1) | 31 (3.3) | 6 (1.5) |  |  |
| Multidisciplinary Degree | 17 (1.1) | 2 (1.1) | 11 (1.2) | 4 (1.0) |  |  |
| Faculty of Dentistry | 14 (0.9) | 2 (1.1) | 9 (1.0) | 3 (0.7) |  |  |
| School of Music | 2 (0.1) | 0 (0.0) | 2 (0.2) | 0 (0.0) |  |  |
| Others | 40 (2.6) | 4 (2.3) | 25 (2.6) | 11 (2.7) |  |  |
| **Self-rated health, *n* (%)** |  |  |  |  |  |  |
| Excellent | 170 (11.1) | 83 (47.2) | 82 (8.7) | 5 (1.2) | 567.3 | <0.001 |
| Good | 713 (46.6) | 87 (49.4) | 525 (55.5) | 101 (24.8) |  |  |
| Fair | 556 (36.4) | 6 (3.4) | 328 (34.7) | 222 (54.5) |  |  |
| Poor | 90 (5.9) | 0 (0.0) | 11 (1.2) | 79 (19.4) |  |  |
| **Sleep quality, *n* (%)** |  |  |  |  |  |  |
| Good | 268 (17.5) | 153 (86.9) | 115 (12.2) | 0 (0.0) | 1354.3 | <0.001 |
| Okay | 801 (52.4) | 23 (13.1) | 704 (74.4) | 74 (18.2) |  |  |
| Bad | 351 (23.0) | 0 (0.0) | 125 (13.2) | 226 (55.5) |  |  |
| Very bad | 109 (7.1) | 0 (0.0) | 2 (0.2) | 107 (26.3) |  |  |
| **Sleepiness problem, *n* (%)** |  |  |  |  |  |  |
| No problem at all | 300 (19.6) | 105 (59.7) | 169 (17.9) | 26 (6.4) | 588.6 | <0.001 |
| A little problem | 791 (51.7) | 70 (39.8) | 601 (63.5) | 120 (29.5) |  |  |
| More than a little problem | 277 (18.1) | 1 (0.6) | 149 (15.8) | 127 (31.2) |  |  |
| A big problem | 137 (9.0) | 0 (0.0) | 25 (2.6) | 112 (27.5) |  |  |
| A very big problem | 24 (1.6) | 0 (0.0) | 2 (0.2) | 22 (5.4) |  |  |
| **Stayed up past 3:00 am, *n* (%)** |  |  |  |  |  |  |
| Never | 406 (26.6) | 122 (69.3) | 268 (28.3) | 16 (3.9) | 349.7 | <0.001 |
| Once or twice | 441 (28.8) | 41 (23.3) | 308 (32.6) | 92 (22.6) |  |  |
| Several times | 682 (44.6) | 13 (7.4) | 370 (39.1) | 299 (73.5) |  |  |
| **Sleep latency, *n* (%)** |  |  |  |  |  |  |
| Less than 30 minutes | 909 (59.5) | 164 (93.2) | 598 (63.2) | 147 (36.1) | 270.2 | <0.001 |
| 30 to 60 minutes | 476 (31.1) | 12 (6.8) | 308 (32.6) | 156 (38.3) |  |  |
| More than 60 minutes | 144 (9.4) | 0 (0.0) | 40 (4.2) | 104 (25.6) |  |  |
| **Self-rated chronotype (%)** |  |  |  |  |  |  |
| Morning type | 210 (13.7) | 54 (30.7) | 126 (13.3) | 30 (7.4) | 89.5 | <0.001 |
| Intermediate type | 995 (65.1) | 107 (60.8) | 641 (67.8) | 247 (60.7) |  |  |
| Evening type | 324 (21.2) | 15 (8.5) | 179 (18.9) | 130 (31.9) |  |  |
| **School sleep duration, *n* (%)** |  |  |  |  |  |  |
| Below 7 hours | 798 (52.2) | 44 (25.0) | 461 (48.7) | 293 (72.0) | 126.3 | <0.001 |
| 7 to 9 hours | 692 (45.3) | 122 (69.3) | 466 (49.3) | 104 (25.6) |  |  |
| Above 9 hours | 39 (2.6) | 10 (5.7) | 19 (2.0) | 10 (2.5) |  |  |

**Supplementary Table 1e: Study 4 in university freshmen (prospective study, 2021 cohort)**

|  |  | **SHAWQ category of sleep health** | | |  |  |
| --- | --- | --- | --- | --- | --- | --- |
| **Characteristic** | **All (n = 1488)** | **Good**  **(n = 178)** | **Fair**  **(n = 1017)** | **Bad**  **(n = 293)** | ***X*^2^*/F*** | ***p*** |
| **Girls, *n* (%)** | 831 (55.8) | 89 (50.0) | 567 (55.8) | 175 (59.7) | 4.3 | 0.119 |
| **Age (years)** | 20.46 ± 2.12 | 20.72 ± 3.40 | 20.41 ± 1.89 | 20.46 ± 1.82 | 1.7 | 0.19 |
| **Ethnicity, *n* (%)** |  |  |  |  |  |  |
| Chinese | 1323 (89.0) | 159 (89.3) | 908 (89.4) | 256 (87.4) | 5.2 | 0.513 |
| Malay | 36 (2.4) | 7 (3.9) | 20 (2.0) | 9 (3.1) |  |  |
| Indian | 69 (4.6) | 6 (3.4) | 50 (4.9) | 13 (4.4) |  |  |
| Others | 59 (4.0) | 6 (3.4) | 38 (3.7) | 15 (5.1) |  |  |
| **Citizenship, *n* (%)** |  |  |  |  |  |  |
| Singapore citizen | 1322 (88.8) | 159 (89.3) | 903 (88.8) | 260 (88.7) | 6.3 | 0.18 |
| Singapore Permanent Resident | 75 (5.0) | 4 (2.2) | 58 (5.7) | 13 (4.4) |  |  |
| Foreigner | 91 (6.1) | 15 (8.4) | 56 (5.5) | 20 (6.8) |  |  |
| **Housing type, *n* (%)** |  |  |  |  |  |  |
| Public 1- or 2-room flat | 14 (0.9) | 3 (1.7) | 8 (0.8) | 3 (1.0) | 6.3 | 0.787 |
| Public 3-room flat | 95 (6.4) | 11 (6.2) | 66 (6.5) | 18 (6.2) |  |  |
| Public 4-room flat | 377 (25.5) | 49 (27.7) | 256 (25.3) | 72 (24.9) |  |  |
| Public Executive/ 5-room flat | 469 (31.8) | 54 (30.5) | 320 (31.7) | 95 (32.9) |  |  |
| Condo/ Private | 347 (23.5) | 33 (18.6) | 243 (24.1) | 71 (24.6) |  |  |
| Landed property | 174 (11.8) | 27 (15.3) | 117 (11.6) | 30 (10.4) |  |  |
| **School of enrolment, *n* (%)** |  |  |  |  |  |  |
| Faculty of Science | 340 (22.8) | 47 (26.4) | 235 (23.1) | 58 (19.8) | 27.4 | 0.388 |
| Business School | 220 (14.8) | 16 (9.0) | 158 (15.5) | 46 (15.7) |  |  |
| Fac. of Arts & Social Sciences | 192 (12.9) | 24 (13.5) | 125 (12.3) | 43 (14.7) |  |  |
| School of Computing | 184 (12.4) | 20 (11.2) | 129 (12.7) | 35 (11.9) |  |  |
| Faculty of Engineering | 218 (14.7) | 27 (15.2) | 144 (14.2) | 47 (16.0) |  |  |
| School of Design & Environment | 79 (5.3) | 7 (3.9) | 58 (5.7) | 14 (4.8) |  |  |
| Nursing Studies | 97 (6.5) | 14 (7.9) | 63 (6.2) | 20 (6.8) |  |  |
| Faculty of Law | 40 (2.7) | 7 (3.9) | 24 (2.4) | 9 (3.1) |  |  |
| School of Medicine | 37 (2.5) | 10 (5.6) | 21 (2.1) | 6 (2.0) |  |  |
| Multidisciplinary Degree | 29 (1.9) | 0 (0.0) | 24 (2.4) | 5 (1.7) |  |  |
| Faculty of Dentistry | 15 (1.0) | 2 (1.1) | 10 (1.0) | 3 (1.0) |  |  |
| College of Humanities & Sci. | 5 (0.3) | 0 (0.0) | 3 (0.3) | 2 (0.7) |  |  |
| School of Music | 2 (0.1) | 0 (0.0) | 2 (0.2) | 0 (0.0) |  |  |
| Others | 30 (2.0) | 4 (2.2) | 21 (2.1) | 5 (1.7) |  |  |
| **Self-rated health, *n* (%)** |  |  |  |  |  |  |
| Excellent | 155 (10.4) | 61 (34.3) | 90 (8.8) | 4 (1.4) | 445.1 | <0.001 |
| Good | 778 (52.3) | 110 (61.8) | 598 (58.8) | 70 (23.9) |  |  |
| Fair | 488 (32.8) | 7 (3.9) | 318 (31.3) | 163 (55.6) |  |  |
| Poor | 67 (4.5) | 0 (0.0) | 11 (1.1) | 56 (19.1) |  |  |
| **Sleep quality, *n* (%)** |  |  |  |  |  |  |
| Good | 307 (20.6) | 162 (91.0) | 141 (13.9) | 4 (1.4) | 1146.8 | <0.001 |
| Okay | 869 (58.4) | 16 (9.0) | 770 (75.7) | 83 (28.3) |  |  |
| Bad | 266 (17.9) | 0 (0.0) | 106 (10.4) | 160 (54.6) |  |  |
| Very bad | 46 (3.1) | 0 (0.0) | 0 (0.0) | 46 (15.7) |  |  |
| **Sleepiness problem, *n* (%)** |  |  |  |  |  |  |
| No problem at all | 337 (22.6) | 113 (63.5) | 207 (20.4) | 17 (5.8) | 502.4 | <0.001 |
| A little problem | 798 (53.6) | 64 (36.0) | 638 (62.7) | 96 (32.8) |  |  |
| More than a little problem | 242 (16.3) | 1 (0.6) | 139 (13.7) | 102 (34.8) |  |  |
| A big problem | 94 (6.3) | 0 (0.0) | 32 (3.1) | 62 (21.2) |  |  |
| A very big problem | 17 (1.1) | 0 (0.0) | 1 (0.1) | 16 (5.5) |  |  |
| **Stayed up past 3:00 am, *n* (%)** |  |  |  |  |  |  |
| Never | 462 (31.0) | 131 (73.6) | 313 (30.8) | 18 (6.1) | 312.4 | <0.001 |
| Once or twice | 488 (32.8) | 39 (21.9) | 373 (36.7) | 76 (25.9) |  |  |
| Several times | 538 (36.2) | 8 (4.5) | 331 (32.5) | 199 (67.9) |  |  |
| **Sleep latency, *n* (%)** |  |  |  |  |  |  |
| Less than 30 minutes | 820 (55.1) | 161 (90.4) | 591 (58.1) | 68 (23.2) | 308.5 | <0.001 |
| 30 to 60 minutes | 534 (35.9) | 17 (9.6) | 377 (37.1) | 140 (47.8) |  |  |
| More than 60 minutes | 134 (9.0) | 0 (0.0) | 49 (4.8) | 85 (29.0) |  |  |
| **Self-rated chronotype (%)** |  |  |  |  |  |  |
| Morning type | 199 (13.4) | 52 (29.2) | 129 (12.7) | 18 (6.1) | 76.7 | <0.001 |
| Intermediate type | 1011 (67.9) | 110 (61.8) | 713 (70.1) | 188 (64.2) |  |  |
| Evening type | 278 (18.7) | 16 (9.0) | 175 (17.2) | 87 (29.7) |  |  |
| **School sleep duration, *n* (%)** |  |  |  |  |  |  |
| Below 7 hours | 586 (39.4) | 37 (20.8) | 373 (36.7) | 176 (60.1) | 87.2 | <0.001 |
| 7 to 9 hours | 833 (56.0) | 124 (69.7) | 600 (59.0) | 109 (37.2) |  |  |
| Above 9 hours | 69 (4.6) | 17 (9.6) | 44 (4.3) | 8 (2.7) |  |  |

**Supplementary Table 2: Effect sizes of SHAWQ category on mental health, sleep problems, and academic achievement**

|  | **Comparisons between SHAWQ sleep health categories** | | | |
| --- | --- | --- | --- | --- |
|  | **Fair versus Good** | | **Bad versus Good** | |
|  | **Cohen’s d (95% CI)** | ***p*** | **Cohen’s d (95% CI)** | ***p*** |
| *Study 1 in adolescents (analysis of previous study)* | | | | |
| KADS score | 0.92 (0.82 to 1.02) | <0.001 | 2.32 (2.06 to 2.58) | <0.001 |
| *Study 2 in adolescents (cross-sectional study)* | | | | |
| KADS score* | 0.80 (0.66 to 0.93) | <0.001 | 1.83 (1.63 to 2.02) | <0.001 |
| ESS | 0.51 (0.35 to 0.66) | <0.001 | 0.96 (0.78 to 1.13) | <0.001 |
| *Study 3 in university students (cross-sectional study)* | | | | |
| PSQI | 0.99 (0.87 to 1.10) | <0.001 | 2.30 (2.09 to 2.50) | <0.001 |
| ISI | 1.08 (0.97 to 1.18) | <0.001 | 2.61 (2.34 to 2.88) | <0.001 |
| CESDR | 0.72 (0.64 to 0.80) | <0.001 | 1.91 (1.73 to 2.09) | <0.001 |
| CESA | 0.48 (0.38 to 0.58) | <0.001 | 1.09 (0.93 to 1.25) | <0.001 |
| *Study 4 in university freshmen (prospective study)* | | | | |
| 2020 GPA | -0.05 (-0.21 to 0.13) | 0.517 | -0.25 (-0.41 to -0.06) | 0.008 |
| 2020 Percentile Rank | -0.10 (-0.26 to 0.07) | 0.249 | -0.29 (-0.47 to -0.10) | 0.002 |
| 2021 GPA | -0.19 (-0.33 to -0.03) | 0.028 | -0.33 (-0.51 to -0.15) | <0.001 |
| 2021 Percentile Rank | -0.18 (-0.34 to -0.02) | 0.029 | -0.32 (-0.52 to -0.13) | 0.001 |

KADS, Kutcher Adolescent Depression Scale; ESS, Epworth Sleepiness Scale; ISI, Insomnia Severity Index; PSQI, Pittsburgh Sleep Quality Index; CESDR, Center for Epidemiologic Studies Depression Scale Revised; CESA, Center for Epidemiologic Studies Anxiety Scale. *The KADS score was based on a 10-item version of the scale in which the question on thoughts of self-harm or suicide was removed.

**Supplementary Table 3: Effect sizes of SHAWQ category on depression symptoms in adolescents and university students**

**Supplementary Table 3a:** Effect sizes for SHAWQ category in adolescents

|  | **Comparisons between SHAWQ sleep health categories** | | | |
| --- | --- | --- | --- | --- |
|  | **Fair versus Good** | | **Bad versus Good** | |
|  | **Cliff’s delta (95% CI)** | ***p*** | **Cliff’s delta (95% CI)** | ***p*** |
| *Study 1 in adolescents (analysis of previous study)* | | | | |
| Fatigue/low motivation | 0.35 (0.30 to 0.40) | <0.001 | 0.72 (0.64 to 0.77) | <0.001 |
| Lack of Focus | 0.30 (0.25 to 0.35) | <0.001 | 0.64 (0.55 to 0.71) | <0.001 |
| Worthlessness | 0.31 (0.26 to 0.36) | <0.001 | 0.63 (0.55 to 0.70) | <0.001 |
| Sadness | 0.30 (0.25 to 0.35) | <0.001 | 0.63 (0.55 to 0.70) | <0.001 |
| Life is not fun/anhedonia | 0.27 (0.22 to 0.32) | <0.001 | 0.56 (0.47 to 0.63) | <0.001 |
| Physical signs of anxiety | 0.21 (0.16 to 0.25) | <0.001 | 0.53 (0.44 to 0.61) | <0.001 |
| Anxiety | 0.25 (0.20 to 0.30) | <0.001 | 0.52 (0.43 to 0.60) | <0.001 |
| Sleep difficulties | 0.23 (0.18 to 0.28) | <0.001 | 0.47 (0.38 to 0.56) | <0.001 |
| Apathy | 0.26 (0.21 to 0.31) | <0.001 | 0.46 (0.36 to 0.54) | <0.001 |
| Irritability | 0.20 (0.15 to 0.25) | <0.001 | 0.42 (0.33 to 0.51) | <0.001 |
| Self-harm thoughts | 0.10 (0.07 to 0.13) | <0.001 | 0.34 (0.26 to 0.42) | <0.001 |
| *Study 2 in adolescents (cross-sectional study)* | | | | |
| Fatigue/low motivation | 0.33 (0.26 to 0.40) | <0.001 | 0.67 (0.60 to 0.73) | <0.001 |
| Lack of Focus | 0.27 (0.19 to 0.33) | <0.001 | 0.57 (0.49 to 0.63) | <0.001 |
| Worthlessness | 0.32 (0.24 to 0.38) | <0.001 | 0.62 (0.54 to 0.68) | <0.001 |
| Sadness | 0.28 (0.20 to 0.35) | <0.001 | 0.59 (0.51 to 0.65) | <0.001 |
| Life is not fun/anhedonia | 0.24 (0.16 to 0.32) | <0.001 | 0.55 (0.48 to 0.63) | <0.001 |
| Physical signs of anxiety | 0.28 (0.21 to 0.34) | <0.001 | 0.55 (0.47 to 0.61) | <0.001 |
| Anxiety | 0.21 (0.14 to 0.28) | <0.001 | 0.51 (0.43 to 0.58) | <0.001 |
| Sleep difficulties | 0.37 (0.30 to 0.43) | <0.001 | 0.74 (0.68 to 0.79) | <0.001 |
| Apathy | 0.26 (0.18 to 0.33) | <0.001 | 0.47 (0.39 to 0.55) | <0.001 |
| Irritability | 0.16 (0.08 to 0.23) | <0.001 | 0.46 (0.38 to 0.53) | <0.001 |

**Supplementary Table 3b:** Effect sizes for SHAWQ category in university students

|  |  | **Comparisons between SHAWQ sleep health categories** | | | |
| --- | --- | --- | --- | --- | --- |
|  |  | **Fair versus Good** | | **Bad versus Good** | |
| *Study 3 in university students* (cross-sectional study) | | **Cliff’s delta (95% CI)** | ***p*** | **Cliff’s delta (95% CI)** | ***p*** |
| CESDR | Fatigue: Tired all the time | 0.36 (0.30 to 0.41) | <0.001 | 0.76 (0.70 to 0.81) | <0.001 |
|  | Sleep: Restless sleep | 0.32 (0.27 to 0.36) | <0.001 | 0.72 (0.66 to 0.77) | <0.001 |
|  | Thinking: Cannot focus | 0.32 (0.26 to 0.38) | <0.001 | 0.72 (0.65 to 0.77) | <0.001 |
|  | Thinking: Mind wanders | 0.34 (0.27 to 0.40) | <0.001 | 0.70 (0.64 to 0.76) | <0.001 |
|  | Fatigue: Cannot get going | 0.34 (0.28 to 0.39) | <0.001 | 0.68 (0.61 to 0.74) | <0.001 |
|  | Movement: Feel slow-moving | 0.30 (0.25 to 0.35) | <0.001 | 0.65 (0.58 to 0.71) | <0.001 |
|  | Dysphoria: Feel the blues | 0.29 (0.23 to 0.34) | <0.001 | 0.64 (0.57 to 0.70) | <0.001 |
|  | Dysphoria: Feel depressed | 0.26 (0.21 to 0.31) | <0.001 | 0.61 (0.55 to 0.68) | <0.001 |
|  | Sleep: Trouble getting to sleep | 0.22 (0.17 to 0.26) | <0.001 | 0.58 (0.52 to 0.65) | <0.001 |
|  | Guilt: Dislike myself | 0.25 (0.20 to 0.30) | <0.001 | 0.58 (0.50 to 0.64) | <0.001 |
|  | Dysphoria: Feel sad | 0.26 (0.20 to 0.32) | <0.001 | 0.58 (0.50 to 0.64) | <0.001 |
|  | Anhedonia: No happiness | 0.20 (0.15 to 0.25) | <0.001 | 0.55 (0.48 to 0.62) | <0.001 |
|  | Movement: Feel fidgety | 0.25 (0.20 to 0.31) | <0.001 | 0.53 (0.45 to 0.60) | <0.001 |
|  | Anhedonia: Lost interest | 0.21 (0.15 to 0.26) | <0.001 | 0.51 (0.43 to 0.58) | <0.001 |
|  | Guilt: Feel like a bad person | 0.19 (0.14 to 0.24) | <0.001 | 0.50 (0.42 to 0.57) | <0.001 |
|  | Sleep: Slept much more | 0.26 (0.20 to 0.31) | <0.001 | 0.48 (0.40 to 0.55) | <0.001 |
|  | Appetite: Poor Appetite | 0.25 (0.20 to 0.29) | <0.001 | 0.46 (0.39 to 0.53) | <0.001 |
|  | Suicide: Wish to be dead | 0.14 (0.10 to 0.16) | <0.001 | 0.36 (0.29 to 0.42) | <0.001 |
|  | Suicide: Want to hurt myself | 0.08 (0.06 to 0.10) | <0.001 | 0.22 (0.16 to 0.27) | <0.001 |
|  | Appetite: Lost weight w/o reason | 0.06 (0.02 to 0.08) | 0.001 | 0.18 (0.13 to 0.24) | <0.001 |
| KADS | Fatigue/low motivation | 0.35 (0.29 to 0.40) | <0.001 | 0.72 (0.67 to 0.77) | <0.001 |
|  | Sadness | 0.31 (0.24 to 0.37) | <0.001 | 0.62 (0.55 to 0.68) | <0.001 |
|  | Lack of focus | 0.29 (0.23 to 0.35) | <0.001 | 0.63 (0.56 to 0.69) | <0.001 |
|  | Anxiety | 0.23 (0.17 to 0.29) | <0.001 | 0.55 (0.48 to 0.62) | <0.001 |
| *Study 4 in university freshmen (cross-sectional data)* | | **Cliff’s delta (95% CI)** | ***p*** | **Cliff’s delta (95% CI)** | ***p*** |
|  | 2020 Freshmen | | | | |
| KADS | Fatigue/low motivation | 0.41 (0.33 to 0.48) | <0.001 | 0.70 (0.63 to 0.76) | <0.001 |
|  | Sadness | 0.35 (0.26 to 0.43) | <0.001 | 0.60 (0.52 to 0.66) | <0.001 |
|  | Lack of focus | 0.36 (0.27 to 0.43) | <0.001 | 0.58 (0.50 to 0.65) | <0.001 |
|  | Anxiety | 0.26 (0.18 to 0.33) | <0.001 | 0.52 (0.43 to 0.59) | <0.001 |
| QoL | Satisfied with sleep | -0.58 (-0.64 to -0.52) | <0.001 | -0.88 (-0.92 to -0.84) | <0.001 |
|  | Energy for everyday life | -0.33 (-0.41 to -0.25) | <0.001 | -0.63 (-0.69 to -0.56) | <0.001 |
|  | 2021 Freshmen | | | | |
| KADS | Fatigue/low motivation | 0.32 (0.24 to 0.40) | <0.001 | 0.60 (0.52 to 0.67) | <0.001 |
|  | Sadness | 0.29 (0.21 to 0.37) | <0.001 | 0.56 (0.47 to 0.63) | <0.001 |
|  | Lack of focus | 0.21 (0.13 to 0.29) | <0.001 | 0.51 (0.43 to 0.59) | <0.001 |
|  | Anxiety | 0.22 (0.13 to 0.30) | <0.001 | 0.50 (0.41 to 0.58) | <0.001 |
| QoL | Satisfied with sleep | -0.55 (-0.61 to -0.48) | <0.001 | -0.82 (-0.87 to -0.77) | <0.001 |
|  | Energy for everyday life | -0.27 (-0.34 to -0.18) | <0.001 | -0.56 (-0.63 to -0.47) | <0.001 |

KADS, Kutcher Adolescent Depression Scale; CESDR, Center for Epidemiologic Studies Depression Scale Revised; QoL, World Health Organization Quality of Life Assessment.

**Supplementary Table 4: Comparison of 6-item SHAWQ versus 4-item version with sleep-related questions only (sleep quality, daytime sleepiness, frequency of staying up until at least 3:00 am, school day sleep latency)**

|  | **Comparison of associations with sleep & mental health measures** | | | |
| --- | --- | --- | --- | --- |
|  | **6-item SHAWQ** | | **4-item SHAWQ (sleep items only)** | |
| **Instrument** | **Kendall’s 𝜏 (95% CI)** | ***p*** | **Kendall’s 𝜏 (95% CI)** | ***p*** |
| *Study 1 in adolescents (analysis of previous study)* | | | | |
| KADS | 0.406 (0.377 to 0.432) | <0.001 | 0.384 (0.355 to 0.411) | <0.001 |
| 6-item SHAWQ | - | - | 0.851 (0.840 to 0.861) | <0.001 |
| *Study 2 in adolescents (cross-sectional study)* | | | | |
| KADS* | 0.391 (0.361 to 0.417) | <0.001 | 0.376 (0.347 to 0.404) | <0.001 |
| ESS | 0.213 (0.181 to 0.246) | <0.001 | 0.211 (0.178 to 0.244) | <0.001 |
| 6-item SHAWQ | - | - | 0.847 (0.836 to 0.858) | <0.001 |
| *Study 3 in university students (cross-sectional study)* | | | | |
| PSQI | 0.498 (0.473 to 0.522) | <0.001 | 0.504 (0.479 to 0.528) | <0.001 |
| ISI | 0.495 (0.469 to 0.517) | <0.001 | 0.506 (0.481 to 0.528) | <0.001 |
| CESDR | 0.393 (0.366 to 0.419) | <0.001 | 0.366 (0.337 to 0.392) | <0.001 |
| CESA | 0.220 (0.190 to 0.251) | <0.001 | 0.187 (0.156 to 0.219) | <0.001 |
| 6-item SHAWQ | - | - | 0.842 (0.831 to 0.852) | <0.001 |

KADS, Kutcher Adolescent Depression Scale; ESS, Epworth Sleepiness Scale; ISI, Insomnia Severity Index; PSQI, Pittsburgh Sleep Quality Index; CESDR, Center for Epidemiologic Studies Depression Scale Revised; CESA, Center for Epidemiologic Studies Anxiety Scale. *The KADS score was based on a 10-item version of the scale in which the question on thoughts of self-harm or suicide was removed.

**Supplementary Figure 1**


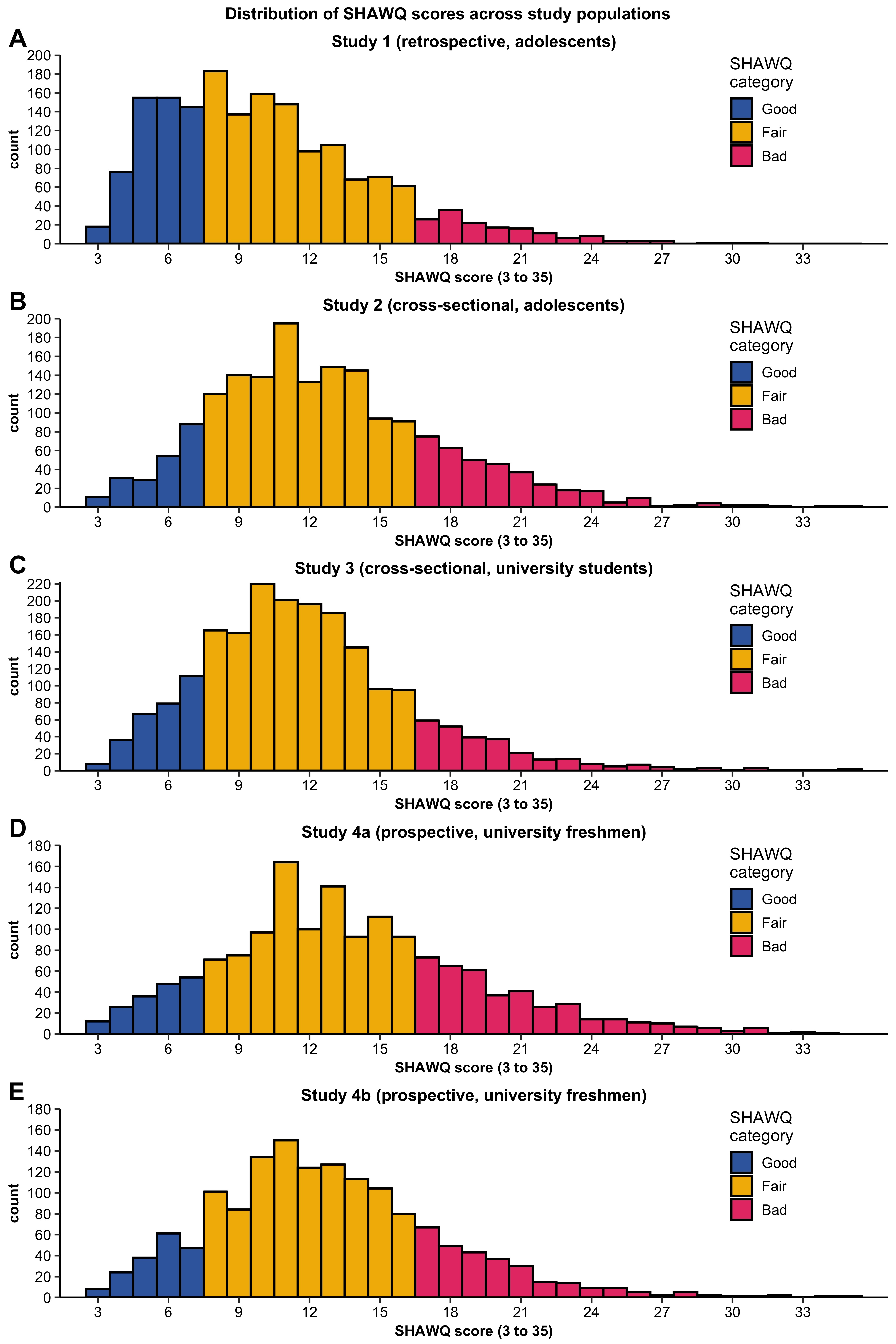


**Supplementary Fig. 1. Distribution of Sleep Health And Wellness Questionnaire (SHAWQ) scores across study populations.** Histograms are color-coded to show students who were categorized as having good, fair, or bad sleep health based on their SHAWQ score. (**a**) The SHAWQ was developed from analysis of a prior study in adolescents (Study 1, *n*=1,733), and then tested subsequently in populations of (**b**) adolescents (Study 2, *n*=1,777), (**c**) university students (Study 3, *n*=2,040), and (**d & e**) 2 cohorts of university freshmen (Study 4, *n*=1,529 and *n*=1,488).

**Supplementary Figure 2**


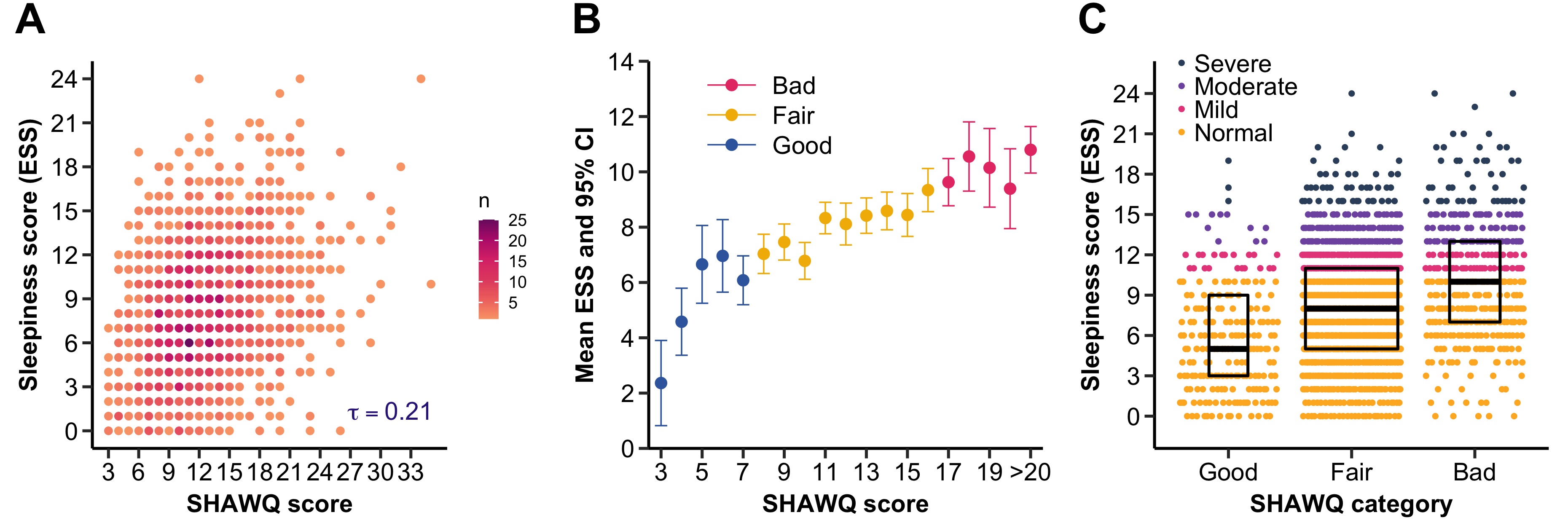


**Supplementary Fig. 2. Sleep Health And Wellness Questionnaire (SHAWQ) scores were associated with excessive daytime sleepiness in adolescents.** Adolescents completed a survey that included the SHAWQ and the Epworth Sleepiness Scale (ESS) (Study 2, *n*=1,777). Students were categorized as having good, fair, or bad sleep health based on their SHAWQ score. (**a**) The scatter density plot shows that ESS scores increased monotonically with higher SHAWQ scores, (**b**) the average ESS score increased by SHAWQ score and category, and (**c**) the distribution of ESS scores and excessive daytime sleepiness categories differed across SHAWQ categories. Box plots show the median and interquartile range.

**Supplementary Figure 3**


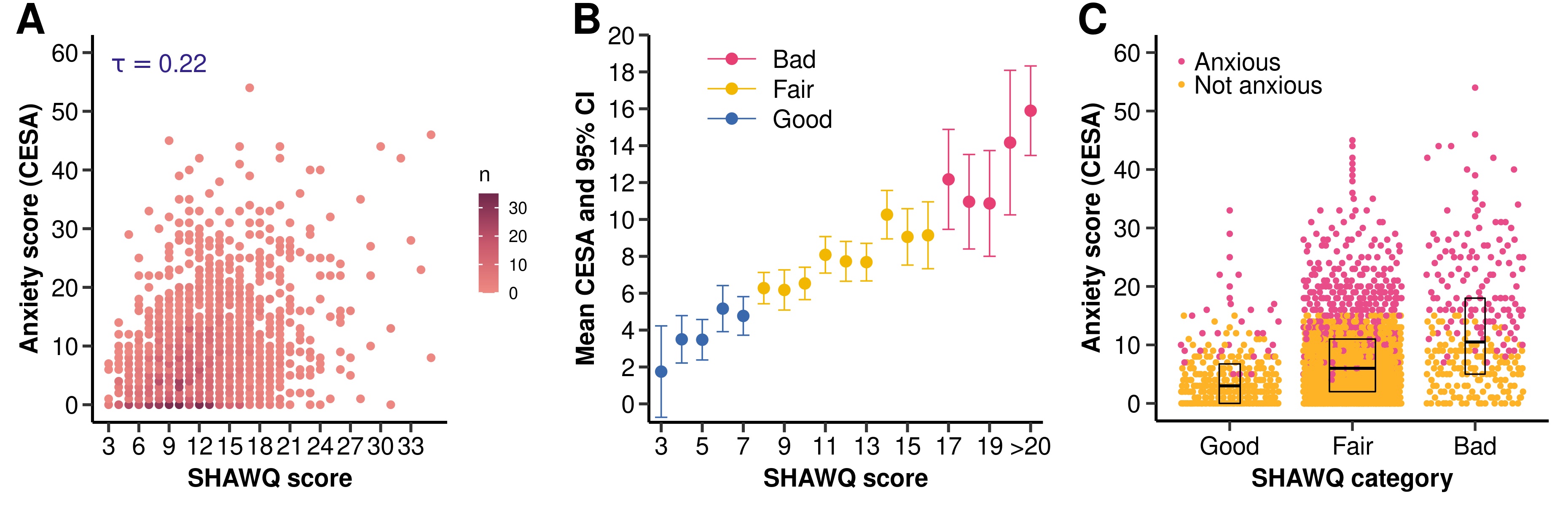


**Supplementary Fig. 3. Sleep Health And Wellness Questionnaire (SHAWQ) scores were associated with anxiety disorder symptoms in university students.** University students completed a survey that included the SHAWQ and the Center for Epidemiologic Studies Anxiety (CESA) scale (Study 3, *n*=2,040). Students were categorized as having good, fair, or bad sleep health based on their SHAWQ score. (**a**) The scatter density plot shows that CESA scores increased with higher SHAWQ scores, (**b**) the average CESA score increased by SHAWQ score and category, and (**c**) the distribution of CESA scores and anxiety categories differed across SHAWQ categories. Box plots show the median and interquartile range.

**Supplementary Figure 4**

**
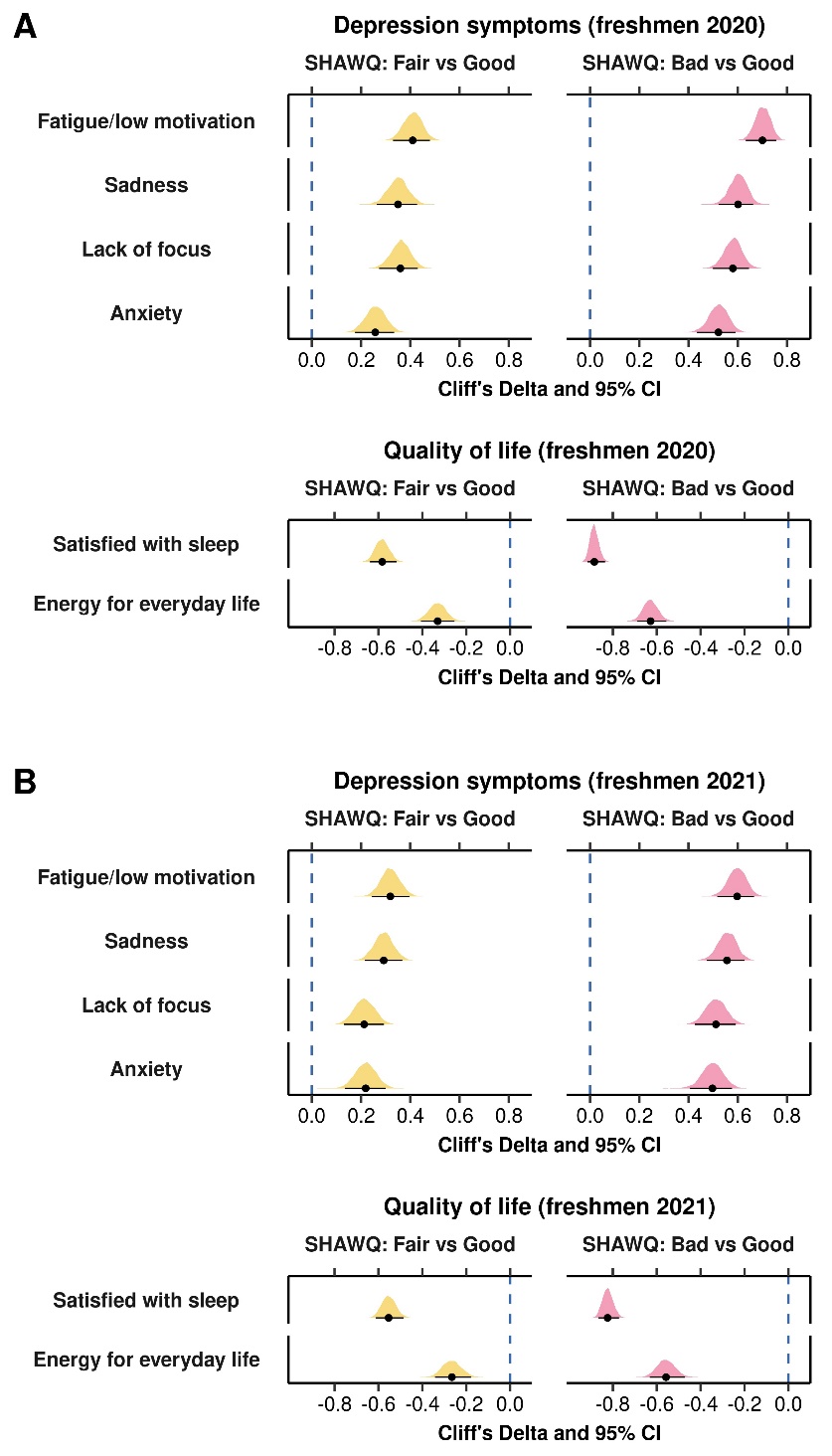
**

**Supplementary Fig. 4. Effect size plots for sleep health and depression symptoms in university freshmen.** Students who enrolled in 2020 (*n*=1,529) and 2021 (*n*=1,488) completed a survey that included the Sleep Health and Wellness Questionnaire (SHAWQ) and a subset of questions from the Kutcher Adolescent Depression Scale and the World Health Organization Quality of Life Assessment. Students were categorized as having good, fair, or bad sleep health based on their SHAWQ score. Effect sizes (Cliff’s delta) for fair and bad sleep health were determined relative to good sleep health. In the (**a**) 2020 freshman cohort, and (**b**) the 2021 freshman cohort, depression symptoms and quality of life measures were worse in the fair sleep health group with small-to-large effect sizes, and worse in the bad sleep health group with large effect sizes. In each plot, the population estimate of effect size is shown with 95% CIs and the bootstrap sampling distribution (5,000 samples).
